# Influenza vaccine protection and contact patterns among outpatients with influenza-like illness during the 2024–2025 season in China: a test-negative case-control study

**DOI:** 10.7189/jogh.16.04253

**Published:** 2026-10-09

**Authors:** Luping Chen, Jia Mi, Yingxuan Wang, Zhisheng Chen, Juping Wang, Zihao Guo, Haogao Gu, Qiaoge Chi, Lirong Cao, Ruijuan Ji, Kamuranni Luotebula, Shengmei Yang, Yan Kong, Kailu Wang, Sheikh Taslim Ali, Daihai He, Marc KC Chong, Shi Zhao, Kai Wang, Yuantao Hao, David SC Hui, Zijian Feng

**Affiliations:** 1 Institute of Medical Engineering Interdisciplinary Research, College of Medical Engineering and Technology, Xinjiang Medical University, Urumqi, Xinjiang, China; 2 School of Public Health, Tianjin Medical University, Tianjin, China; 3 Ili Kazakh Autonomous Prefecture Center for Disease Control and Prevention, Ili Kazakh Autonomous Prefecture, China; 4 JC School of Public Health and Primary Care, The Chinese University of Hong Kong, Hong Kong, China; 5 School of Public Health, The University of Hong Kong, Hong Kong, China; 6 Department of Biomedical Sciences, City University of Hong Kong, Hong Kong, China; 7 Health Commission of Ili Kazakh Autonomous Prefecture, Ili Kazakh Autonomous Prefecture, China; 8 Department of Mathematics, The Hong Kong Polytechnic University, Hong Kong, China; 9 Peking University Center for Public Health and Epidemic Preparedness & Response, MoE Key Laboratory of Epidemiology of Major Diseases, Department of Epidemiology and Biostatistics, School of Public Health, Peking University, Beijing, China; 10 Department of Medicine and Therapeutics, The Chinese University of Hong Kong, Hong Kong, China; 11 School of Public Health and Emergency Management, Southern University of Science and Technology, Shenzhen, China

**Keywords:** influenza, influenza vaccination, vaccine effectiveness, test-negative design, contact patterns, China

## Abstract

**Background:**

Continuous monitoring of both influenza vaccine protection and behavioural determinants of infection is important for understanding related risks. Evidence on influenza vaccine effectiveness (VE) in China during the 2024–2025 season, however, remains limited. We aimed to assess influenza VE among healthcare-seeking outpatients with influenza-like illness (ILI) and to examine the contact patterns associated with infection risk.

**Methods:**

We enrolled 928 outpatients with ILI from three sentinel hospitals in Ili Kazakh Autonomous Prefecture, China, between December 2024 and March 2025 in a test-negative case-control study. We confirmed influenza infection using reverse transcription-polymerase chain reaction and collected vaccination history and social contact frequency and duration through questionnaires. We estimated VE using logistic regression with covariate adjustment, and otherwise analysed social contact patterns and performed exploratory sequencing of six randomly selected influenza-positive samples.

**Results:**

Our full sample comprised 928 participants. We excluded 21 infants younger than 6 months from the VE analysis and included 907 participants, of whom 121 tested positive and 786 tested negative. The overall adjusted VE against medically attended influenza was 42.3% (95% credible interval = 11.1–62.5). In the analysis of social contacts for all 928 participants, test-positive children aged 0–10 years and adults aged 65 years or older reported more frequent and longer social contact than their test-negative counterparts. Exploratory sequencing of six samples suggested no major antigenic mismatch with the vaccine strains, but the very small number of sequenced samples limits the generalisability of this finding.

**Conclusions:**

Influenza vaccination provided moderate protection against medically attended influenza, while contact patterns were associated with infection risk among children and older adults. Combined vaccination and behavioural interventions warrant further evaluation, as the observational design of this study and its use of self-reporting data limit causal inference.

Influenza viruses pose a persistent global health threat due to their role in inducing acute respiratory infections [1,2]. Their high infectivity, short incubation periods, and frequent mutations facilitate rapid transmission and lead to a substantial number of outbreaks annually. The World Health Organization estimates that approximately one billion influenza cases occur each year (*e.g.* pneumonia, bronchitis, which are directly caused by influenza), with 3–5 million being severe and 290,000–650,000 resulting in deaths due to respiratory complications [3,4]. Furthermore, the high rates of human-to-human transmission of influenza viruses, influenced by factors such as population contact density and frequency [5–7], add to this burden, as does their continuous viral evolution. Specifically, influenza viruses evolve through antigenic drift and shift, involving point mutations and gene reassortment [8]. These mechanisms contribute to genetic diversity and can facilitate the emergence of novel pandemic strains, with potentially substantial effects on morbidity and mortality [9–11]. In this context, pathogen genome sequencing provides insights into evolutionary pathways, genetic diversity, and phylogenetic relationships and can support public health surveillance, epidemic preparedness, and the development of control strategies [12–14].

While vaccination is the primary pharmaceutical intervention for preventing influenza [15–17], its effectiveness varies across contexts. Vaccine effectiveness (VE) can be reduced by strain mismatch, host age, immune history, and the circulating virus type or subtype [18]. The influenza vaccine is not included in the national immunisation programme in China, where out-of-pocket payments, low public awareness, and limited availability have contributed to persistently low vaccination coverage [19]. The national vaccination rate averaged 2.43% from 2014 to 2020 and reached only 4% in 2020, compared with 48.4% among adults in the USA during the same period [20,21].

Non-pharmaceutical interventions (NPIs) also play an important role in influenza prevention because the virus is transmitted primarily through respiratory droplets and interpersonal contact. Understanding contact patterns is especially important for estimating transmission dynamics and evaluating NPIs such as school closures and travel restrictions [22,23]. Notably, the type, frequency, duration, and setting of social contacts vary by region and across time. Analyses that account for these variations, particularly by age, sex, and location, can improve infectious disease forecasting and the evaluation of control measures [24–26]. Despite this, studies on contact patterns remain geographically skewed. For example, Mossong *et al.* [27] developed contact matrices for Europe in the Improving Public Health Policy in Europe through Modelling and Economic Evaluation of Interventions for the Control of Infectious Diseases (POLYMOD) study. More recent studies have examined coastal Chinese cities and regions, including Hong Kong, Macao, and Taiwan [28–31]. Evidence remains limited for inland regions, particularly Xinjiang. The Ili Kazakh Autonomous Prefecture in Xinjiang is a multi-ethnic border region with substantial population mobility due to seasonal migration and tourism, which may increase opportunities for influenza transmission. However, few studies have examined social patterns in this region.

We examined influenza transmission in the Ili Kazakh Autonomous Prefecture of Xinjiang by integrating epidemiological, behavioural, and genetic data. We used outpatient samples and questionnaire data from the 2024–2025 influenza season to compare vaccination status and contact patterns between participants who tested positive and those who tested negative for influenza. We also performed exploratory viral genotyping. Previously, we conducted a vaccine effectiveness study in the Ili region in 2023-2024, but that study was limited to evaluating vaccine effectiveness alone; the present study builds on it to provide a more comprehensive understanding of local influenza transmission [32]. Our findings may help identify local transmission drivers, improve outbreak preparedness, and inform regional influenza prevention and control strategies.

## METHODS

### Setting and study design

We conducted a multicentre test-negative case-control study from 8 December 2024 (epidemiological week 50) to 30 March 2025 (epidemiological week 13) among outpatients with influenza-like illness (ILI) from three sentinel hospitals in Yining and Horgos, Ili Kazakh Autonomous Prefecture, China. These sentinel outpatient clinics serve as the core data source for the national influenza surveillance network and support the systematic collection of epidemiological and virological data.

In accordance with the Chinese National Influenza Surveillance Protocol, we defined ILI as the presence of fever (body temperature ≥38°C) accompanied by a cough or sore throat [33]. We randomly selected 10–40 patients (typically 20) with ILI each week from whom we collected nasal or nasopharyngeal specimens for influenza testing using real-time reverse transcription-polymerase chain reaction (RT-PCR), and administered a structured questionnaire to collect demographic information, influenza vaccination history, and social contact patterns. Trained hospital staff administered structured questionnaires and recorded the responses in an electronic database. We linked the questionnaire records to the corresponding laboratory results using unique study identifiers.

We included outpatients who presented with ILI at a sentinel hospital, understood the questionnaire, and provided verbal informed consent, either personally or through a guardian. We excluded participants younger than six months of age from the VE analysis because maternal antibodies could provide protection. We truncated the number of reported daily contact counts above 30 at 30 in the contact analysis to reduce the influence of outliers.

### Outcome variable

We defined the influenza test result as a dichotomous variable, categorised as positive or negative based on RT-PCR analysis of nasopharyngeal swab specimens. We performed testing using the Zhuocheng Huisheng Influenza A/B Duplex real-time PCR detection kit (Beijing Zhuocheng Huisheng Bioscience Co., Ltd., Beijing, China). This kit used specific primers and fluorescent probes targeting influenza A and B viruses and included an internal control that monitors the entire process from nucleic acid extraction to amplification, thereby reducing the risk of false-negative results.

### Exposure variables

We defined influenza vaccination status as the primary exposure variable based on data reported by the participants or their guardians. We classified participants as vaccinated if they had received at least one dose of a seasonal influenza vaccine, including egg-based, cell-based, trivalent, and quadrivalent vaccines, within the past 24 months, with the most recent dose being administered at least 14 days before symptom onset. We classified participants who did not meet these criteria as unvaccinated.

We defined social contact pattern as the secondary exposure variable and characterised it by contact frequency and duration according to the POLYMOD study criteria [22]. We defined conversational contact as a face-to-face conversation with an exchange of at least three words, and physical contact as any skin-to-skin interaction, such as handshaking, hugging, or kissing.

### Covariates

We collected potential confounders using structured questionnaires (Text S1 in the **Online Supplementary Document**), including demographic characteristics such as age, sex, and ethnicity; clinical and behavioural characteristics, such as baseline health conditions, health-related behaviours, and daily habits; and temporal variables, such as the dates of symptom onset and visit dates. We grouped age for further analysis and used symptom onset and visit dates to derive the epidemiological week for adjustment for long-term and seasonal trends.

### Viral sequencing sample selection and reference sequences

The national Centres for Disease Control and Prevention (CDC) influenza surveillance protocol requires sentinel hospitals to perform viral genetic sequencing on a subset of influenza-positive samples during routine surveillance to monitor genetic variation among circulating strains, rather than sequencing all positive samples [33]. We selected 6 out of 121 influenza-positive samples for exploratory sequencing in accordance with this protocol.

We retrieved global influenza A(H1N1) reference sequences from the Global Initiative on Sharing All Influenza Data (GISAID) database [34]. We selected 170 sequences containing both haemagglutinin (HA) and neuraminidase (NA) gene segments and combined them with six viral isolates obtained in this study.

### Statistical analysis

We used a Bayesian logistic regression model adjusted for covariates to estimate VE. We specified an informative prior for the vaccination coefficient, centred on a VE of 40% based on historical CDC estimates [35]. We calculated VE for laboratory-confirmed influenza as (1 − odds ratio) × 100% and expressed uncertainty in the model coefficients using 95% credible intervals (CrIs) derived from the posterior distribution.

We performed targeted sequencing of 6 virus isolates from influenza-positive participants and conducted phylogenetic analysis together with 170 reference sequences retrieved from GISAID. We aligned the sequences using ClustalW in Molecular Evolutionary Genetics Analysis, version 12 [36] and assessed alignment quality using ESPript [37]. We constructed a maximum-likelihood phylogenetic tree using IQ-TREE [38]. We then compared sequences within a specific monophyletic clade to identify variable sites and non-synonymous mutations (Text S3 in the **Online Supplementary Document**).

We performed age standardisation according to the 2020 China Population Census. We used linear regression to quantify age-specific differences in contact frequency and duration (*β*) between influenza test-positive and test-negative participants. We smoothed the contact matrices using Gaussian kernel density estimation. We used stratified χ^2^ tests to compare the distributions of contact setting, frequency, and duration between the groups. We used a Bayesian negative binomial regression model to assess the effects of demographic characteristics and contact setting and evaluated model robustness against a gamma model (Text S4 in the **Online Supplementary Document**).

We conducted subgroup analyses for VE by age group (6 months to 6 years, 7–17 years, 18–59 years, and ≥60 years) and influenza subtype (A(H1), A(H3), and B/Victoria). We performed similar analyses for social contacts by age groups (0–5 years, 6–15 years, 16–65 years, and >65 years) based on immune status, social behaviours, and health risks, with further stratification by sex (male and female), household size (small, ≤3 persons; medium, 4 persons; large, ≥5 persons), and contact setting (home, school, workplace, leisure, and other) (Text S4 in the **Online Supplementary Document**).

We also conducted sensitivity analyses to evaluate the robustness of the VE estimates and social contact analyses (Text S5 in the **Online Supplementary Document**). For the VE analysis, we assessed the effects of including infants younger than six months, treating age and body mass index as continuous variables, and adjusting for the symptom onset date. For the social contact analysis, we restricted the sample to unvaccinated participants to reduce potential confounding by vaccination status.

We performed all statistical analyses and phylogenetic tree visualisations using *R*, version 4.3.2 (R Core Team, Vienna, Austria), with the ‘EBImage’, ‘seqinr’, ‘ape’, ‘phytools’, ‘ggplot2’, ‘treeio’, and ‘ggtree’ packages.

## RESULTS

We enrolled 928 participants with ILI between 8 December 2024 and 30 March 2025. For the VE analysis, we excluded 21 infants younger than 6 months and included 907 participants, of whom 121 (13.3%) tested positive for influenza: 101 (83.5%) had influenza A and 20 (16.5%) had influenza B (**Table 1**). Among influenza-positive participants, 5 of 121 (4.1%) had been vaccinated, compared with 51 of 786 influenza-negative participants (6.5%) (**Table 2**).

**Table 1:** Demographic and clinical characteristics of participants presenting to hospital with influenza-like illness, according to influenza test result*

	Test-positive participants			
	**Influenza A (n = 101)**	**Influenza B (n = 20)**	**Total (n = 121)**	**Test-negative participants (n = 786)**
**Sex**				
Female	51 (50.5)	11 (55.0)	62 (51.2)	405 (51.5)
Male	50 (49.5)	9 (45.0)	59 (48.8)	381 (48.5)
**Age in years**				
x̄ (SD)	37.9 (29.9)	23.8 (21.2)	35.6 (29.0)	25.3 (24.7)
0.5–6	24 (23.8)	6 (30.0)	30 (24.8)	239 (30.4)
7–17	12 (11.9)	3 (15.0)	15 (12.4)	175 (22.3)
18–59	33 (32.7)	9 (45.0)	42 (34.7)	262 (33.3)
≥60	32 (31.7)	2 (10.0)	34 (28.1)	110 (14.0)
**Ethnicity**				
Han	51 (50.5)	5 (25.0)	56 (46.3)	325 (41.3)
Other ethnicities	50 (49.5)	15 (75.0)	65 (53.7)	461 (58.7)
**Comorbidity**				
No comorbidity reported	70 (69.3)	17 (85.0)	87 (71.9)	661 (84.1)
≥1 reported comorbidity	31 (30.7)	3 (15.0)	34 (28.1)	125 (15.9)
**Smoking status**				
Never	86 (85.1)	19 (95.0)	105 (86.8)	663 (84.5)
Former	3 (3.0)	0 (0.0)	3 (2.5)	22 (2.8)
Current	12 (11.9)	1 (5.0)	13 (10.7)	100 (12.7)
**BMI**				
x̄ (SD)	22.6 (4.6)	21.7 (4.3)	22.4 (4.6)	21.0 (4.8)
Underweight (<18.5)	15 (14.9)	6 (30.0)	21 (17.4)	263 (33.5)
Normal (18.5–23.0)	42 (41.6)	5 (25.0)	47 (38.8)	259 (33.0)
Overweight (23.0–27.5)	37 (36.6)	8 (40.0)	45 (37.2)	211 (26.8)
Obesity (>27.5)	7 (6.9)	1 (5.0)	8 (6.6)	53 (6.7)
**Educational attainment**				
Secondary education	63 (62.4)	13 (65.0)	76 (62.8)	548 (69.7)
Post-secondary education	38 (37.6)	7 (35.0)	45 (37.2)	238 (30.3)
**Occupational status**				
Preschool	21 (20.8)	6 (30.0)	27 (22.3)	233 (29.6)
Student	19 (18.8)	3 (15.0)	22 (18.2)	209 (26.6)
Employed	27 (26.7)	6 (30.0)	33 (27.3)	219 (27.9)
Unemployed	19 (18.8)	5 (25.0)	24 (19.8)	70 (8.9)
Retired	15 (14.9)	0 (0.0)	15 (12.4)	53 (6.7)
Not reported	0 (0.0)	0 (0.0)	0 (0.0)	2 (0.3)
**Household size, persons**				
1	2 (2.0)	1 (5.0)	3 (2.5)	10 (1.3)
2	3 (3.0)	0 (0.0)	3 (2.5)	32 (4.1)
3	16 (15.8)	4 (20.0)	20 (16.5)	161 (20.5)
4	38 (37.6)	8 (40.0)	46 (38.0)	312 (39.7)
≥5	42 (41.6)	7 (35.0)	49 (40.5)	271 (34.5)
**Time from ILI onset to specimen collection**				
x̄ (SD)	1.6(1.1)	1.9(1.0)	1.6(1.1)	1.8(1.2)
0–2	92 (91.1)	14 (70.0)	106 (87.6)	630 (80.2)
3–4	6 (5.9)	6 (30.0)	12 (9.9)	138 (17.6)
≥5	3 (3.0)	0 (0.0)	3 (2.5)	18 (2.3)
**Month of ILI onset in the 2024–2025 season**				
December	13 (12.9)	0 (0.0)	13 (10.7)	128 (16.3)
January	29 (28.7)	2 (10.0)	31 (25.6)	226 (28.8)
February	24 (23.8)	3 (15.0)	27 (22.3)	220 (28.0)
March	33 (32.7)	15 (75.0)	48 (39.7)	211 (26.8)
April	2 (2.0)	0 (0.0)	2 (1.7)	1 (0.1)

BMI – body mass index, ILI – influenza-like illness, SD – standard deviation, x̄ – mean

*Presented as n (%) unless specified otherwise.

**Table 2:** Influenza vaccine effectiveness against any influenza, influenza A and influenza B, overall and by age group

	Test-positive participants		Test-negative participants		
	**Total, n**	**Vaccinated, n (%)**	**Total, n**	**Vaccinated, n (%)**	**VE, % (95% CrI)**
**Any influenza**					
Total	121	5 (4.1)	786	51 (6.5)	42.3 (11.1–62.5)
**Age group**					
0.5–6	30	2 (6.7)	239	15 (6.3)	37.5 (−0.5, 61.1)
7–17	15	0 (0.0)	175	13 (7.4)	100 (NE)*
18–59	42	1 (2.4)	262	12 (4.6)	41.1 (5.4–63.3)
≥60	34	2 (5.9)	110	11 (10.0)	42.1 (7.8–63.7)
**Influenza A**					
Total	101	4 (4.0)	786	51 (6.5)	43.1 (11.7, 63.3)
**Age group**					
0.5–6	24	2 (8.3)	239	15 (6.3)	36.5 (−2.3, 60.6)
7–17	12	0 (0.0)	175	13 (7.4)	100 (NE)*
18–59	33	1 (3.0)	262	12 (4.6)	40.4 (4.1–63.0)
≥60	32	1 (3.1)	110	11 (10.0)	44.6 (11.5–65.3)
**Influenza B**					
Total	20	1 (5.0)	786	51 (6.5)	39.0 (1.5, 62.3)
**Age group**					
0.5–6	6	0 (0.0)	239	15 (6.3)	100 (NE)*
7–17	3	0 (0.0)	175	13 (7.4)	100 (NE)*
18–59	9	0 (0.0)	262	12 (4.6)	100 (NE)*
≥60	2	1 (50.0)	110	11 (10.0)	36.9 (−2.7, 61.3)

CrI – credible interval, NE – not estimable, VE – vaccine effectiveness

*The 95% CrIs for VE were not calculated when there were no vaccinated participants in the test-positive group because the statistical estimation failed to converge

Influenza A circulated throughout the study period, from epidemiological week 50 of 2024 to week 13 of 2025. The proportion of participants who tested positive for influenza A increased from 5.3% in week 51 to a peak of 15.6% in week 52 of 2024. Influenza B activity, meanwhile, remained low: it was first detected in week 2 of 2025, with no cases detected in weeks 3, 4, or 7, and with the proportion of positive cases increasing from week 8 and peaking at 6.3% in week 13 (**Figure 1**, Panels E and F).

**Figure 1:**
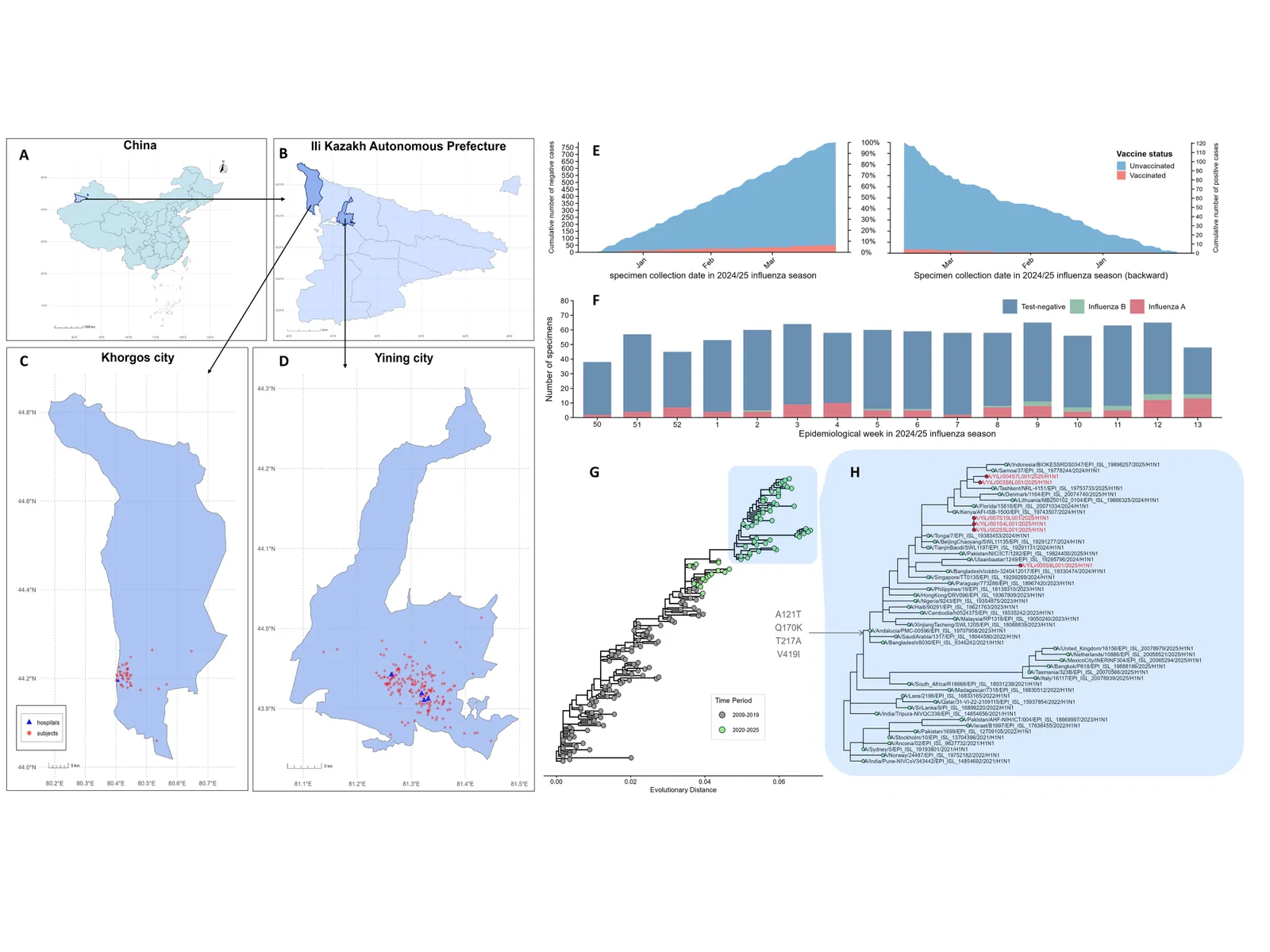
Influenza activity, vaccination, geographic distribution, and phylogenetic analysis of influenza A(H1N1) viruses in Ili Kazakh Autonomous Prefecture, Xinjiang, during the 2024–2025 influenza season. **Panel A.** Map of China showing the location of the study region, Xinjiang. **Panel B.** Map showing the location of Ili Kazakh Autonomous Prefecture within Xinjiang. **Panel C.** Detailed map of the study site in Yining. **Panel D.** Detailed map of the study site in Khorgos. **Panel E.** Weekly enrolment of influenza test-negative and test-positive participants (n = 907) in Ili Kazakh Autonomous Prefecture, China, during the 2024–2025 influenza season. **Panel F.** Cumulative trends of influenza-like illness (blue areas) and influenza vaccination (pink areas) among test-negative and test-positive participants in the left and right plots, respectively. **Panel G.** Phylogenetic tree based on hemagglutinin gene sequences from influenza A(H1N1) viruses; sequences collected from 2020 to 2025 are shown in green, and those collected from 2009 to 2019 shown in grey. **Panel H.** Detailed view of a selected clade comprising 51 sequences. Study sequences are shown in red, and reference sequences are shown in black. The A/Yili/004S7L001/2025/H1N1 strain was collected in week 51 of 2024, whereas the other five study strains were collected in week 50.

The VE against medically attended influenza virus infection among participants with ILI was 42.3% (95% CrI = 11.1–62.5) against both influenza A and B infections, 43.1% (95% CrI = 11.7–63.3) against influenza A specifically, and 39.0% (95% CrI = 1.5–62.3) against influenza B (**Table 2**). Although the point estimate for influenza B suggested a protective trend, the wide CrI indicated limited precision. Age-stratified VE was 37.5% (95% CrI = −0.5, 61.1) among children aged 6 months to 6 years, 41.1% (95% CrI = 5.4–63.3) among adults aged 18–59 years, and 42.1% (95% CrI = 7.8–63.7) among older adults (≥60 years). The CrI for the youngest age group included zero, indicating that the data did not provide clear evidence of vaccine effectiveness in that subgroup. Influenza A similar pattern was observed for influenza A, with the highest VE among older adults, at 44.6% (95% CrI = 11.5–65.3). By virus (sub)type, VE was highest against A(H1N1), at 45.1% (95% CrI = 13.8–65.0), followed by A(H3N2). at 37.8% (95% CrI = 0.2–61.2), and B/Victoria, at 38.3% (95% CrI = −15.7, 67.1).

Phylogenetic analysis of influenza A(H1N1) showed moderate genetic diversity within a genetic background dominated by a single predominant lineage. Within the selected monophyletic clade, we identified clade-specific amino acid substitutions in the HA (A121T, Q170K, T217A, and V419I) and NA (N184S and T248I). These substitutions were not detected in other circulating strains (**Figure 1****,** Panel H; Figure S3 in the **Online Supplementary Document**).

We analysed the average daily number and duration of contacts among all 928 outpatients according to influenza test result and age group. The contact matrices showed strong diagonal patterns in both test groups, indicating age mixing. This pattern was most evident among school-aged children aged 5–15 years. Two secondary diagonals among adults aged 25–30 years suggested frequent intergenerational contact (**Figure 2****,** Panels A, B, D and E). Test-positive participants aged 0–10 years tended to have a higher average daily number of contacts and longer contact durations than their test-negative counterparts (**Figure 2**, Panels C and F). Within outpatient participants, test-positive individuals aged 0 to 10 years and over 65 years exhibited both a higher contact frequency and longer contact duration than their test-negative counterparts did (**Figure 2****,** Panels C and F; **Figure 3****,** Panels A and B). Influenza positivity was highest among children aged 0–5 years and adults older than 70 years (**Figure 3**, Panel C). Age had a stronger positive association with the number of contacts among participants aged 0–5 years (*β* = 1.11) and those older than 65 years (*β* = 1.06) than among those aged 16–65 years (*β* = 0.83) (**Figure 3**, Panel D). The association between age and contact duration showed a similar pattern, with higher coefficients among the youngest (*β* = 1.10) and oldest (*β* = 1.08) age groups (**Figure 3**, Panel E). Associations between contact patterns and infection risk appeard stronger among male participants, among participants from households with at least four members, and for contacts occurring at school and home (Text S4 in the **Online Supplementary Document**). Sensitivity analyses produced similar estimates of VE and social contact patterns (Text S5 in the **Online Supplementary Document**).

**Figure 2:**
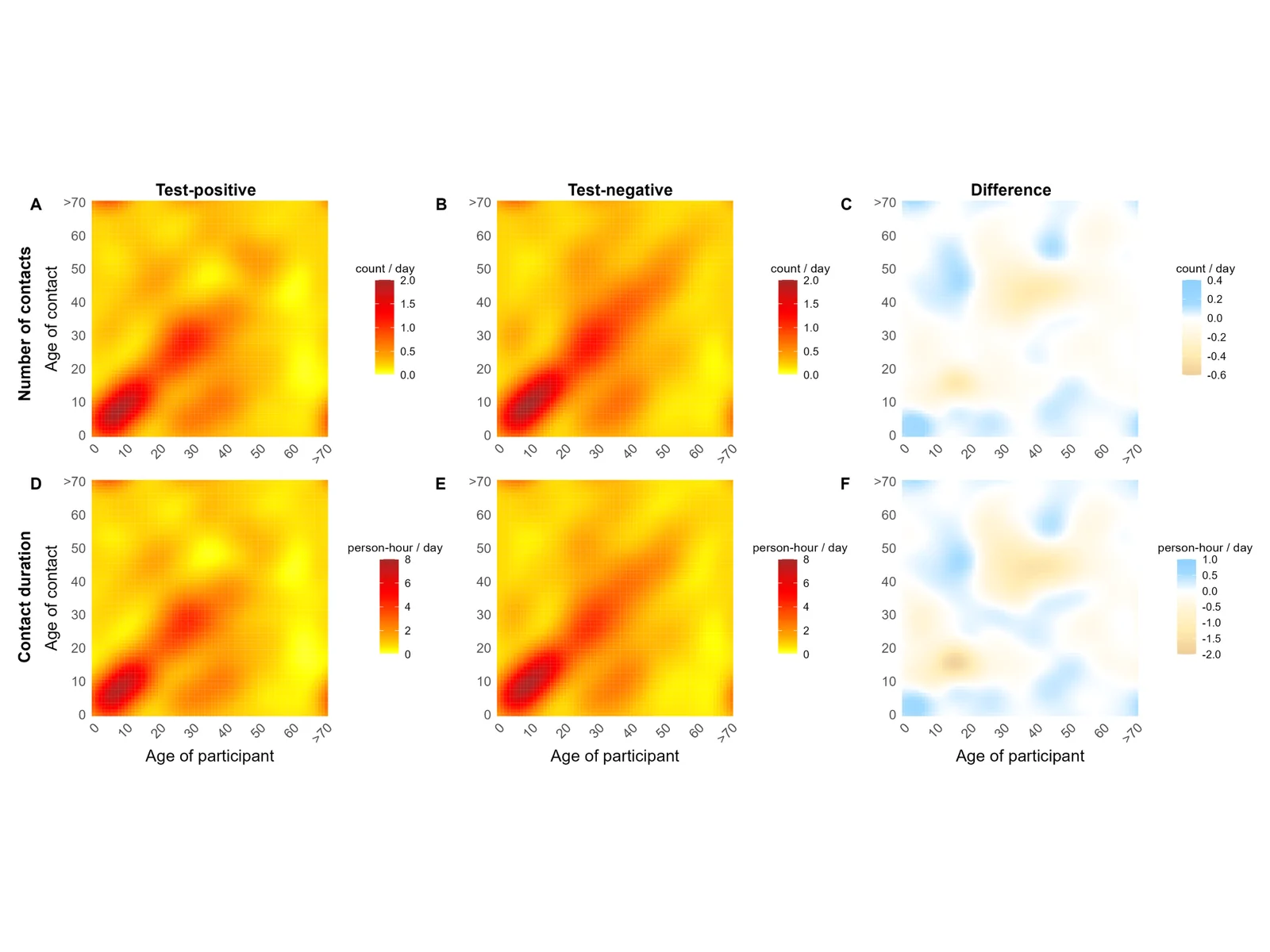
Age-specific contact matrices according to influenza test result. **Panel A.** Number of contacts among influenza test-positive participants. **Panel B.** Number of contacts among influenza test-negative participants. **Panel C.** Difference in the number of contacts between influenza test-positive and test-negative participants. **Panel D.** Total contact duration, expressed in person-hours, among influenza test-positive participants. **Panel E.** Total contact duration, expressed in person-hours, among influenza test-negative participants. **Panel F.** Difference in total contact duration between test-positive and test-negative participants, expressed in person-hours.

**Figure 3:**
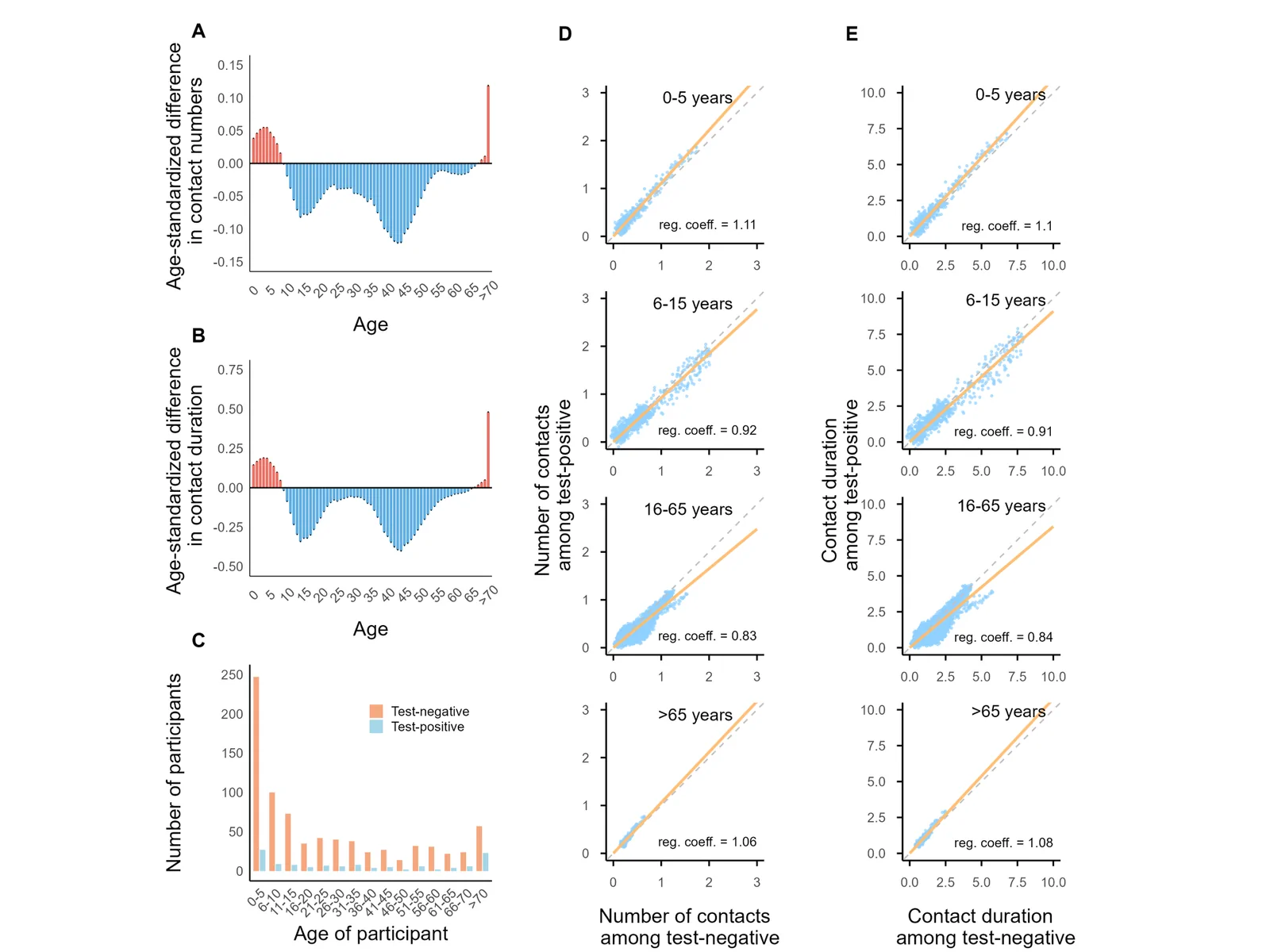
Contact patterns and influenza test results according to age group. **Panel A.** Age-standardised distribution of difference in the number of contacts across age groups. **Panel B.** Distribution of contact duration across age groups. **Panel C.** Distribution of influenza test-negative and test-positive participants across age groups. **Panel D.** Comparison of the number of contacts between influenza test-negative and test-positive participants according to age group. **Panel E.** Comparison of contact duration between influenza test-negative and test-positive participants according to age group.

## DISCUSSION

We used a test-negative design to estimate influenza VE among participants who presented with ILI at hospitals in Ili Kazakh Autonomous Prefecture, Xinjiang during the 2024–2025 influenza season. Such a test-negative design is commonly used to evaluate VE in real-world settings because it can help reduce bias associated with differences in healthcare-seeking behaviour [39,40]. We estimated an overall VE of 42.3% (95% CrI = 11.1–62.5), indicating moderate protection against circulating strains, which is consistent with the findings from previous research [41–44]. Because Ili Kazakh Autonomous Prefecture experiences frequent tourism activities and substantial population movement, the assessment framework and VE estimates derived from this study may serve as a reference for formulating vaccination strategies in tourism-oriented regions with similarly high population mobility.

We found that subtype-specific VE was highest against influenza A(H1N1), at 45.1% (95% CrI = 13.8–65.0). This estimate is consistent with findings from our previous research and a similar study conducted in Chongqing [45,46]. The estimated VE against influenza B/Victoria of 38.3% (95% CrI = −15.7, 67.1) is lower compared to the estimates reported in studies from other regions [47–49] and our previous study in Ili, conducted in 2023–2024, where we observed a VE of 51.2% (95% CrI = 28.7–83.0) [32]. The small number of influenza B cases in our analysis (n = 20), however, may have reduced the precision of the estimate, while limited seasonal circulation may have reduced the representativeness of the cases. A potential antigenic mismatch between the circulating and vaccine strains may have also weakened vaccine-induced cross-neutralisation and contributed to the lower estimate.

We observed distinct age-stratified VE patterns, where participants aged 6 months to 6 years had the lowest VE estimate, at 37.5% (95% CrI = −0.5, 61.1), while those aged 60 years or older had the highest adjusted VE estimate, at 42.1% (95% CrI = 7.8–63.7). This age-specific pattern differs from findings reported in several international studies. Studies from the USA and UK reported lower or statistically nonsignificant VE among older adults [43,50], whereas studies from Germany and the USA reported greater protection in younger populations [51,52]. Differences in healthcare-seeking behaviour may explain these findings.

Children are more likely to seek medical care even for mild symptoms, which may produce an excess of non-influenza cases among the test-negative controls and may dilute the VE estimate. Older adults usually seek medical care only when they experience more severe illness [53,54]. This behaviour may make test-negative controls more comparable to influenza cases in healthcare-seeking and disease severity, potentially reducing this source of selection bias; a higher case proportion alone, however, does not ensure a more accurate VE estimate. Additionally, older adults may also receive additional protection through immunological memory [55]. However, we could not distinguish the relative contributions of healthcare-seeking bias and immunological mechanisms. Future research should validate these findings by using community-based cohorts or active surveillance data.

Influenza surveillance is critical for understanding viral epidemiology and determining the composition of seasonal vaccines [56]. For this reason, we used molecular evolutionary analysis to obtain additional virological context for our findings. We sequenced the HA and NA genes from six influenza-positive specimens as part of influenza surveillance and conducted a phylogenetic analysis with 170 representative GISAID sequences [57,58]. The circulating A(H1N1) strains clustered closely with the vaccine reference strain A/California/07/2009. GISAID lists this isolate as type ‘A/H1N1’ and lineage ‘pdm09’ (EPI_ISL_203615). We identified four nonsynonymous HA mutations, but only Q170K mapped to one of the known major antigenic sites (Sa, Sb, Ca, and Cb) [59]. None of the identified NA mutations affected the conserved enzymatic active site. The high conservation in key antigenic regions corresponded with the observed VE of 45.1% against A(H1N1) and was consistent with reports of a good vaccine–strain match [60]. Nonetheless, ongoing viral evolution remains a concern because enhanced transmissibility or immune escape may undermine vaccine-induced immunity. These risks support the need for continued molecular monitoring [61].

We also examined social contact patterns among outpatients to understand their role in influenza transmission. Social contact studies commonly use paper diaries, online surveys, and wearable proximity-sensing technologies to record contact duration, frequency, and context of contacts, including their time and location – data which researchers use to construct contact matrices for modelling mixing patterns [62,63]. Most studies have examined the determinants and seasonal variations in social mixing, with few comparing contact intensity according to laboratory-confirmed infection status within healthcare-seeking populations [64]. Such comparisons may provide evidence about the behavioural characteristics of infected individuals and their potential role in transmission chains, with important implications for public health. We addressed this gap by comparing social contact patterns among outpatients with different influenza test results and analysing age-specific contact intensity according to infection status. Influenza-positive participants tended to report more frequent and longer contact durations than influenza-negative participants; this pattern was observed across the overall outpatient population and was most evident among children aged 0–10 years. These findings suggest that contact behaviour may be associated with influenza test status among outpatients with ILI.

We conducted age-stratified analyses among outpatients and found that influenza-positive children aged 0–5 years had the highest contact intensity. This finding is consistent with those of community-based studies in Suzhou, China, Norway, France, and other countries [65–67]. The observed pattern paralleled the proposed role of children as primary drivers of transmission in community settings. Children’s immature immune systems may result in higher viral loads and longer periods of viral shedding after infection. They also frequently participate in social activities in daycare centres, schools, and family settings and may have less developed hygiene practices, including cough etiquette and hand cleaning. These factors may facilitate influenza transmission during close contact.

Participants aged over than 65 years or older had higher contact intensity than younger adults, regardless of their influenza test status. This finding is consistent with that of a recent study conducted in Korea [24]. Older adults in our outpatient population may participate more frequently in group social activities at community centres and senior universities or through square dancing. They may also care for grandchildren and consequently have multiple daily close contacts with children at home and in public places each day. In addition, their daily routines may also depend heavily on densely populated areas because online alternatives are unavailable.

We constructed contact matrices using data collected through influenza surveillance to characterise contact patterns among outpatients. We observed strong assortative mixing, which was most pronounced among school-aged children and least evident among older adults [27]. We also observed intergenerational mixing within households [68]. While these patterns can help characterise contacts among symptomatic individuals, the design of our study limits us from ascertaining their generalisability to the community at large. Despite this, we suggest a potential dual strategy that could encourage school-aged children to share hygiene practices within their households and provide targeted health campaigns for older adults in community centres, which may help reduce influenza transmission across age groups.

Our study has several limitations. First, we collected epidemiological data and exposure data using questionnaires, which required participants to self-report information. We did not formally assess the questionnaire’s reliability or validity, and the self-reported data may be affected by recall bias and underreporting. Questionnaire data collection may also be affected by non-response bias, meaning that respondents and nonrespondents may have differed in their baseline characteristics, which could produce baseline imbalances.

Second, we used the test-negative design primarily to evaluate VE, while the contact analyses were observational and intended to identify associations rather than establish causal relationships. We, therefore, interpreted the observed differences in contact frequency as an association between contact behaviour and infection risk. Furthermore, the inclusion of vaccinated participants introduced the possibility that vaccination status modified the association between contact frequency and infection risk, as all three factors are interrelated. Consequently, their effects may not have been fully disentangled in our models. We also adjusted for the available covariates of age, sex, and vaccination status, but unmeasured factors, including occupation, household size, community contact intensity, healthcare-seeking behaviour, and chronic diseases, may have caused residual confounding. The sensitivity analysis produced similar contact-related findings, suggesting that the inclusion of vaccinated participants did not substantially alter the results (Text S5 in the **Online Supplementary Document**). Yet we did not jointly model contact frequency, vaccination status, and infection risk, fit an interaction model, or stratify the analyses by contact level or viral subtype because of the exploratory nature of the analyses and the limited sample size. We thus reported the three components in parallel. Future research should model their interrelationships explicitly and use prospective cohorts or contact-network transmission models to assess potential causal relationships.

Third, we derived the social contact patterns from a hospital-based sample of participants with ILI who sought healthcare. We therefore cannot interpret these contact matrices as representative of community-wide mixing patterns because participants’ healthcare-seeking behaviour and symptomatic status may have influenced the observed patterns. Fourth, we conducted this study only in the Ili Kazakh Autonomous Prefecture, so our findings may not fully represent the diverse conditions across Xinjiang. Lastly, we sequenced only a small sample of six influenza-positive specimens; our sequencing results should, therefore, be considered as exploratory, meaning that generalisable conclusions cannot be drawn about the characteristics or evolution of circulating strains.

## CONCLUSIONS

We found through an integrated analysis that influenza vaccination provided moderate real-world protection against medically attended influenza during the 2024–2025 influenza season in Ili, a border region of Xinjiang. Higher frequency of self-reported contacts and longer contact duration were associated with influenza test positivity, especially among children and older adults. Local authorities could consider prioritising vaccination for these age groups, strengthening non-pharmaceutical interventions, including ventilation, mask use, and reducing gatherings in schools, nursing homes, and households. Linking vaccination records with electronic health records could also help strengthen VE surveillance. Public health authorities in border areas with limited resources could consider combining vaccination with targeted behavioural measures to reduce outpatient influenza burden. School-based campaigns for children and community outreach for older adults may provide complementary layers of protection. Future studies should use community-based cohorts, measure contacts objectively using wearable devices, conduct viral sequencing on larger samples, and explicitly assess whether contact levels modify VE.

## Additional material


Online Supplementary Document
