# Supplementary material for: Influenza vaccine protection and contact patterns among outpatients with influenza-like illness during the 2024–2025 season in China: a test-negative case-control study
Source: J Glob Health. 2026 Oct 9;16:04253. doi: 10.7189/jogh.16.04253 (PMC13650974; doi:10.7189/jogh.16.04253)
Supplement: Supplementary file 1 [file jogh-16-04253-s001.zip › jogh-16-04253-s001.pdf]

Supplementary materials

Table of Contents

S1. Data and variables 2
S1.1 Patient recruitment 2
S1.2 Questionnaire design and implementation 2
S1.2.1 Questionnaire content 2
S1.2.2 Questionnaire administration 6
S1.3 Detection of influenza viruses 6
S1.3.1 Specimen collection and preservation 6
S1.3.2 Nucleic acid extraction and influenza virus identification 6
S1.3.3 Virus isolation and culture 6
S1.3.4 Targeted sequencing and genetic analysis 7
S2. Calculation of vaccine effectiveness (VE) 8
S3. Phylogenetic analysis process 9
S3.1 Sequence collection and alignment 9
S3.2 Phylogenetic analyses 9
S3.3 Differential site analysis between major clusters 10
S3.4 Phylogenetic analysis of the H1N1 influenza virus NA gene 13
S3.5 In silico prediction of vaccine effectiveness based on viral gene sequences 13
S4. Social contact analysis process 15
S4.1 Statistical analysis 15
S4.1.1 Individual-level contact patterns 15
S4.1.2. Contact matrices 16
S4.2 Factors influencing social contact patterns during the 2024/25 influenza season 17
S4.3 Contact patterns and demographics by influenza status 20
S4.4 Associations between contact type, setting, age, and influenza status 22
S4.4.1. Analysis of age-specific contact patterns by setting 22
S4.4.2. Analysis of the influence of contact setting, frequency, and duration on contact type 23
S4.4.3 Association of contact frequency and setting with influenza 25
S4.5 Age-specific effects of contact settings on influenza and their distributions 27
S4.6 Association analysis of influenza and contact patterns across demographic subgroups 32
S4.6.1. Analysis of the impact of sex stratification on contact number and duration 32
S4.6.2 Analysis of the impact of household size on contact number and duration 36
S4.6.3. Analysis of the impact of contact settings on contact number and duration 42
S5. Sensitivity analysis 49
S5.1 Vaccine effectiveness 49
S5.1.1 Sensitivity analysis expanded to include children aged under 6 months 49
S5.1.2 Sensitivity analysis using age and BMI as continuous variables 50
S5.1.3 Sensitivity analysis incorporating the calendar date 51
S5.2 Contact patterns 52
Supplementary references 55

S1. Data and variables

S1.1 Patient recruitment

This was a test-negative study based on the surveillance of influenza-like illness (ILI) by sentinel hospitals. Unlike many previous studies that used the WHO definition of ILI ("*an acute respiratory infection with fever (≥38°C) and cough, with an onset within the last 10 days*" [1]), we adopted the following definition from the Chinese CDC: "*fever (body temperature ≥38°C) accompanied by cough or sore throat*" [2], as our study was conducted in mainland China. The subject group consisted of ILI patients who tested positive in the laboratory, while the control group consisted of patients who tested negative.

During the 2024–2025 influenza season, three sentinel hospitals in Ili Prefecture were integrated into the influenza virology surveillance system of China's Xinjiang Uygur Autonomous Region. Using a convenience sampling method, trained clinicians collected information and throat swab specimens weekly from ILI patients who presented at the outpatient departments of each hospital (including internal medicine, adult emergency, fever clinic, or paediatric emergency departments). On average, 20 ILI specimens (minimum 10 and maximum 40 specimens) were collected per hospital each week. The specimens were transported in viral transport medium at 4°C to collaborating laboratories within 24 hours and tested for influenza viruses via reverse real-time transcription polymerase chain reaction (rRT–PCR), in accordance with protocols recommended by the Chinese National Influenza Centre (CNIC; https://www.chinacdc.cn/). Given the characteristic winter peak of influenza activity in Xinjiang, the study period for the final analysis was defined as December 8, 2024, to March 30, 2025. All subjects included in the analysis had sought outpatient care at the sentinel hospitals and completed laboratory testing within this period.

S1.2 Questionnaire design and implementation

S1.2.1 Questionnaire content

A structured questionnaire comprising three primary sections was used for data collection. In the first section, demographic information, including sex, age, ethnicity, education level, occupation, and household size, was collected. The second section pertained to health status and behavioural characteristics and included height, weight, smoking status, comorbidities, dates of ILI onset and specimen collection, and influenza vaccination history within the preceding 24 months. The third section documented contact behaviours across different settings. The subjects were asked to recall all contacts from the day preceding the survey (i.e., the 24-hour period before the interview), including each contact's age, sex, type, duration, location, and usual frequency of interaction with that contact.

The questionnaire employs a standardized approach for age estimation. The subjects were instructed to provide their exact age only if known or readily estimable; otherwise, they selected an age range. These data were then uniformly processed via a "binned age" method, which categorized the contacts into 15 mutually exclusive and consecutive age groups, namely, 0–4, 5–9, 10–14, 15–19, 20–24, 25–29, 30–34, 35–39, 40–44, 45–49, 50–54, 55–59, 60–64, 65–69, and ≥70 years. The definitions of different contact settings are summarized in **Table S1** [3]. In addition, if an individual had contact in multiple settings, only one entry was created, and the total cumulative contact duration for that day was noted.

Table S1. Definitions of settings for the contacts of influenza-like illness subjects.

**Setting**	**Contact Definition**
Household	Contacts who live with or provide care for an influenza-like illness (ILI) subject.
School	Subjects who had contact with an ILI subject in an educational system, such as kindergartens, primary and secondary schools, or universities.
Workplace	Individuals who work or perform official duties in the same premises as ILI patients include the following two categories.<br>1) Colleagues who share workspaces or frequently use common areas within various organizations such as companies, factories, or government departments.<br>2) Faculty and staff employed at the same educational institution (including daycare centres, primary and secondary schools, and universities), such as teachers and administrative personnel.
Leisure	Contacts of ILI subjects in public or private settings for social or recreational purposes, such as subjects in group activities or fitness classes and individuals sharing a meal in restaurants.
Others	Contacts of ILI subjects encountered in scenarios not covered above or that are difficult to define, including situations such as neighbourhood encounters in residential common areas such as stairwells and elevators, brief shared travel on public transport or in nonleisure carpools, and casual contact in crowded places such as streets or shopping malls.

Contact survey (Chinese version in use)

年龄	性别		是否有身体接触		您通常多久与此人联系一次?					您在哪里接触过此人?<br>（可多选）					在整个接触过程中与此人相处的总时长				
（区间范围）	男	女	是	否	每天或<br>几乎每天	大约每周一两次	大约每月一两次	每月<br>少于一次	从未<br>见过面	家庭	学校	工作场所	休闲场所	其他	少于<br>5 分钟	5-15 分钟	15 分钟 - 1 小时	1-4 小时	4 小时以上
□□_□□	□	□	□	□	□	□	□	□	□	□	□	□	□	□	□	□	□	□	□
□□_□□	□	□	□	□	□	□	□	□	□	□	□	□	□	□	□	□	□	□	□
□□_□□	□	□	□	□	□	□	□	□	□	□	□	□	□	□	□	□	□	□	□
□□_□□	□	□	□	□	□	□	□	□	□	□	□	□	□	□	□	□	□	□	□
□□_□□	□	□	□	□	□	□	□	□	□	□	□	□	□	□	□	□	□	□	□
□□_□□	□	□	□	□	□	□	□	□	□	□	□	□	□	□	□	□	□	□	□
□□_□□	□	□	□	□	□	□	□	□	□	□	□	□	□	□	□	□	□	□	□
□□_□□	□	□	□	□	□	□	□	□	□	□	□	□	□	□	□	□	□	□	□
□□_□□	□	□	□	□	□	□	□	□	□	□	□	□	□	□	□	□	□	□	□
□□_□□	□	□	□	□	□	□	□	□	□	□	□	□	□	□	□	□	□	□	□
□□_□□	□	□	□	□	□	□	□	□	□	□	□	□	□	□	□	□	□	□	□
□□_□□	□	□	□	□	□	□	□	□	□	□	□	□	□	□	□	□	□	□	□

您是否填写完所有接触者：

□ 是　　□ 否

Contact survey (translated English version)

**Age**	**Sex**		**Did you touch his or her skin?**		**How often do you have contact with this person generally?**					**Where did you have contact with this person? (tick all that apply on your assigned day)**					**Total time spent with this person during whole day**				
**(or range)**	**F**	**M**	**Y**	**N**	**Daily or almost daily**	**About once or twice a week**	**About once or twice a month**	**Less than once a month**	**Never met before**	**Household**	**School**	**Workplace**	**Leisure**	**Others**	**Under 5 mins**	**5-15 mins**	**15 mins - 1 hr**	**1-4 hrs**	**More than 4 hrs**
☐☐_☐☐	☐	☐	☐	☐	☐	☐	☐	☐	☐	☐	☐	☐	☐	☐	☐	☐	☐	☐	☐
☐☐_☐☐	☐	☐	☐	☐	☐	☐	☐	☐	☐	☐	☐	☐	☐	☐	☐	☐	☐	☐	☐
☐☐_☐☐	☐	☐	☐	☐	☐	☐	☐	☐	☐	☐	☐	☐	☐	☐	☐	☐	☐	☐	☐
☐☐_☐☐	☐	☐	☐	☐	☐	☐	☐	☐	☐	☐	☐	☐	☐	☐	☐	☐	☐	☐	☐
☐☐_☐☐	☐	☐	☐	☐	☐	☐	☐	☐	☐	☐	☐	☐	☐	☐	☐	☐	☐	☐	☐
☐☐_☐☐	☐	☐	☐	☐	☐	☐	☐	☐	☐	☐	☐	☐	☐	☐	☐	☐	☐	☐	☐
☐☐_☐☐	☐	☐	☐	☐	☐	☐	☐	☐	☐	☐	☐	☐	☐	☐	☐	☐	☐	☐	☐
☐☐_☐☐	☐	☐	☐	☐	☐	☐	☐	☐	☐	☐	☐	☐	☐	☐	☐	☐	☐	☐	☐
☐☐_☐☐	☐	☐	☐	☐	☐	☐	☐	☐	☐	☐	☐	☐	☐	☐	☐	☐	☐	☐	☐
☐☐_☐☐	☐	☐	☐	☐	☐	☐	☐	☐	☐	☐	☐	☐	☐	☐	☐	☐	☐	☐	☐
☐☐_☐☐	☐	☐	☐	☐	☐	☐	☐	☐	☐	☐	☐	☐	☐	☐	☐	☐	☐	☐	☐
☐☐_☐☐	☐	☐	☐	☐	☐	☐	☐	☐	☐	☐	☐	☐	☐	☐	☐	☐	☐	☐	☐

Have you completed this questionnaire?

☐ Yes ☐ No

S1.2.2 Questionnaire administration

In this study, the questionnaire was administered via two distinct modalities [4]. For respondents who were readily available and able to cooperate onsite at sentinel hospitals, face-to-face interviews were conducted by trained healthcare professionals or public health practitioners. For those unable to participate onsite, questionnaire data were collected via telephone interviews following their clinical visit.

The mode of information provision was tailored to the subject's age. Accompanying parents provided information for children aged 0 to 10 years; self-reported data were obtained from subjects aged 11 to 17 years after informed consent was obtained from their parents; subjects aged 18 to 64 years provided information independently; and for subjects aged 65 years and above, information was provided with the assistance of accompanying family members (if necessary). For all groups, the provided information was verified and systematically documented by staff to ensure data accuracy and completeness.

S1.3 Detection of influenza viruses

S1.3.1 Specimen collection and preservation

In this study, nasopharyngeal swab specimens were collected from enrolled ILI subjects in strict accordance with the China National Influenza Surveillance Program (2017 edition) [2]. The target population was ILI patients within three days of symptom onset. Immediately after collection, the specimens were placed in tubes containing 3–4 mL of viral transport medium, stored at 2–8°C, and transported to collaborating influenza surveillance network laboratories within 48 hours. Specimens that were not delivered within this time frame were stored at -70°C or below, and precautions taken to avoid repeated freeze–thaw cycles.

S1.3.2 Nucleic acid extraction and influenza virus identification

Upon arrival at the laboratory, the specimens were processed according to standard procedures. Viral nucleic acids were extracted via a magnetic bead-based kit designed for the extraction, enrichment, and purification of DNA or RNA from clinical samples. Under specific high-salt conditions, nucleic acids released from lysed cells or viruses were adsorbed onto magnetic beads coated with polyhydroxy groups. After the impurities were removed by washing, the purified nucleic acids were eluted in a suitable buffer for downstream applications. Subsequently, influenza virus detection and typing were performed with the Zhuocheng Hui Sheng Influenza A/B Duplex Real-time RT–PCR Detection Kit (website link: https://www.x-abt.com/portal/article/index/id/80/cid/31.html, which is in Chinese). This kit employs specific primers and fluorescent probes for the qualitative detection of influenza A and B virus nucleic acids from throat swabs, nasal swabs, and sputum extracts. Each reaction includes an internal control to monitor the entire process from sampling to extraction, thereby reducing the risk of false-negative results. Testing was conducted on validated real-time PCR systems, including an ABI 7500, a Bio-Rad CFX96, a Roche LightCycler 480II, and a Shanghai Hongshi SLAN-96P system, all of which support multichannel fluorescence detection for FAM, VIC/HEX, and CY5 signals.

S1.3.3 Virus isolation and culture

To further analyse viral biological characteristics, a subset of nucleic acid-positive specimens was subjected to virus isolation. Sample aliquots were inoculated into Madin–Darby canine kidney (MDCK) cells, specific pathogen-free (SPF) chicken embryos, or both. Viral growth was assessed

through a haemagglutination assay, and isolates showing haemagglutination activity were identified as influenza virus strains.

S1.3.4 Targeted sequencing and genetic analysis

Sequencing libraries were constructed via next-generation sequencing platforms (e.g., Illumina). Specifically, cDNA was first synthesized via reverse transcription. The target viral genome was then enriched through multiplex PCR using influenza virus-specific primers. The resulting amplicons were subsequently purified, and sequencing libraries were constructed via kits such as the Nextera XT kit. Finally, the libraries were sequenced on high-throughput platforms, including MiSeq or NextSeq.

For in-depth genetic characterization, whole-genome sequencing was performed on selected influenza-positive samples and isolated virus strains. An amplicon-based targeted sequencing approach was used to sequence all eight segments of the influenza virus genome: haemagglutinin (HA), neuraminidase (NA), polymerase basic 1 (PB1), polymerase basic 2 (PB2), polymerase acidic (PA), nucleoprotein (NP), matrix protein (M) and nonstructural protein (NS).

Sequencing was performed on the Illumina platform. Target fragments were first enriched via multiplex PCR amplification with influenza-specific primers. The amplified products were then purified, and sequencing libraries were constructed with the Nextera XT Library Prep Kit. Finally, high-throughput sequencing was conducted with the MiniSeqDx-CN system.

Raw sequencing data were processed and assembled with WeFuture Influenza Analysis Software. Single-nucleotide polymorphism (SNP) analysis was then conducted with SAMtools [5] and GATK [6]. Sequence alignment and phylogenetic analysis were performed in MEGA [7] software.

S2. Calculation of vaccine effectiveness (VE)

This observational study employed a test-negative design (TND) to estimate annual influenza vaccine effectiveness (VE). Cases were defined as patients who tested positive for influenza, and controls were defined as those who tested negative (i.e., those with noninfluenza respiratory illnesses) [8].

A Bayesian logistic regression model was used to assess the association between vaccination status and the risk of influenza infection, from which VE was calculated. The model was fitted with the "*bayesglm*()" function from the "*arm*" package in **R** [9].

The dependent variable in the model was the influenza virus test result (a binary variable: 1 = positive, 0 = negative). The primary independent variable was the vaccination status (1 = vaccinated, 0 = unvaccinated). The model was adjusted for potential confounders, including sex, the number of days from symptom onset to specimen collection, age group, and BMI group.

All model parameters were assigned T-distributed priors. Specifically, for the vaccination status coefficient, we set an informative prior whose mean was derived from VE estimates reported in previous studies and calculated as log(1 − prior VE), thereby incorporating existing evidence into our model. For the coefficients of all the other covariates, weakly informative priors with a mean of zero were specified. The degrees of freedom for all T-distributed priors were set to infinity, making them equivalent to normal distributions.

VE and its 95% credible interval (CrI) were calculated as (1 − OR) × 100%, where the odds ratio (OR) represents the exponentiated regression coefficient for the vaccination status, obtained from the posterior distribution of the model.

S3. Phylogenetic analysis process

S3.1 Sequence collection and alignment

In this study, six sample sequences from collected viral isolates were used, supplemented by 170 reference sequences systematically retrieved from the GISAID EpiFlu database [10]. These reference sequences, which are complete and include both the HA and NA segments, span a 17-year period (from 2009 to 2025), with ten representative sequences selected per year.

Multiple sequence alignment was conducted via the “ClustalW” algorithm in MEGA12 [7], followed by manual refinement of the initial output. To improve the robustness of subsequent phylogenetic inference, poorly aligned terminal regions and gap-dense areas were trimmed, and only high-quality alignments were retained. This process yielded final alignments of 1,701 bp (HA gene) and 1,410 bp (NA gene) for phylogenetic analysis.

S3.2 Phylogenetic analyses

To further investigate viral evolutionary relationships, we conducted separate phylogenetic analyses of the HA and NA gene sequences. Phylogenetic trees were constructed via the maximum likelihood (ML) method in IQ-TREE 3 [11]. The optimal nucleotide substitution model for both trees, selected under the Bayesian information criterion (BIC), was the transitional model with empirical base frequencies and a discrete Gamma model with 4 rate categories (TIM+F+G4).

The branch supports were then evaluated via the ultrafast bootstrap method (UFBoot; 1,000 replicates) and SH-like approximate likelihood ratio tests (SH-aLRT). Branches with UFBoot support ≥95% and SH-aLRT support ≥80% were considered statistically well supported. The final phylogenetic tree was visualized and annotated via the “*ggtree*” package in **R** statistical software[12].

S3.3 Differential site analysis between major clusters

To identify amino acid variations between the two major evolutionary clusters of each gene, we conducted differential site analysis on the basis of the established phylogenetic framework. This analysis involved calculating the conserved sequences for each cluster and aligning them with the reference strain A/California/07/2009, thereby identifying stable variations at amino acid sites.

Sequence analysis revealed several nonsynonymous mutations. In the HA gene, mutations A121T, Q170K, and T217A were identified within the receptor-binding HA1 domain (a region critical for receptor binding and antigenic epitopes), which alter amino acid hydrophilicity and charge, respectively. (**Table S2**, and **Figure S1**). In the NA gene, two mutation (N184S and T248I) were located in the head domain (**Table S3**, and **Figure S2**). The N184S substitution, which replaces a polar neutral with a polar hydrophilic amino acid, could affect the hydrophilicity or glycosylation potential of the region, thereby potentially modulating NA enzyme activity or antigenic epitopes. The T248I substitution, which replaces a polar hydrophilic with a nonpolar hydrophobic amino acid, could similarly alter local hydrophilicity and potentially impact NA function.

The three-dimensional structural visualizations presented in this study were generated with PyMOL software (version 3.1) [13]. The crystal structures included the HA from a 2009 H1N1 influenza virus (PDB ID: 3LZG) (website link: https://www.rcsb.org/structure/3LZG) [14] and the NA in complex with a human antibody Fab fragment from the A/California/04/2009 (H1N1) strain (PDB ID: 6Q23) (website link: https://www.rcsb.org/structure/6Q23) [15]; both were obtained from the Protein Data Bank (PDB) [16]. Following import, the structures underwent basic preprocessing to optimize visual clarity. Nonsynonymous mutation sites, identified through prior sequence alignment analysis, were precisely mapped onto the three-dimensional structures. For visualization, protein backbones are depicted in cartoon representation to illustrate the overall fold, and the key mutation residues were located with PyMOL selection commands and are highlighted in red to accentuate their positions and facilitate structural comparison.

Table S2. Nonsynonymous mutations in the haemagglutinin (HA) segment.

**Codon position**	**Ref AA**	**Mutant AA**	**Mature position**	**Domain**	**Mutation**
137	A	T	121	HA1 Domain	A121T
186	Q	K	170	HA1 Domain	Q170K
233	T	A	217	HA1 Domain	T217A
435	V	I	419	HA2 Domain	V419I

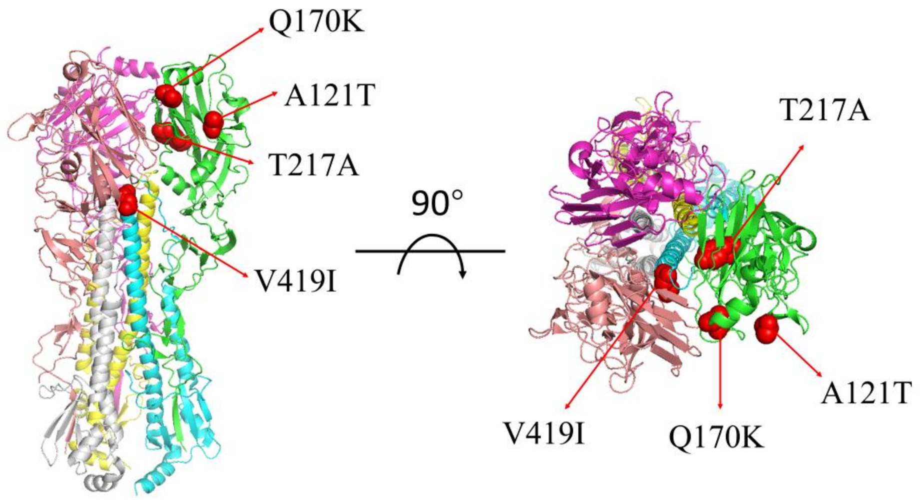

Figure S1. Crystal structure of the influenza virus haemagglutinin (HA) protein with A121T, Q170K, T217A, and V419I mutations. The structure is shown in two orthogonal views (one rotated 90° relative to the other) to display different aspects of the molecular architecture. Both views are in cartoon representation. Individual protein chains within the crystallographic asymmetric unit are coloured consistently across both panels: chain A in vivid green, chain B in cyan, chain C in hot pink, chain D in yellow, chain E in salmon, and chain F in grey. The mutation sites are highlighted with red spheres in both views.

Table S3. Nonsynonymous mutations in the neuraminidase (NA) segment.

AA position	Ref AA	Mutant AA	Mature position	Domain	Mutation
200	N	S	184	Head Domain	N184S
264	T	I	248	Head Domain	T248I

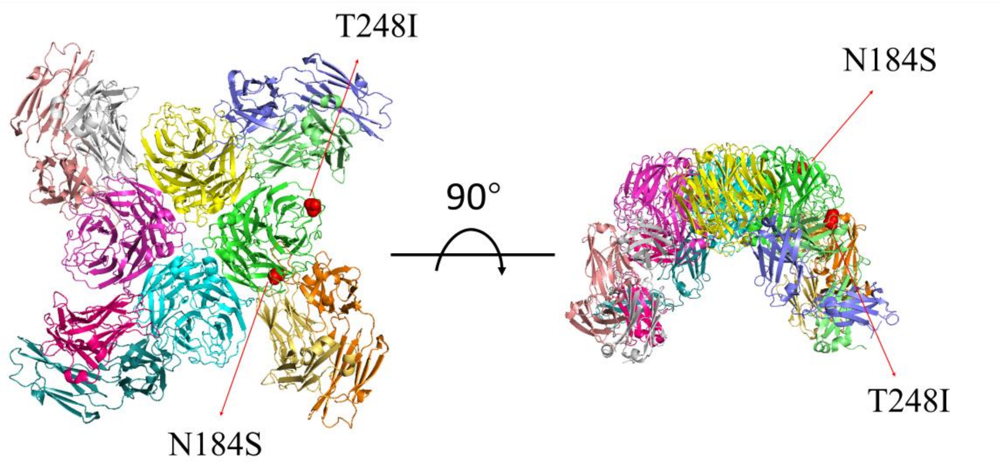

Figure S2. Crystal structure of the influenza virus neuraminidase (NA) protein with N184S and T248I mutations. The structure is shown in two orthogonal views (one rotated 90° relative to the other) to depict different aspects of the molecular architecture. Individual protein chains within the crystallographic asymmetric unit are coloured as follows: chain A in vivid green, chain B in cyan, chain C in hot pink, chain D in yellow, chain E in salmon, chain F in grey, chain G in purple, chain H in orange, chain I in light green, chain J in turquoise, chain K in deep pink, and chain L in light brown. The mutation sites are highlighted with red spheres.

S3.4 Phylogenetic analysis of the H1N1 influenza virus NA gene

For viral sequencing, we performed HA and NA gene sequencing on six influenza-positive samples. Phylogenetic analysis was conducted by integrating these sequences with 170 representative subtype-specific strain sequences retrieved from the GISAID database to explain the molecular evolutionary relationships of the currently circulating strains. The results revealed that the HA and NA genes of the circulating H1N1 strains clustered with andwere closely related to those of the vaccine reference strain A/California/07/2009 (H1N1)pdm09. Furthermore, substitution site analysis was performed on a selected clade comprising 51 sequences, which revealed two nonsynonymous mutations, N184S and T248I , in the NA gene.

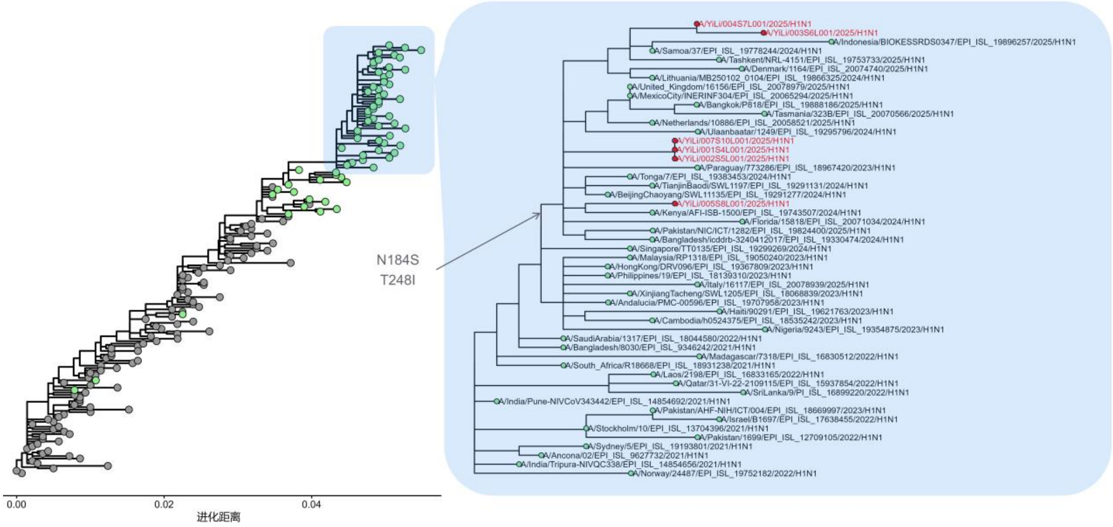

Figure S3. Phylogenetic tree of the neuraminidase (NA) gene from the H1N1 influenza virus. The sample sequences are highlighted in red, of which five were collected in week 51 of 2024 and one in week 52. The reference sequences are shown in black. The results of the analysis revealed that the NA gene maintains overall genetic stability despite harbouring some degree of genetic diversity.

S3.5 In silico prediction of vaccine effectiveness based on viral gene sequences

To provide a complementary genomic assessment of vaccine effectiveness (VE), we applied the sequence-based model established by Cao et al. [17] for *in silico* prediction. This model enables real-time estimation of influenza VE directly from viral genetic sequences by quantifying the number of nonsynonymous mutations in the HA gene relative to the vaccine strain, a metric referred to as "effective mutation distance" (EMD) byin Cao et al. [17].

The HA sequences of the six H1N1 influenza virus strains described in Section S3.1 were aligned with the World Health Organization (WHO)-recommended Northern Hemisphere vaccine strain for the 2023/24 season, A/Victoria/4897/2022 (H1N1)pdm09-like virus [18], and a total of 11 nonsynonymous mutations were identified across the six isolates compared to the vaccine strain. The total number of nonsynonymous mutations (n = 11) was input into the linear regression model provided by Cao et al. [17] for the H1N1pdm09 subtype,

VE (%) = 70.7 − 2.28 × (number of nonsynonymous HA mutations).

The predicted VE of 45.62% is close to the estimated VE of 42.3% in our study, thereby providing independent, genomic-level validation for our main findings (**Figure S4**).

A

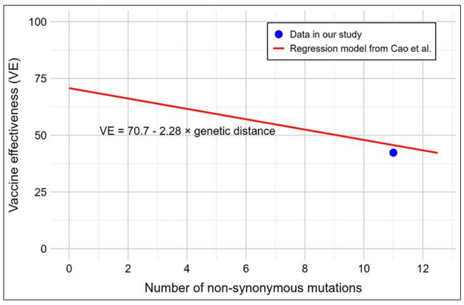

B

influenza A virus stains	codon position on HA segment										
	120	137	142	169	216	223	260	277	294	356	451
A/Victoria/4897/2022/H1N1 (WHO recommended vaccine strain)	T	S	R	K	A	R	E	A	N	D	H
A/YiLi/001S4L001/2025/H1N1	A	P	K	Q	T	Q	D	T	K	E	N
A/YiLi/002S5L001/2025/H1N1	A	P	K	Q	T	Q	D	T	K	E	N
A/YiLi/003S6L001/2025/H1N1	A	P	K	Q	T	Q	D	T	N	E	N
A/YiLi/004S7L001/2025/H1N1	A	P	K	Q	T	Q	D	T	N	E	N
A/YiLi/005S8L001/2025/H1N1	A	S	K	Q	T	Q	D	T	N	E	N
A/YiLi/007S10L001/2025/H1N1	A	P	K	Q	T	Q	D	T	K	E	N

Figure S4. Relationship between viral HA sequence variation and vaccine effectiveness (VE). Panel A showed the prediction of vaccine effectiveness based on the basis of the number of nonsynonymous mutation sites in the HA gene. The red dashed line represents the regression model by Cao et al. (2021)[17] for H1N1pdm09. The blue dot indicates the predicted VE (45.62%) derived from the 11 site variations identified in the HA sequences of the six viral strains in this study. Panel B showed the amino acid variation at key sites in the HA protein of the six H1N1 viruses in this study relative to the vaccine strain A/Victoria/4897/2022/H1N1.

S4. Social contact analysis process

S4.1 Statistical analysis

S4.1.1 Individual-level contact patterns

In this study, mathematical modelling was employed to convert qualitative responses from the social contact survey into quantitative epidemiological metrics [19].

(1) Quantification of contact frequency and duration

We used a right-truncated, weighted calculation method to concurrently quantify the contact frequency and duration. For frequency, each qualitative response was converted into a daily contact rate f_C. The high-frequency option ("daily or almost daily") was quantified with the arithmetic mean. The medium- and low-frequency options ("approximately 1 to 2 times per week", "approximately 1 to 2 times per month", "less than once per month", and "no contact") were converted into daily rates according to their respective time scales. For duration, categorical responses ("less than 5 minutes", "5 to 15 minutes", "15 minutes to 1 hour", "1 to 4 hours" and "more than 4 hours") were converted into hourly values d_t via the interval midpoint method. Right truncation was applied to the highest duration category, in which "more than 4 hours" was capped at 4 hours.

On the basis of the quantified values described above, two metrics were computed for each individual *i*. If individual *i* reported J_i contacts, his or her average daily number of contacts, N_i, and the average daily contact person-hours, H_i, were calculated as follows:

$$N_i = \sum_{j=1}^{J_i} f_{ij}, \text{ and } H_i = \sum_{j=1}^{J_i} \left(f_{ij} \times d_{ij}\right),$$

where f_{ij} and d_{ij} represent the quantified values of frequency and duration for the *j*-th contact, respectively.

(2) Statistical modelling and Bayesian estimation

Since the distributions of the individual number of contacts N_i and contact person-hours H_i typically exhibit overdispersion, a negative binomial distribution was adopted for modelling; its probability mass function is given by

$$P(X=n \mid \mu,\theta) = \frac{\Gamma(n+\theta)}{\Gamma(\theta) n!} \left(\frac{\mu}{\mu+\theta}\right)^n \left(\frac{\theta}{\mu+\theta}\right)^\theta,$$

where μ represents the mean contact intensity and θ represents the quantified heterogeneity in contact patterns among individuals. Within the Bayesian framework, weakly informative prior distributions were specified for both parameters.

The posterior distributions were sampled with a Markov chain Monte Carlo (MCMC) algorithm, running 15,000 iterations. The first 5,000 iterations were discarded as burn-in samples, and only every 5th iteration was retained to reduce autocorrelation. From the resulting posterior samples, we computed the mean, standard deviation, and 95% credible interval for the parameters.

(3) Robustness assessment and between-group difference analysis

To assess the robustness of the estimates, we also fitted a gamma distribution to the data using maximum likelihood estimation. The probability density function of the gamma distribution is as follows

$$f(x \mid \alpha,\beta) = \frac{\beta^\alpha}{\Gamma(\alpha)} x^{\alpha-1} e^{-\beta x}.$$

The shape parameter α and rate parameter β were estimated by maximizing the likelihood function $L(\alpha,\beta)=\prod_{i=1}^{n} f(x_i \mid \alpha,\beta)$. Credible intervals were constructed by sampling from the asymptotic, normal distributions of these parameter estimators.

Furthermore, to quantify the disparity in contact behaviours between the test-positive and test-negative populations, we fitted the above distributions to each group separately. The distributional difference and its uncertainty were evaluated by comparing the posterior distributions of the parameters, for example, by examining the posterior distribution of the difference $\mu_{pos}-\mu_{neg}$. The results of this method are presented in the **Supplementary Materials**, **S4.5**.

S4.1.2. Contact matrices

The dataset was stratified into fifteen five-year age groups, ranging from 0–4 years to 70 years and above. For each pair of age groups i and j (i, j = 1, …, 15), an age-specific contact matrix M was constructed, where each element m_{ij} represents the contact intensity from age group i to age group j, based on either the daily number of contacts or the contact person-hours[20].

(1) Matrix interpolation and smoothing

To derive contact patterns at a finer age resolution, the 15×15 contact matrix was first disaggregated into a one-year resolution matrix M^{interp} via a nearest-neighbour interpolation algorithm. For any given target age x and y (x,y=0,1,…,70), the interpolated value is given by

$$m_{xy}^{\text{interp}}=m_{\lfloor x/5\rfloor+1,\lfloor y/5\rfloor+1}.$$

Here, m represents the number of contacts or contact duration between specific ages. m_{xy}^{interp} denotes the interpolated contact matrix, which shows the number of contacts or contact duration reported by participants aged x with individuals aged y. $\lfloor x\rfloor$ denotes the floor function, which maps a real number x to the greatest integer less than or equal to x.

The interpolated matrix was subsequently smoothed via a two-dimensional Gaussian kernel as follows:

$$m_{xy}^{\text{smooth}}=\frac{1}{W}\sum_{u=-k}^{k}\sum_{v=-k}^{k} m_{x+u,y+v}^{\text{interp}}\, G(u,v;\sigma),$$

where m_{xy}^{smooth} represents the smoothed contact matrix, showing the number of contacts or contact duration reported by participants aged x with individuals aged y. $G(u,v;\sigma)=\exp\left(-\frac{u^2+v^2}{2\sigma^2}\right)$ denotes the Gaussian kernel, $W=\sum_{u=-k}^{k}\sum_{v=-k}^{k} G\,(u,v;\sigma)$ is a normalization constant (ensuring that the sum of the kernel weights is 1), k is the kernel radius, and σ is the bandwidth parameter controlling the degree of smoothing.

(2) Age-standardized processing

Direct age standardization was performed to compare contact differences between influenza test-positive and test-negative subjects with the 2020 Chinese census population used as the standard

[21]. Let p_a be the proportion of the standard population in age group a, and let $d_a = m_a^{\text{smooth,positive}} - m_a^{\text{smooth,negative}}$ be the observed contact difference for that age. The age-standardized mean difference (ASMD) is an index of the overall difference and is calculated as follows:

$$\text{ASMD} = \sum_{a=0}^{n} d_a p_a.$$

(3) Age stratification and regression analysis

The smoothed contact matrix was partitioned into four clinically meaningful age strata $G_1=[0,5]$, $G_2=[6,15]$, and $G_3=[16,65]$, and $G_4=[66,\infty)$. For each stratum g, a linear regression model was formulated as follows:

$$E[y_g]=\beta_{0,g}+\beta_{1,g}I_{\text{positive}},$$

where y_g represents the aggregate contact intensity within age stratum g. I_{positive} is a binary indicator variable for infection status (1 = test-positive, 0 = test-negative). $\beta_{1,g}$ is the regression coefficient quantifying the contact difference of the positive group relative to the negative group in stratum g. The estimated regression coefficients $\widehat{\beta}_{1,g}$ for each age stratum represent the proportional relationship in contact behaviour between test-positive and test-negative individuals. The results of this method are presented in the **Supplementary Materials**, **S4.6**.

S4.2 Factors influencing social contact patterns during the 2024/25 influenza season

We analysed the trends in reported contact levels across different epidemiological weeks during the 2024/25 influenza season. As shown in **Figure S5,** the contact levels exhibited temporal fluctuations throughout the season. During the 52-nd and 53-rd weeks, social contact levels were relatively high. In contrast, the contact levels recorded during school holidays and the Chinese Lunar New Year (also known as the Spring Festival) were significantly lower. Following these holidays, contact levels gradually increased, reflecting the notable impact of the holiday periods.

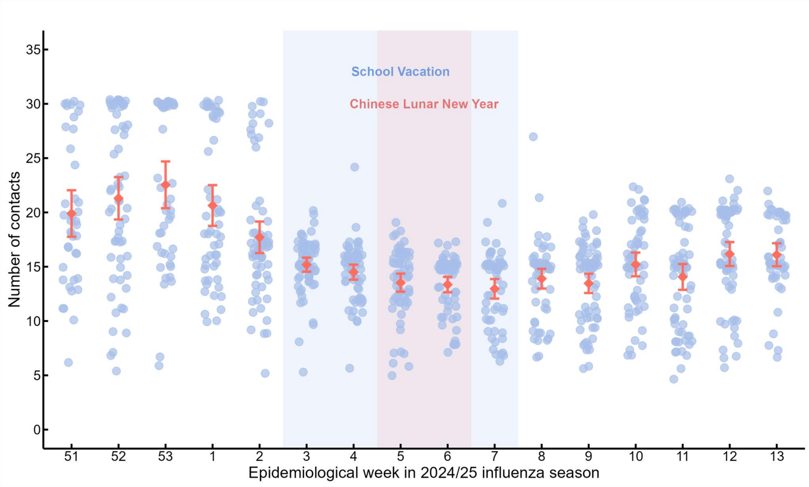

Figure S5. Distribution of contact numbers across epidemiological weeks in the 2024/25 influenza

season. The blue shading represents school vacation from January 20 to February 16, 2025. The red shading represents the Chinese Lunar New Year vacation from January 27 to February 9, 2025.

After stratification by sex, we observed consistent temporal fluctuations in both the male and female groups. Although the peak contact level was initially slightly higher in males, the levels gradually aligned. Furthermore, compared with those found for the male group, the distributions found for the female group exhibited less variability both within and between groups (**Figure S6**).

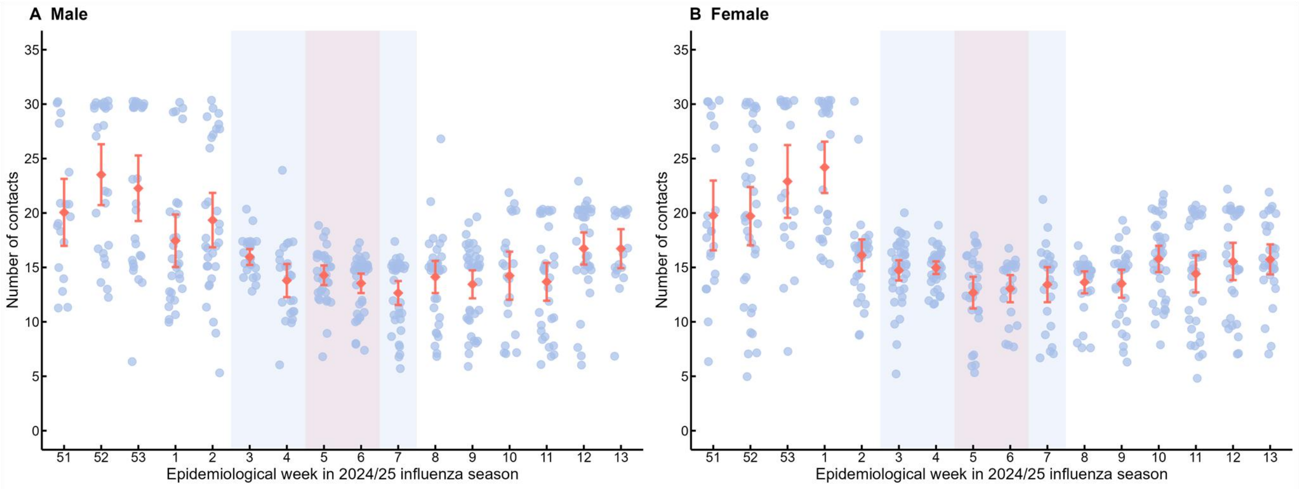

Figure S6. Distribution of contact numbers across epidemiological weeks in the 2024/25 influenza season, stratified by **sex**. Panels A and B showed the distributions for males and females, respectively. All panels were presented in the same style as in Figure S5.

The study population was also stratified into three age groups: 0 to 17, 18 to 59, and ≥60 years. The analysis revealed that the reported contact levels among those aged 0 to 17 and 18 to 59 years exhibited clear temporal fluctuations. Notably, the 0 to 17 age group showed a pronounced decline in reported contacts during school holidays and the Chinese Lunar New Year. In contrast, individuals aged ≥60 years showed no clear temporal trends in their contact numbers, indicating a weaker apparent effect of these holidays on their reported contacts in this study (**Figure S7**).

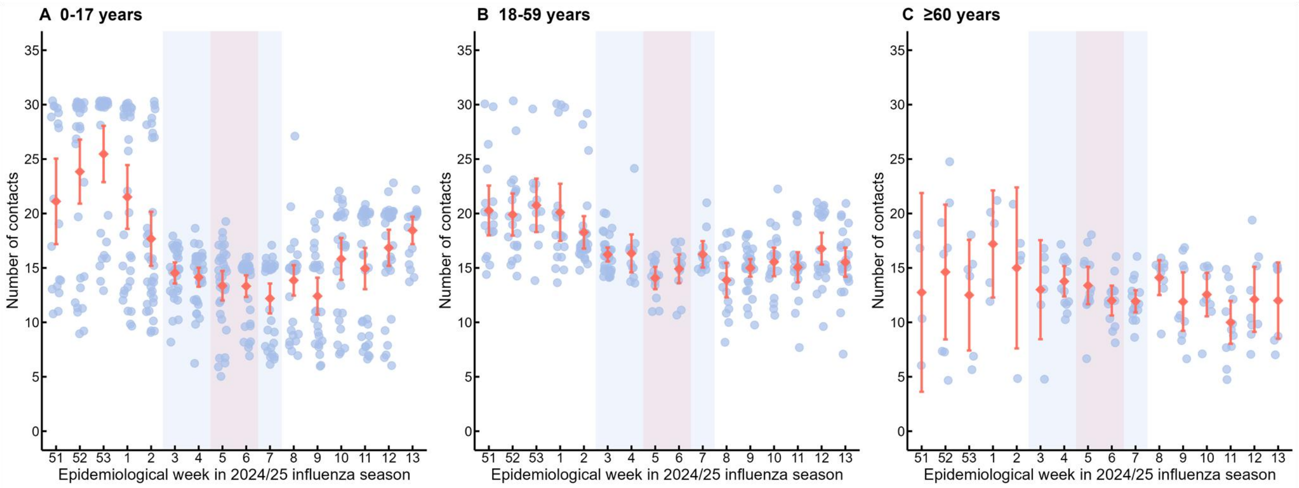

Figure S7. Contact distribution across epidemiological weeks in the 2024/25 influenza season, stratified by **age**. Panels A, B, and C showed contact distributions for the 0 to 17, 18 to 59, and ≥60

age groups, respectively. All panels were presented in the same style as in Figure S5.

We also studied the effects of different settings. As shown in **Figure S8,** the contact levels of households, leisure venues, and others remained stable throughout the study period, with no substantial fluctuations during holidays. In contrast, the reported contact numbers in schools and workplaces exhibited pronounced temporal trends. In particular, school contacts substantially decreased during holiday periods.

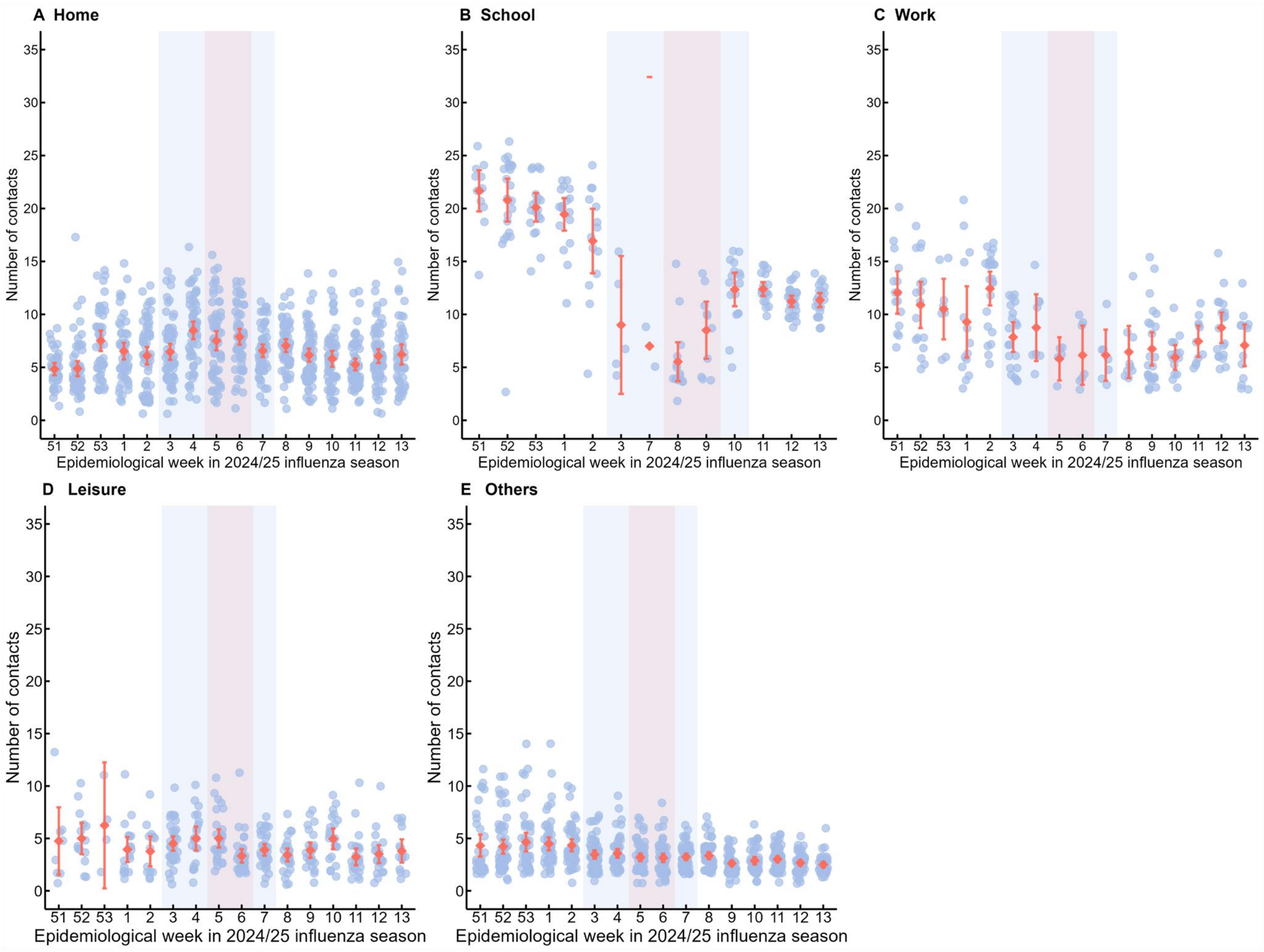

Figure S8. Contact distribution across epidemiological weeks in the 2024/25 influenza season, stratified by **contact settings**. Panels A, B, C, D, and E showed contacts that occurred in the household, school, workplace, leisure venues, and other settings, respectively. All panels were presented in the same style as in Figure S5.

In summary, reported social contact levels during the 2024/25 influenza season exhibited distinct weekly fluctuations that were closely tied to holiday schedules, with general declines observed during school breaks and the Lunar New Year. Demographically, males had slightly higher overall contact rates than females did, and females exhibited a more clustered distribution with smaller-magnitude fluctuations. By age group, both the 0- to 17-year-old and 18- to 59-year-old groups reported a decreasing trend during holidays, with the 0- to 17-year-old cohort experiencing a more pronounced effect. Conversely, individuals aged ≥60 years maintained stable contact patterns with minimal variation. Across settings, schools and workplaces were the primary drivers of overall

reported contact fluctuations, with schools registering the steepest declines during holidays. In contrast, household and leisure settings remained stable throughout the period and showed no significant variation. These observed patterns are consistent with the high interdependence between societal rhythms and holiday calendars, as school closures and the Spring Festival holiday were associated with a sharp reduction in reported commuting and social gatherings, particularly among working-age subjects and schoolchildren.

S4.3 Contact patterns and demographics by influenza status

We systematically examined the distributions of reported contact behaviour among the influenza-positive and influenza-negative groups in our outpatient sample. The results revealed minimal differences in the overall mean number of reported contacts (4.88 test positive vs. 5.31 test negative) and in the total number of contact person-hours (19.24 test positive vs. 20.69 test negative), suggesting comparable overall reported contact levels between groups. However, significant disparities in reported contacts within the test-positive group across demographic and setting strata were observed. Compared with females, males reported higher contact frequency and duration estimates (5.52 contacts and 21.62 person-hours vs. 4.26 contacts and 16.87 person-hours, respectively). With respect to age, those aged 7 to 17 years reported the highest estimates (9.99 contacts and 37.98 person-hours), followed by those aged 0 to 6 years (4.72 contacts and 19.14 person-hours). With respect to occupation, students reported the highest contact rates (8.66 contacts and 33.02 person-hours), whereas retirees and unemployed subjects reported the lowest (2.59 contacts and 9.99 person-hours). Households with four members recorded the highest contact levels (6.25 contacts and 24.68 person-hours). Finally, across settings, reported school contacts were highest (8.26 contacts and 31.82 person-hours), household levels remained stable, and recreational and other settings showed minimal contact.

While the overall reported contact patterns were similar between the test-positive and test-negative groups, significant disparities existed within the test-positive group. Students were the highest-risk group for exposure, suggesting that schools should be prioritized for preventive measures, while households represent effective venues for interventions. Detailed data on contact numbers and person-hours, including standard deviations (SDs), stratified by the subjects' characteristics, test results, and contact settings, are provided in **Table S4**.

Table S4. Multiple-censoring negative binomial regression model of daily recorded contact counts and person-hours, stratified by testing status (positive or negative) and individual characteristics.

	**Test-positive subjects**		**Test-negative subjects**	
	**Average number of contacts (SD)**	**Average contact person-hours (SD)**	**Average number of contacts (SD)**	**Average contact person-hours (SD)**
Total	4.88 (3.41)	19.24 (12.90)	5.31 (4.09)	20.69 (15.58)
Sex				
Female	4.26 (2.65)	16.87 (10.08)	5.34 (4.07)	20.82 (15.42)
Male	5.52 (4.16)	21.62 (15.67)	5.27 (4.10)	20.55 (15.78)

Age group				
<6 yr	5.11 (4.25)	20.37 (15.53)	3.47 (2.73)	14.36 (9.76)
7-17 yr	9.99 (4.55)	37.98 (16.92)	9.17 (5.76)	35.95 (23.42)
18-59 yr	4.49 (2.68)	17.60 (10.40)	5.54 (3.48)	20.66 (14.04)
≥60 yr	2.89 (1.90)	11.45 (4.65)	2.94 (1.86)	11.08 (4.82)
Ethnicity				
Han	5.29 (3.74)	19.97 (13.94)	4.99 (3.78)	19.15 (14.55)
Other ethnicities	4.55 (3.14)	18.40 (12.10)	5.53 (4.29)	21.75 (16.26)
Education				
Secondary	5.07 (3.79)	19.92 (14.27)	5.02 (4.05)	20.00 (15.42)
Postsecondary	4.58 (2.80)	17.81 (10.54)	5.99 (3.97)	22.33 (15.72)
Type of profession				
Preschool	4.72 (3.91)	19.14 (14.53)	3.47 (2.76)	14.42 (9.86)
Student	8,66 (4.93)	33.02 (19.54)	8.72 (5.73)	34.17 (23.14)
Employed	4.96 (2.76)	19.29 (10.63)	5.41 (3.24)	20.13 (13.21)
Unemployed	2.59 (1.81)	9.99 (4.54)	3.06 (1.97)	11.46 (5.94)
Retired	3.10 (2.05)	12.54 (5.07)	3.07 (1.95)	11.36 (4.94)
Unknown	0.00 (0.00)	0.00 (0.00)	4.49 (5.32)	18.56 (23.29)
Household size				
1	4.71 (3.92)	18.67 (17.02)	5.38 (5.99)	14.97 (26.44)
2	2.57 (2.17)	8.24 (5.64)	3.78 (3.06)	12.64 (10.77)
3	4.44 (3.28)	16.23 (11.95)	3.97 (3.54)	15.20 (12.39)
4	6.25 (4.83)	24.68 (19.04)	6.48 (4.95)	25.80 (19.35)
≥5	3.91 (2.29)	15.76 (6.59)	4.96 (3.14)	19.27 (11.53)
Contact setting				
Household	2.31 (1.62)	9.75 (3.86)	2.21 (1.54)	9.49 (3.75)
School	8.26 (4.09)	31.82 (16.26)	9.13 (4.30)	36.02 (18.75)
Workplace	3.38 (2.92)	13.04 (13.24)	3.61 (3.70)	13.20 (17.34)
Leisure	0.07 (0.87)	0.13 (0.43)	0.16 (0.67)	0.43 (1.13)
Others	0.03 (0.16)	0.11 (1.24)	0.02 (0.26)	0.05 (0.58)
Influenza vaccination status in the past 24 months				
Unvaccinated	4.81 (3.33)	18.87 (12.48)	5.21 (3.99)	20.32 (15.20)
Vaccinated	6.27 (5.63)	26.58 (24.17)	6.87 (5.48)	26.05 (20.98)

S4.4 Associations between contact type, setting, age, and influenza status

Our analysis of contact patterns by socioenvironmental context, behavioural characteristics, subject age, and influenza status revealed significant differences in the distribution of contact locations, types, frequency, and duration across age groups and between test outcomes.

S4.4.1. Analysis of age-specific contact patterns by setting

Overall, the household was the predominant reported contact setting across all age groups in our study, accounting for more than 50% of all contacts in both the 0- to 5-year-old and over 60-year-old groups. In school settings, contact was concentrated almost exclusively among those aged 6 to 20 years, while workplace contact was most common among those aged 21 to 45 years. Age-stratified analysis in **Figure S9** demonstrates that the difference in contact proportions within each bar (collective school, workplace, and leisure settings) of test-positive and test-negative subjects is clustered in some age groups. The overall contact proportion for positive subjects across these specific age groups was greater.

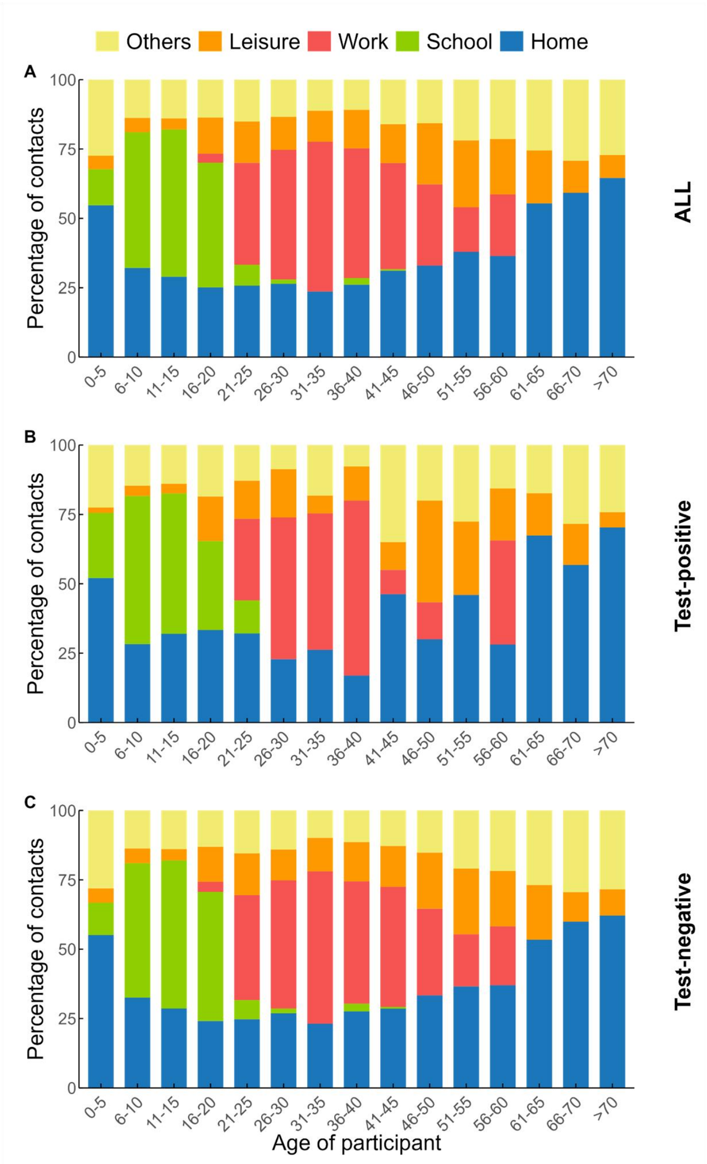

Figure S9. Contact patterns by age group and contact scenario.

S4.4.2. Analysis of the influence of contact setting, frequency, and duration on contact type

Chi-square tests within our study sample revealed significant differences (p value < 0.05) between test-positive and test-negative subjects in terms of the distribution of both physical and nonphysical contact by setting and frequency and the distribution of physical contact only by duration. The proportional data underlying these tests are presented in **Figure S10** and **Table S5**.

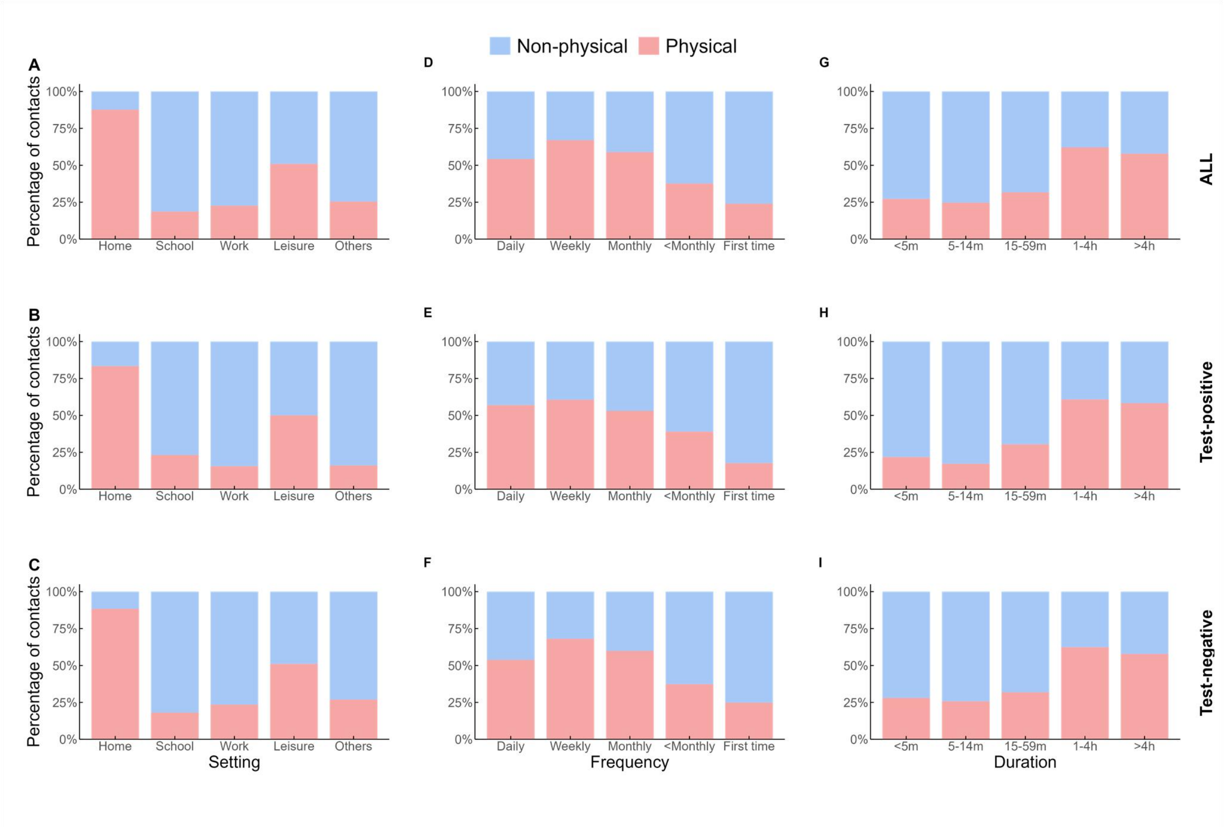

Figure S10. Distribution of contact proportions for different populations by location, frequency, and duration

Table S5. Analysis of the distribution and differences in contact setting, frequency, and duration between test-negative and test-positive subjects, stratified by contact type.

		Physical			Nonphysical		
		**Test-negative**	**Test-positive**	*p*-value	**Test-negative**	**Test-positive**	*p*-value
Setting	Household	4493	700	<0.001	590	140	<0.001
	School	470	75		2127	249	
	Workplace	385	32		1254	173	
	Leisure	635	83		609	83	
	Others	675	61		1835	318	
Frequency	Daily	3522	520	<0.001	3019	393	<0.001
	Weekly	1722	246		809	160	
	Monthly	602	86		404	76	
	<Monthly	158	39		265	61	
	First time	623	58		1885	271	
Duration	<5 m	220	25	0.034	565	90	0.061
	5-14 m	300	30		865	144	
	15-59 m	532	70		1139	160	
	1-4 h	2023	330		1222	213	
	>4 h	3552	494		2591	354	

S4.4.3 Association of contact frequency and setting with influenza

Analysis of our sample revealed distinct contact patterns by setting. Among reported household contacts, 50% occurred daily, and more than 90% lasted more than 1 hour. In the ‘others’ setting, more than 75% of reported contacts were first-time encounters typically lasting less than 15 minutes. Schools had the greatest proportion of reported contacts that lasted longer than 4 hours (**Figure S11**). Notably, no significant distributional differences in contact frequency or duration were observed between test outcomes across any strata, which suggests that contact duration alone does not inherently increase infection risk in these settings. However, a significant association between contact duration and positive test results was observed in household (p-value = 0.017), workplace (p-value = 0.006), and leisure (p-value < 0.001) settings (see **Table S6**).

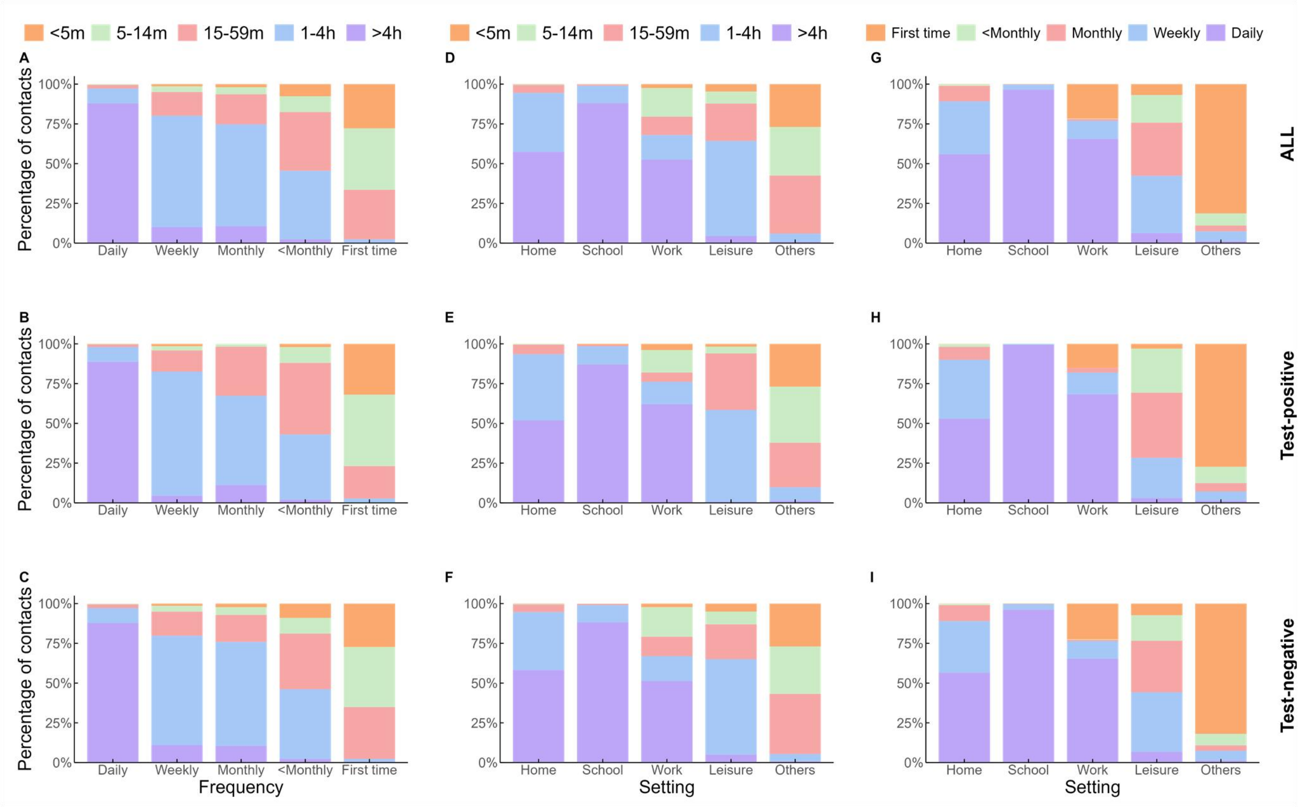

Figure S11. Relationships between contact location, duration, and frequency.

Table S6. Distribution and statistical comparison of contact duration by setting and frequency between influenza test-positive and test-negative individuals.

Category	Group	Duration	Test-negative	Test-positive	*p*-value
Setting	Household	<5 m	10	1	0.017
		5-14 m	21	4	
		15-59 m	241	50	
		1-4 h	1856	349	
		>4 h	2955	436	
	School	<5 m	2	2	0.164
		5-14 m	4	0	
		15-59 m	20	3	
		1-4 h	279	37	
		>4 h	2292	282	
	Workplace	<5 m	37	8	0.006
		5-14 m	305	29	
		15-59 m	200	12	
		1-4 h	256	29	
		>4 h	841	127	
	Leisure	<5 m	63	3	<0.001
		5-14 m	100	7	
		15-59 m	272	59	
		1-4 h	747	96	
		>4 h	62	1	
	Others	<5 m	677	102	<0.001
		5-14 m	749	134	
		15-59 m	949	106	
		1-4 h	117	32	
		>4 h	18	5	
Frequency	Daily	<5 m	3	2	0.112
		5-14 m	32	2	
		15-59 m	153	14	
		1-4 h	613	85	
		>4 h	5740	810	
	Weekly	<5 m	36	6	<0.001
		5-14 m	94	11	

		15-59 m	382	54	
		1-4 h	1740	317	
		>4 h	279	18	
	Monthly	<5 m	23	0	<0.001
		5-14 m	49	3	
		15-59 m	171	50	
		1-4 h	657	91	
		>4 h	106	18	
	<Monthly	<5 m	38	2	0.110
		5-14 m	42	10	
		15-59 m	148	45	
		1-4 h	185	41	
		>4 h	10	2	
	First time	<5 m	685	105	<0.001
		5-14 m	948	148	
		15-59 m	817	67	
		1-4 h	50	9	
		>4 h	8	0	

S4.5 Age-specific effects of contact settings on influenza and their distributions

To determine whether the association between contact exposure and influenza test results varies across age groups, we stratified our analysis by age. First, as shown in **Figure S12**, we compared the distributions of the total number of contacts and contact duration between test-positive and test-negative individuals in the overall population. The fitted curves for both groups exhibited right-skewed distributions, with a higher peak observed in the test-positive group. Moreover, compared with test-negative individuals, test-positive individuals reported a significantly higher contact frequency. The methodology for these results is described in the **Supplementary Materials**, **S4.1.1**.

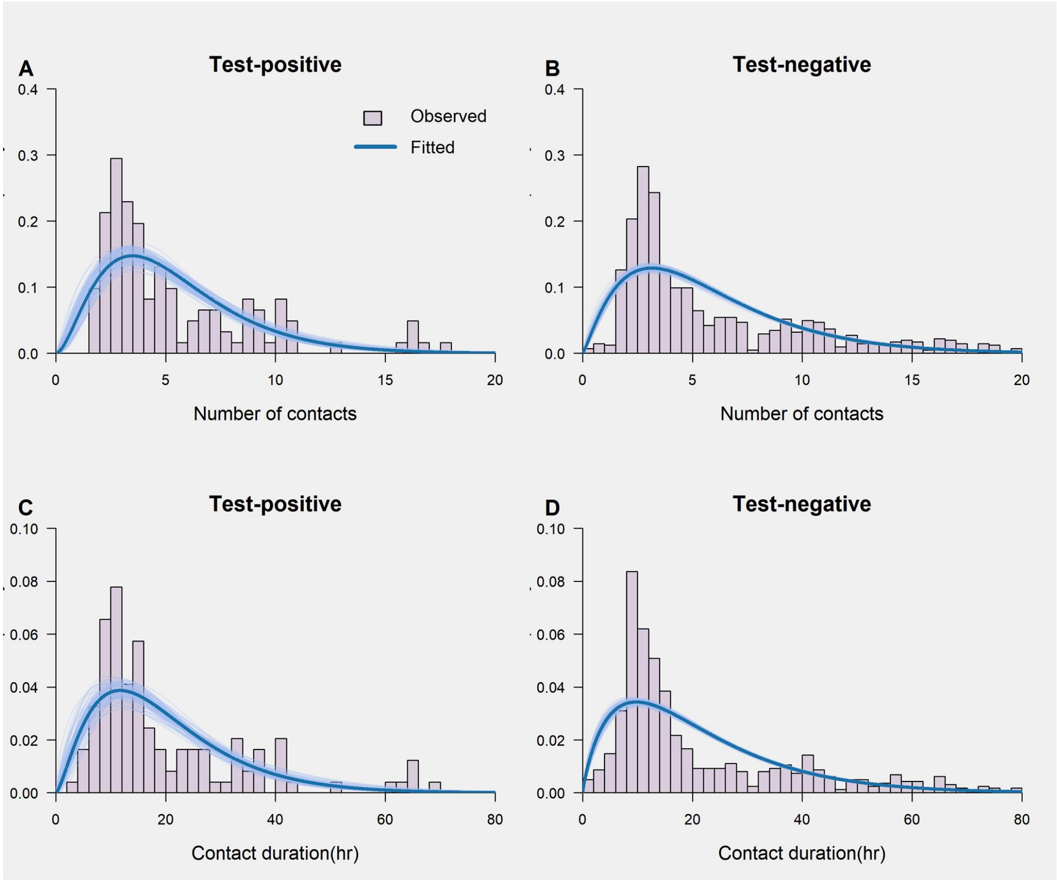

Figure S12. Comparison of gamma distribution fits for contact frequency and duration between test-positive and test-negative subjects. Panel A showed the number of contacts among test-positive subjects. Panel B showed the number of contacts among test-negative subjects. Panel C showed the contact person-hours for test-positive subjects. Panel D showed the contact person-hours for test-negative subjects.

We further performed a stratified analysis by contact setting to quantify its impact on the test outcomes within specific age groups. This analysis was conducted for the overall population and separately for the three age groups (0 to 17, 18 to 59, and ≥60 years). In the overall sample, test-positive subjects reported higher contact frequencies than test-negative subjects did at low contact levels (0 to 10 contacts), and they had significantly higher frequencies across all settings (**Figures S13A**, and **S13B**). A similar trend was observed for duration, with positive subjects having significantly longer contact hours in household, school, and workplace settings within the 0–20 hour range (**Figures S13C**, and **S13D**).

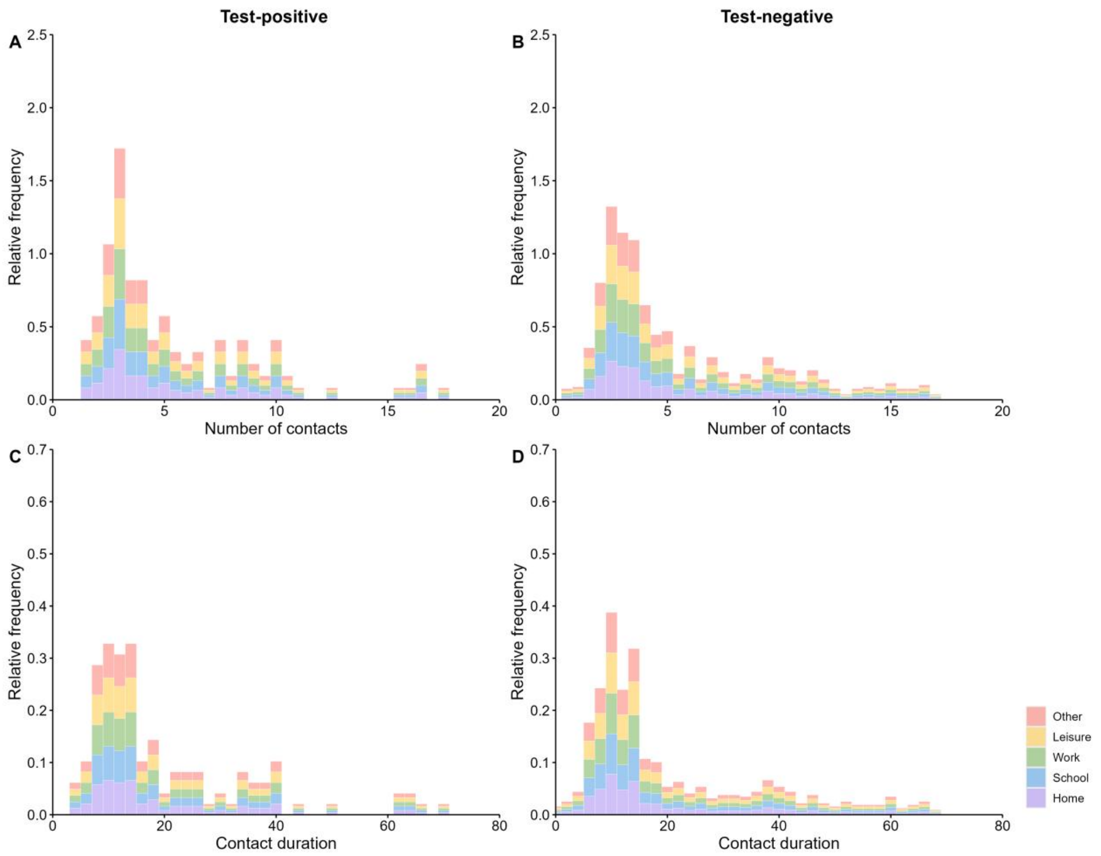

Figure S13. Comparison of contact frequency and duration distributions between test-positive and test-negative subjects. Panel A showed the number of contacts among test-positive subjects. Panel B showed the number of contacts among test-negative subjects. Panel C showed the contact person-hours for test-positive subjects. Panel D showed the contact person-hours for test-negative subjects.

With respect to the 0–17 age group, test-positive subjects had significantly higher contact frequencies across all settings when contact events involved more than 5 people, with pronounced differences in household and school settings (**Figures S14A**, and **S14B**). As shown in **Figures S14C** and **S14D**, for contact durations exceeding 20 hours, positive subjects also exhibited higher contact frequencies in household, school, and leisure settings than negative subjects did.

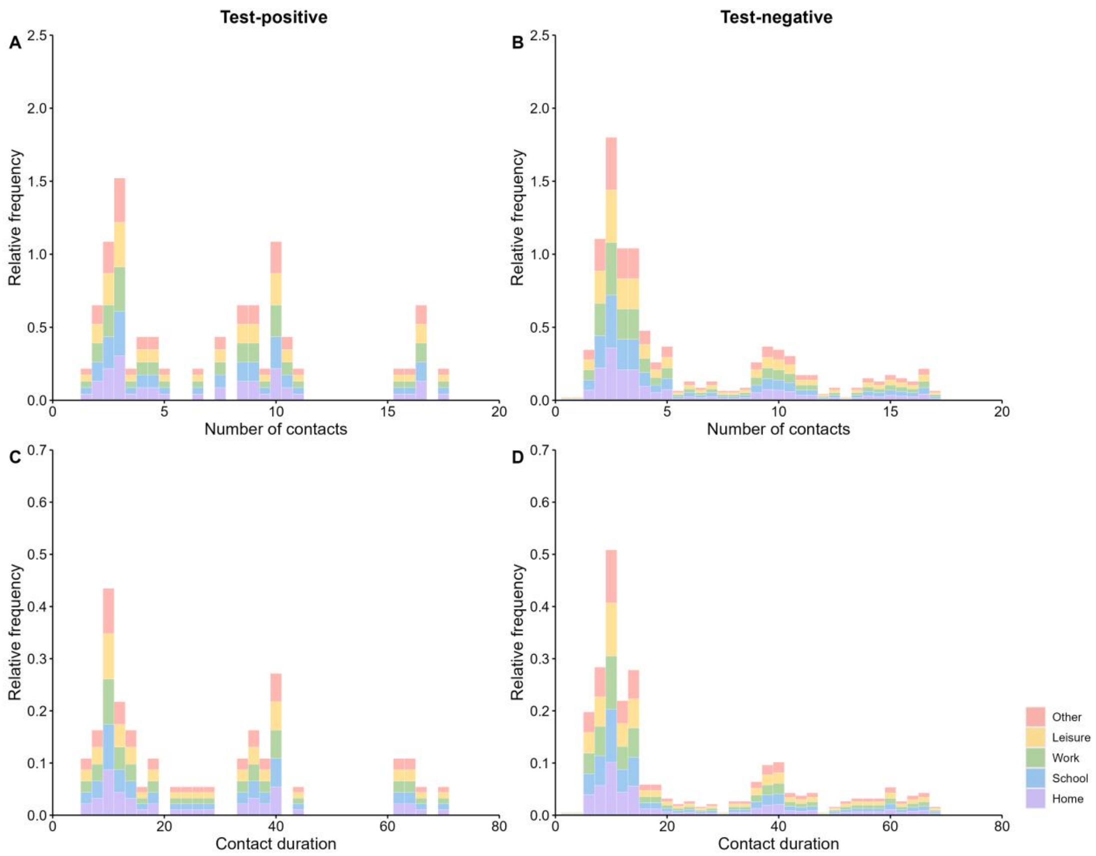

Figure S14. Comparison of contact frequency and duration distributions between test-positive and test-negative subjects **aged 0 to 17 years**. All panels were presented and arranged as in Figure S13.

Among subjects aged 18–59 years, significant differences emerged in the relative frequency of contact across settings (household, school, and workplace) between the positive and negative groups at contact levels of approximately 5 or more people, with positive subjects showing markedly higher frequencies (**Figures S15A**, and **S15B**). A similar pattern was observed for contact duration, where test-positive subjects exhibited higher relative contact frequencies in these same settings at durations of approximately 20 hours or longer than test-negative subjects did (**Figures S15C**, and **S15D**).

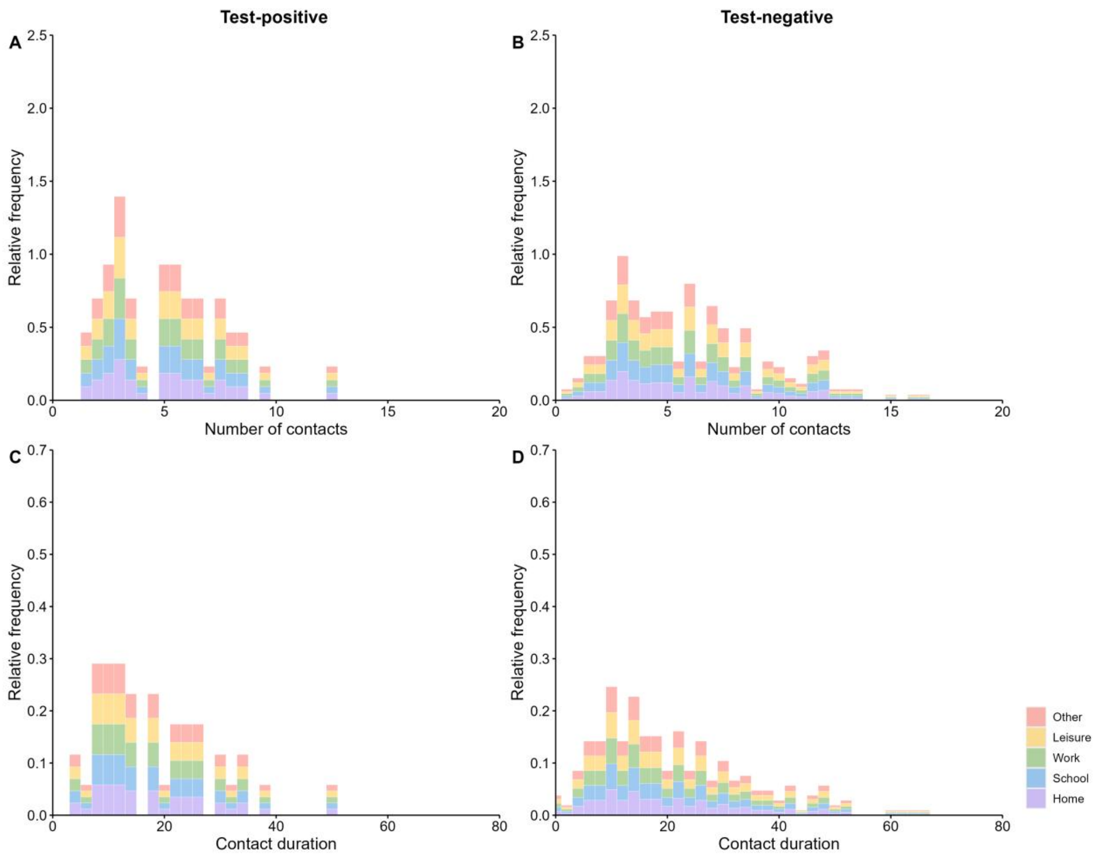

Figure S15. Comparison of contact frequency and duration distributions between test-positive and test-negative subjects **aged 18 to 59 years**. All panels were presented and arranged as in Figure S13.

For subjects aged ≥60 years, within the contact range of 0–5 people, the relative frequency distributions of household and other settings differed significantly between the test-positive and test-negative groups, with both settings exhibiting substantially higher relative frequencies in the positive group (**Figures S16A**, and **S16B**). Similarly, within the 0–20-hour contact duration range, the relative frequencies of household and other settings significantly differed between the two groups, again showing higher relative frequencies for these two settings in the positive group (**Figures S16C**, and **S16D**).

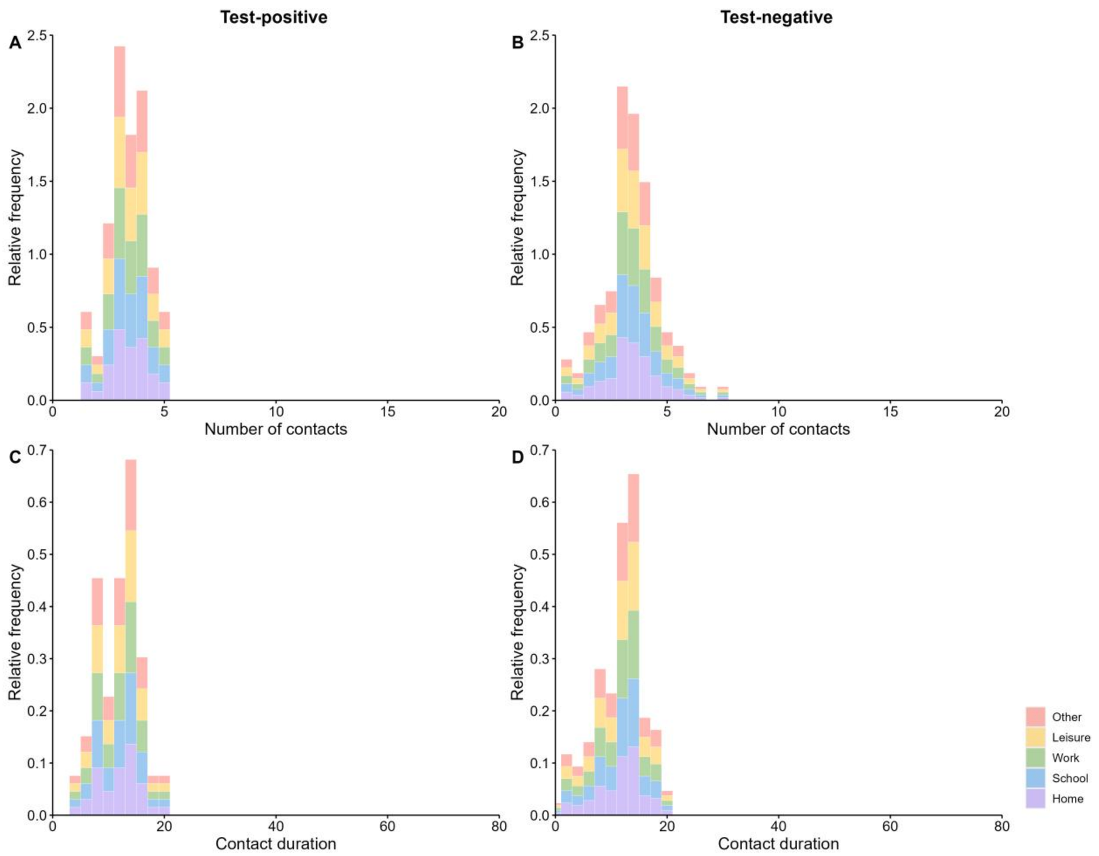

Figure S16. Comparison of contact frequency and duration distributions between test-positive and test-negative subjects **aged ≥60 years**. All panels were presented and arranged as in Figure S13.

S4.6 Association analysis of influenza and contact patterns across demographic subgroups

We conducted analyses stratified by sex, household size, and contact setting to assess their potential influence on the relationship between influenza test results and contact patterns across age groups in our sample. First, we analysed the number of contacts and contact duration among different age groups that were stratified by test status. Second, the analysis was extended to evaluate age-standardized differences and the distribution of subjects by test status across these age groups. Regression models were employed to examine the disparities in the number of contacts and contact duration between test-positive and test-negative subjects within the same age groups. Significant differences in reported contact patterns were observed between positive and negative subjects across the examined subgroups. The methodology for these results is described in the **Supplementary Materials**, **S4.1.2**.

<u>S4.6.1. Analysis of the impact of sex stratification on contact number and duration</u>

Among males, those aged 0–20 years who tested positive reported more contacts and longer contact durations than their negative counterparts did. For all males, both contact and duration tended to decrease with advancing age **(Figure S17**). The age-standardized difference in contacts (the difference in contact values between test-positive and test-negative subjects) was positive for the 0–15 age group, indicating that their reported contact measures were above the average level of our study population (**Figures S18A**, and **S18B**). The regression coefficients greater than 1 for the 0 to 5, 6 to 15, and over 65 age groups (**Figures S18D**, and **S18E**) suggest that, within our sample, positive males in these groups exhibited greater increases in contact number and duration than negative males

did.

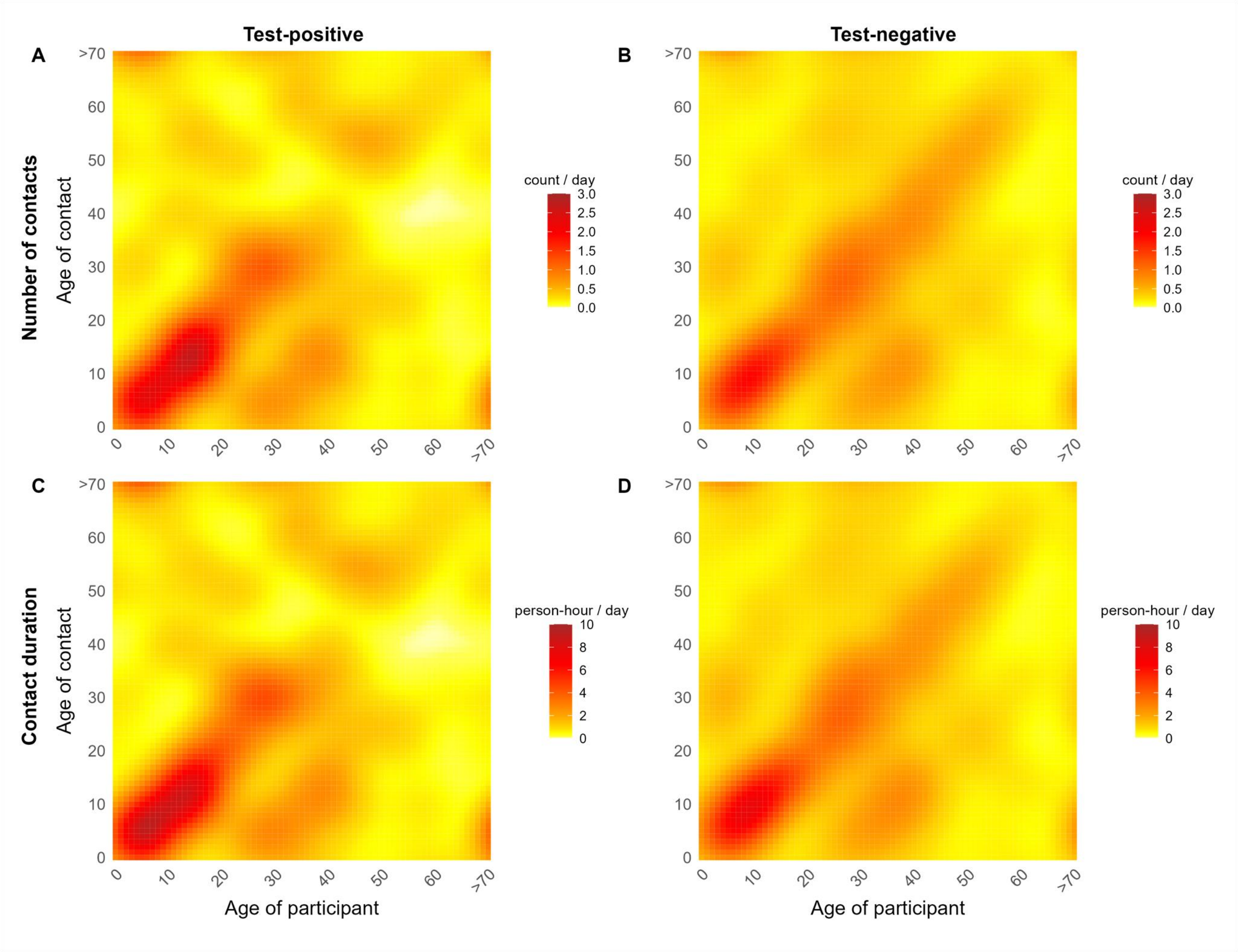

Figure S17. Age-specific contact matrix for the **male** group. Panel A showed the number of contacts for test-positive subjects. Panel B showed the number of contacts for test-negative subjects. Panel C showed the contact person-hours for test-positive subjects. Panel D showed the contact person-hours for test-negative subjects.

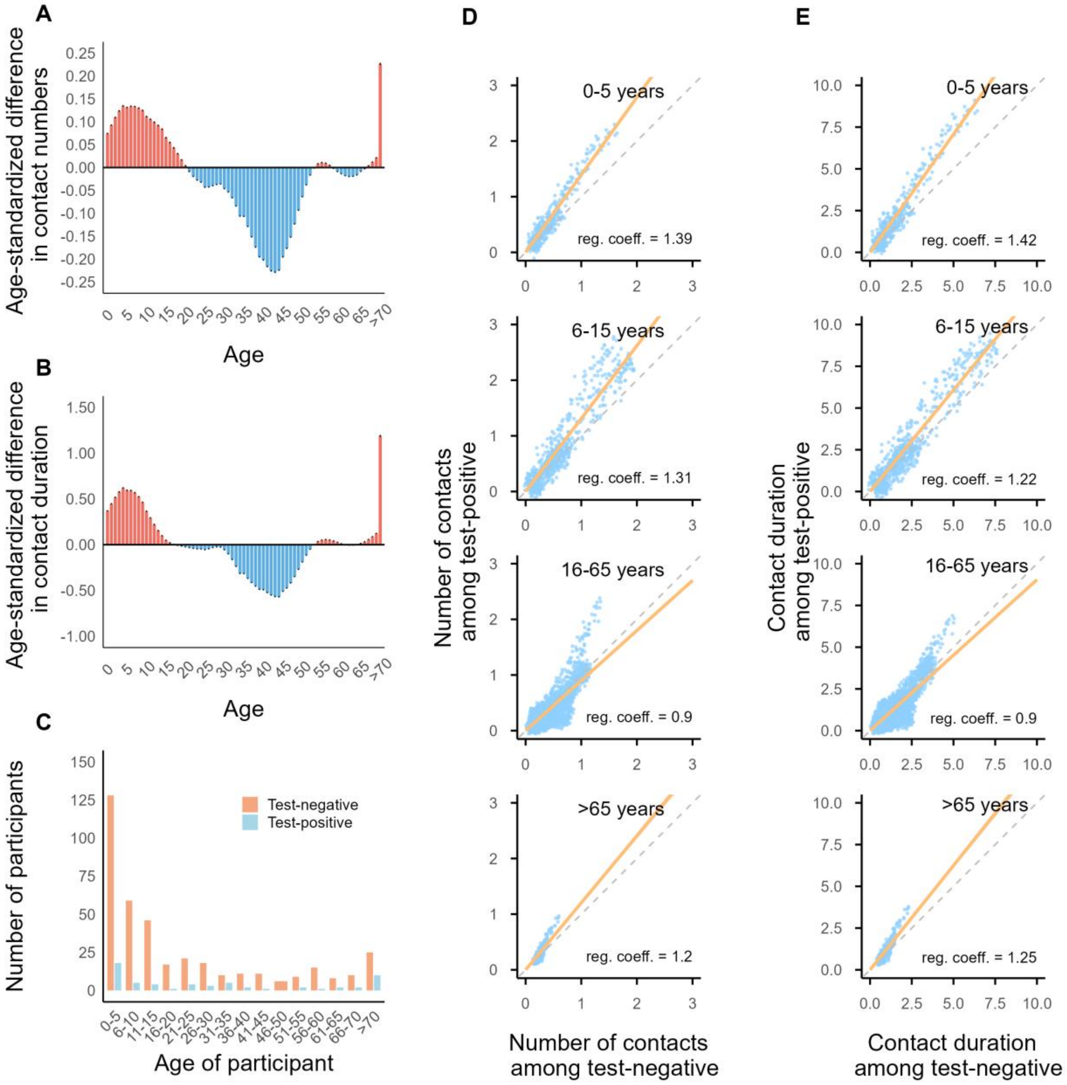

Figure S18. Contact patterns and testing outcomes by age group among **males**. Panel A showed the age-standardized distribution of the difference in contact number across different age groups. Panel B showed the distribution of contact duration. Panel C showed the distribution of test-negative and test-positive subjects across different age groups. Panel D showed a comparison of the number of contacts between test-negative and test-positive subjects by age group. Panel E showed a similar comparison for contact duration across age groups.

Women aged 20 to 40 years who tested positive reported a greater scope of contact than did those who tested negative (**Figure S19**). As shown in **Figure S20A** and **S20B**, the contact numbers and contact hours in most female age groups were less than the study population's average. Furthermore, the regression coefficients varied across age groups (**Figures S20D**, and **S20E**), indicating that among females, changes in the contacts of subjects who tested negative influenced those who tested positive to different extents across ages, with a stronger effect observed in the older group over 65 years of age.

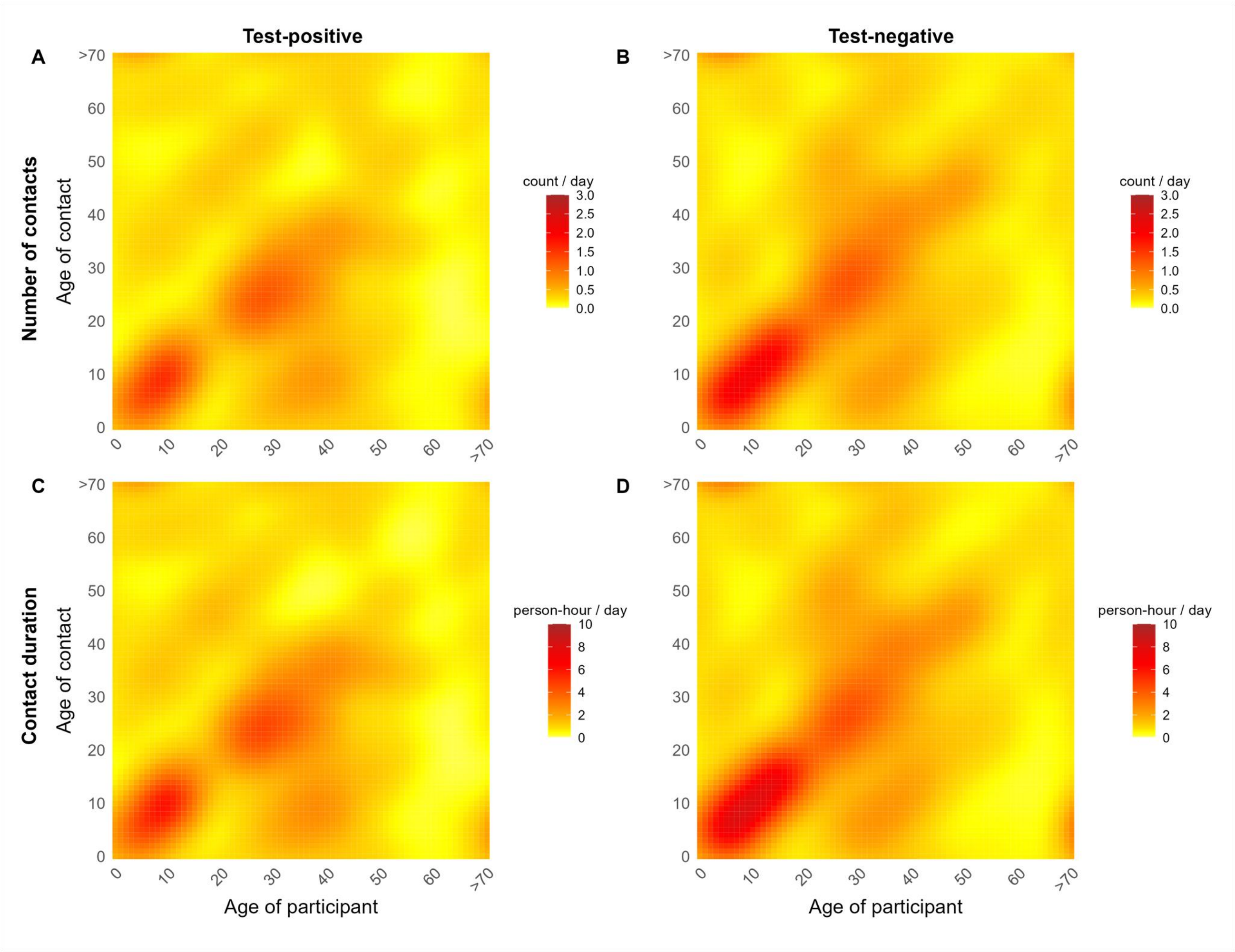

Figure S19. Age-specific contact matrix for the **female** group. All panels were presented and arranged as in Figure S17.

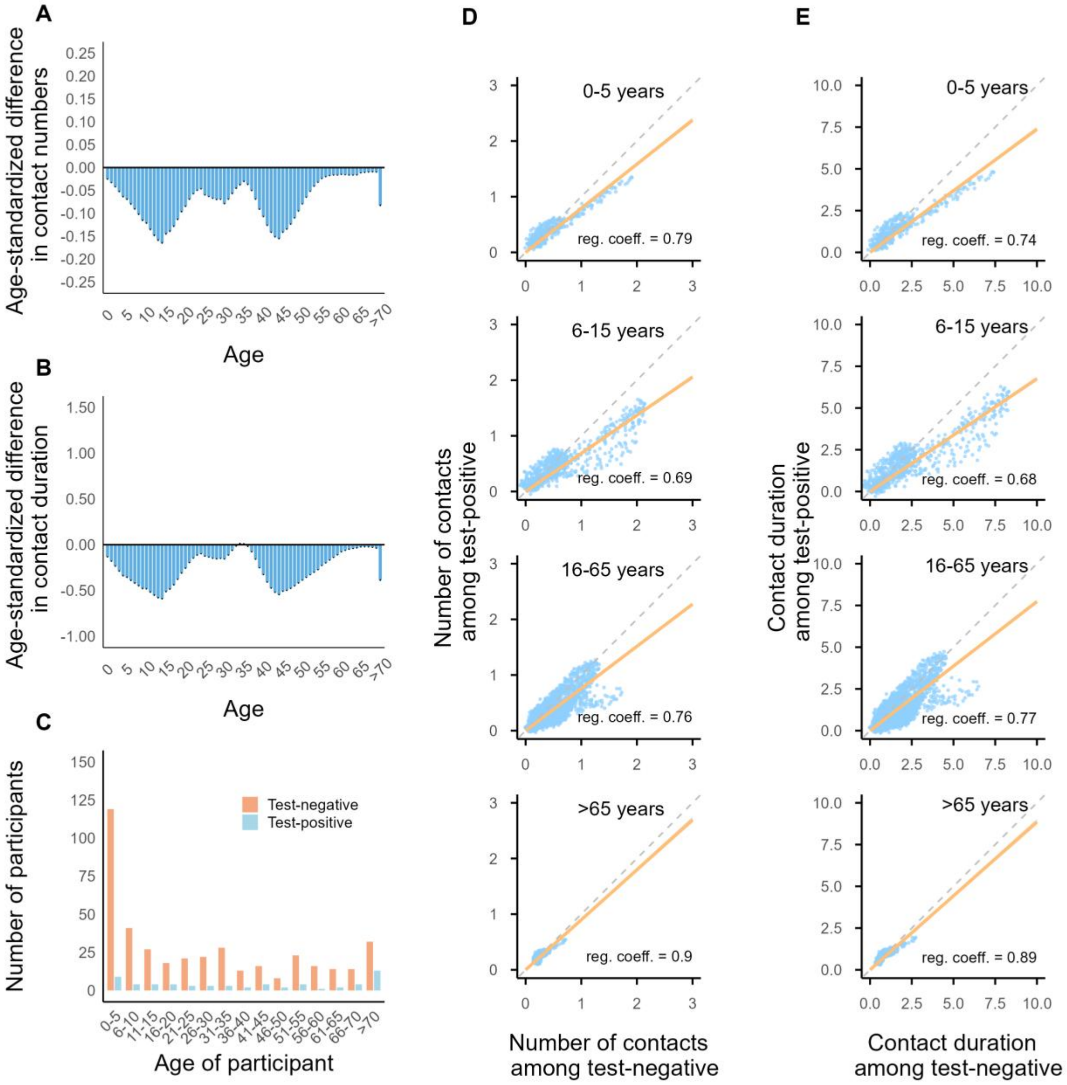

Figure S20. Contact patterns and testing outcomes by age group among **females**. All panels were presented and arranged as in Figure S18.

Across both the test-positive and test-negative groups, males reported a higher contact frequency and longer contact duration than females did. Further regression analysis revealed that across age groups, the regression coefficients for both the number of contacts and duration when test-positive versus test-negative males were compared were consistently greater than the corresponding coefficients observed for females. These results indicate that within the same age group, the contact patterns of test-positive males respond more sensitively to variations in the contact patterns of test-negative males compared with the same association observed among females.

S4.6.2 Analysis of the impact of household size on contact number and duration

As shown in **Figure S21**, for subjects who were in households with 1–3 members, both the number of contacts and the duration of contact among influenza-positive subjects peaked in the 15–35 age group. For this household size category, the age-standardized differences for the contact number and duration were predominantly negative, indicating that these were below the average level (**Figures S22A**, and **S22B**). Furthermore, regression analysis between test-positive and test-negative subjects yielded coefficients of less than 1 for both contact number and duration. These

findings indicate that the increase in contact number and duration among positive subjects was less pronounced in response to increases observed among negative subjects (**Figures S22D**, and **S22E**).

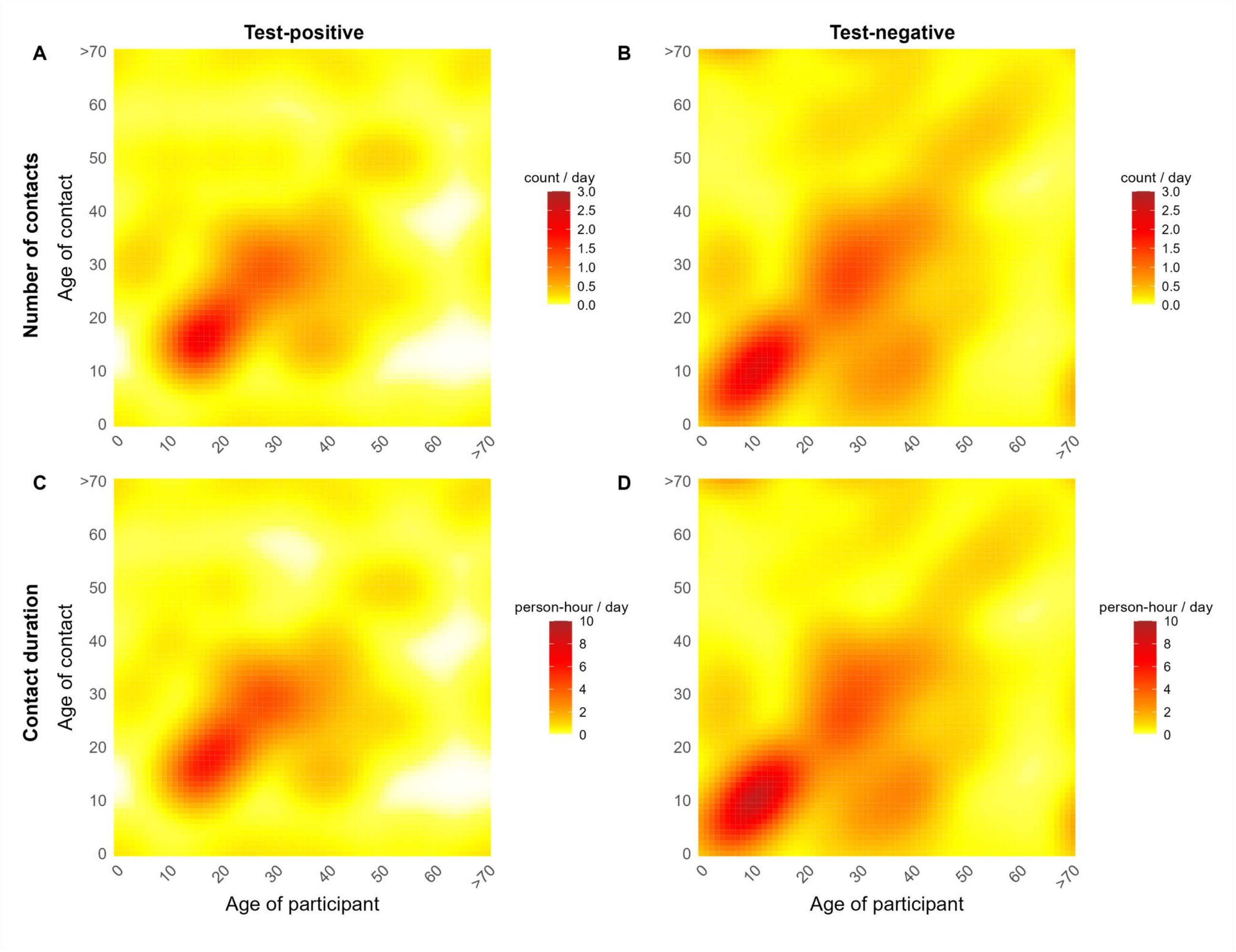

Figure S21. Age-specific contact matrix for households with **≤3 members**. All panels were presented and arranged as in Figure S17.

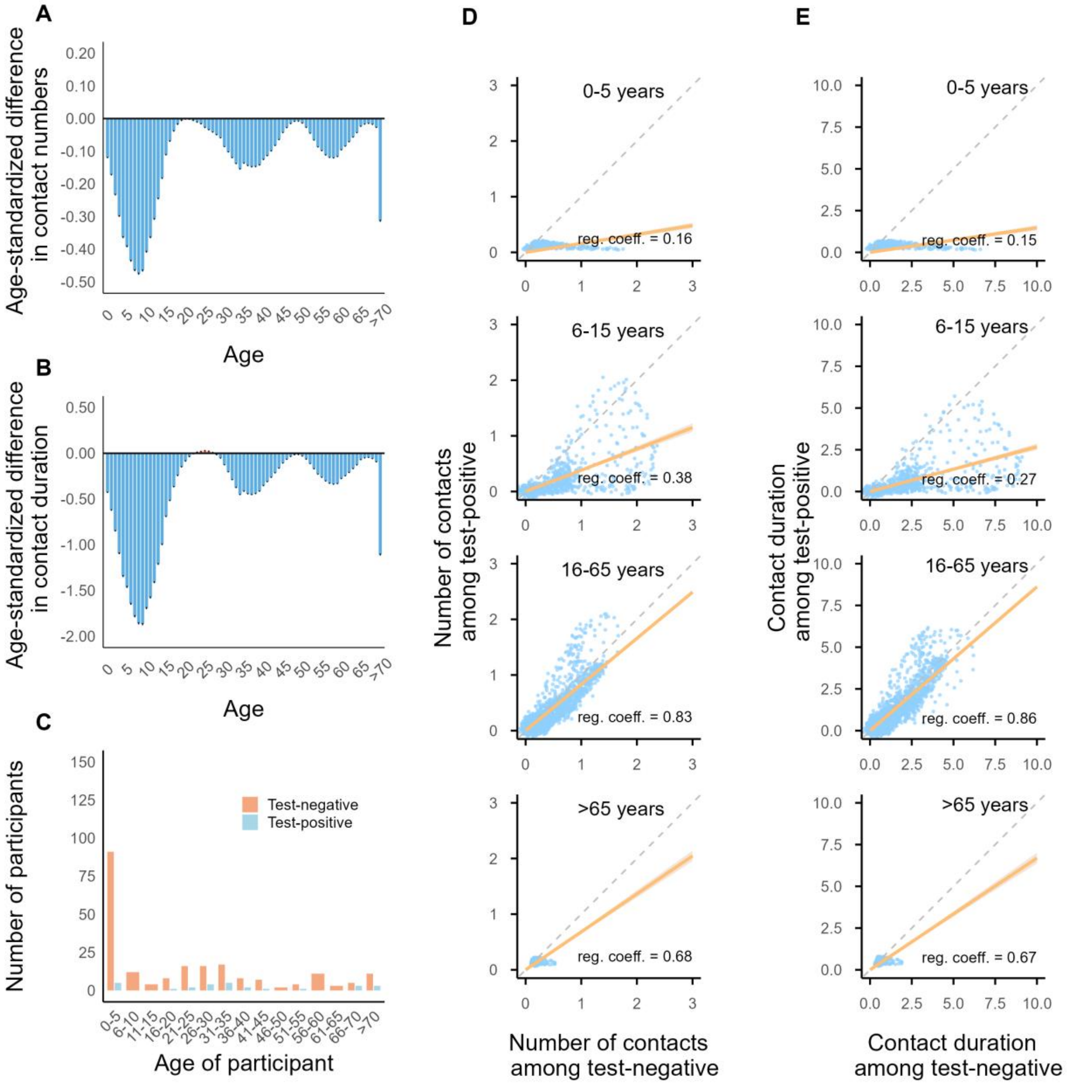

Figure S22. Contact patterns and testing outcomes by age group in households with **≤3 members**. All panels were presented and arranged as in Figure S18.

Households with four members accounted for a relatively high proportion of subjects. Within these households, those aged 0–15 years had the greatest number of contacts and the longest contact durations, in addition to a slightly higher infection incidence among test-positive individuals (**Figure S23**). The age-standardized differences for both contact number and duration were predominantly positive in this age group (**Figures S24A**, and **S24B**), indicating reported contact levels above the average of the study sample. Moreover, regression coefficients greater than 1 were observed for the 0–5-year-old and 6–15-year-old subgroups (**Figures S24D**, and **S24E**), indicating that the increase in contact number and duration among test-positive subjects was more responsive to corresponding increases among test-negative subjects.

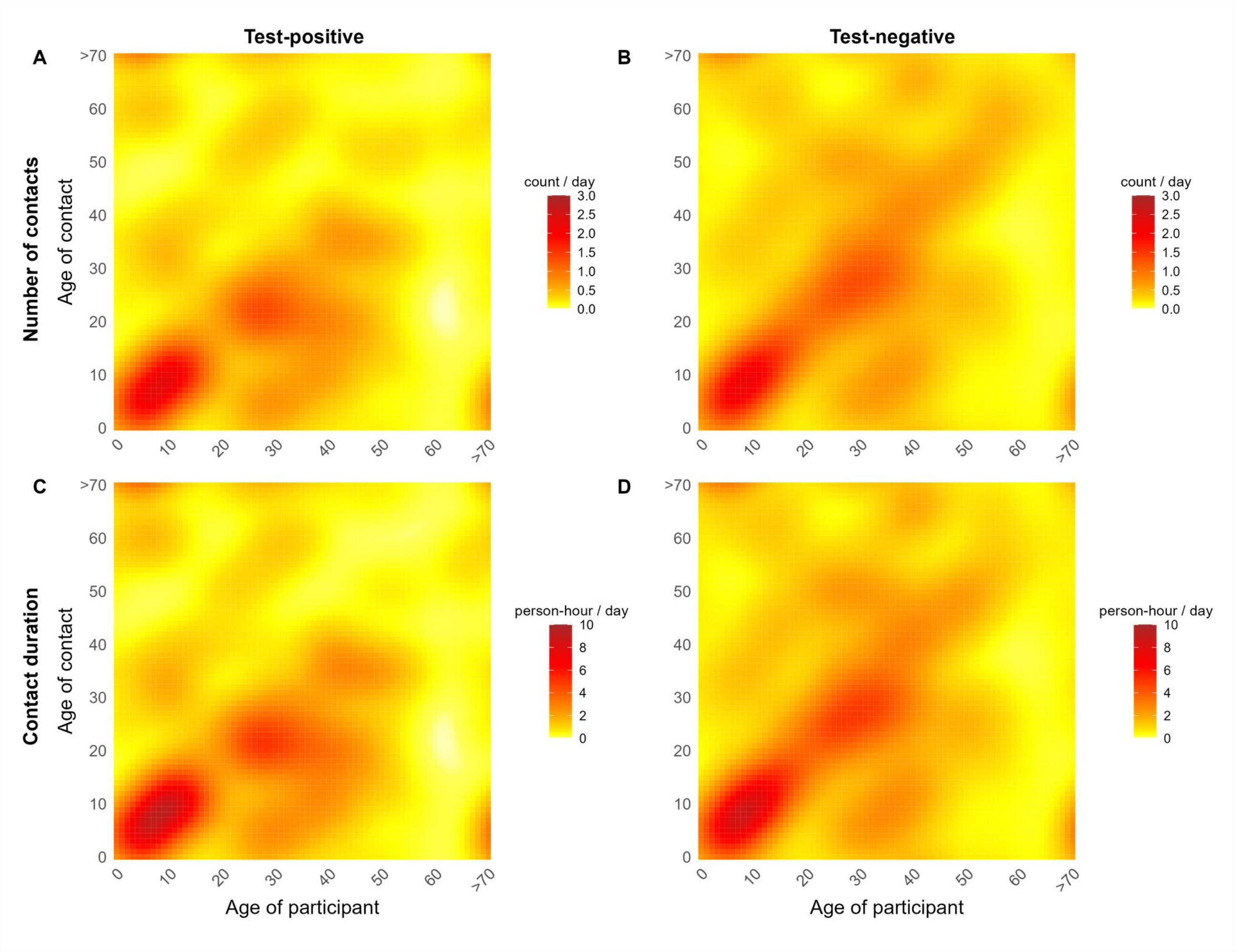

Figure S23. Age-specific contact matrix for households with **4 members**. All panels were presented and arranged as in Figure S17.

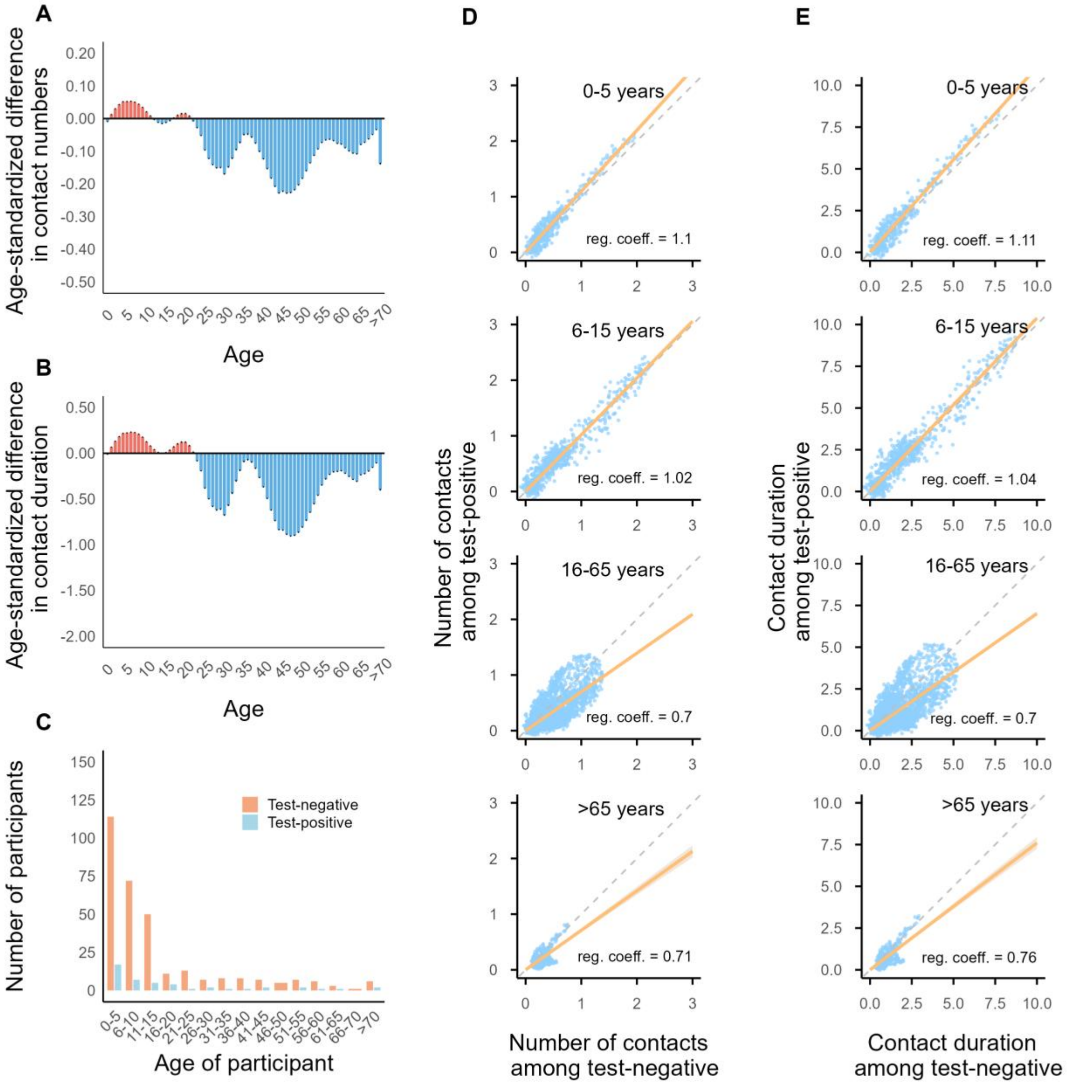

Figure S24. Contact patterns and testing outcomes by age group in households with **4 members**. All panels were presented and arranged as in Figure S18.

In households with more than five members, the <20-year-old age group had a greater number of contacts in both the test-positive and test-negative groups (**Figure S25**). As shown in **Figure S26**, positive age-standardized differences in contact number and duration were observed for both the 0–5 and >65 age groups, along with regression coefficients greater than 1.

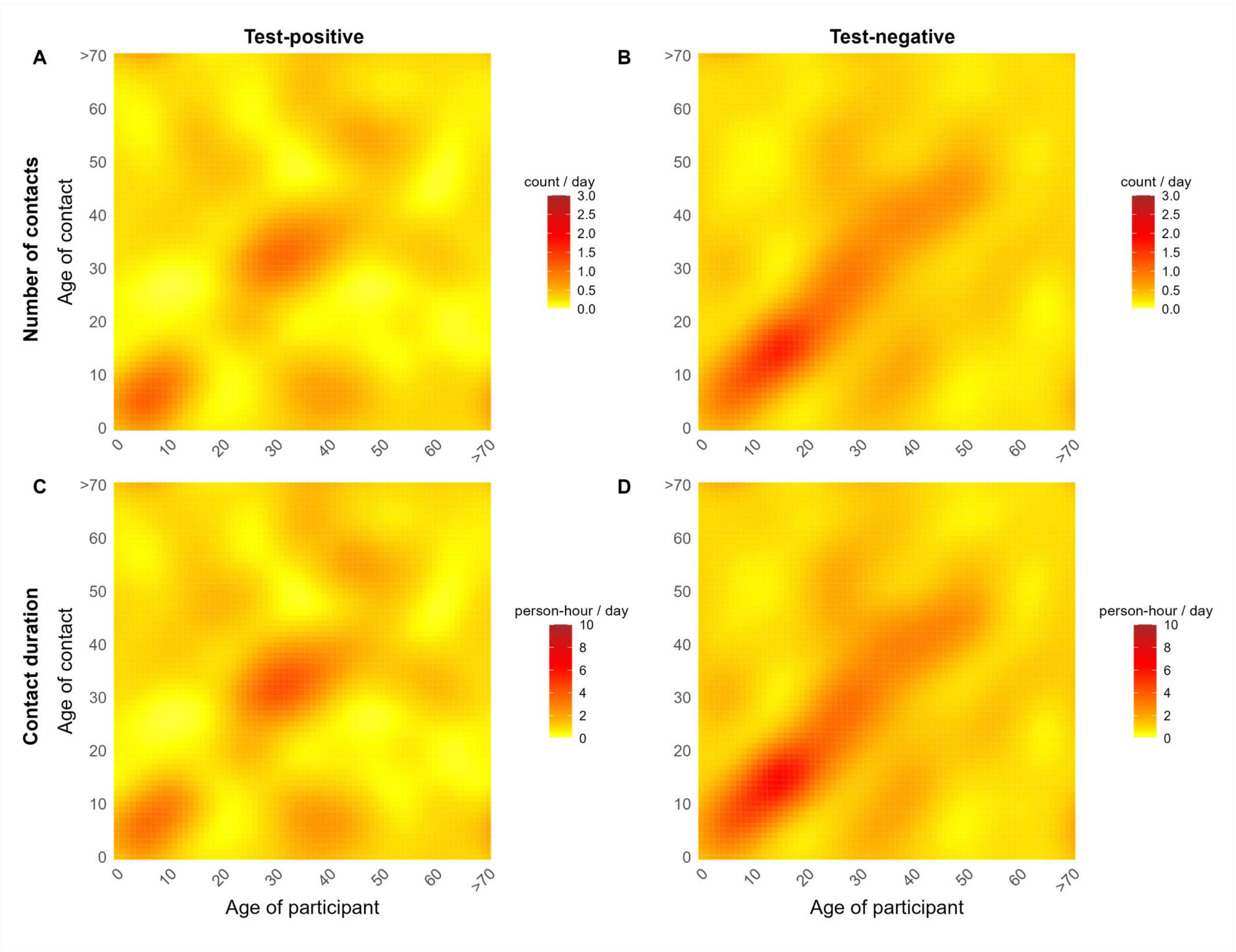

Figure S25. Age-specific contact matrix for households with **≥5 members**. All panels were presented and arranged as in Figure S17.

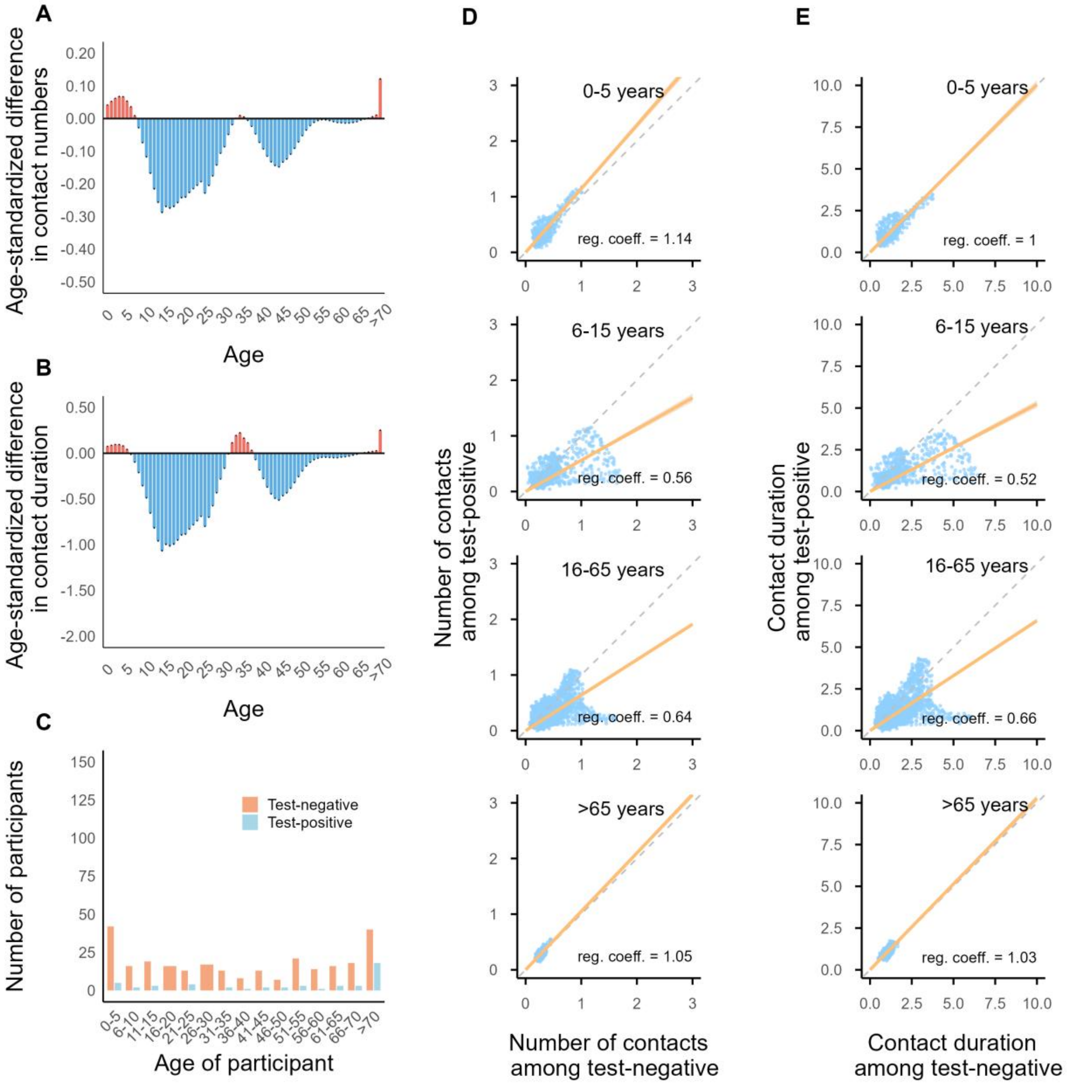

Figure S26. Contact patterns and testing outcomes by age group in households with **≥5 members**. All panels were presented and arranged as in Figure S18.

Among all healthcare-seeking ILI subjects, influenza infection risk profiles varied across household sizes, indicating the potential need for demographically targeted interventions. In 4-person households, children and adolescents (0 to 18 years) reported the highest contact intensity both inside and outside the household, corresponding to their elevated test positivity rates and highlighting their role in transmission networks. In large households (≥5 members), young children (0 to 5 years) and older subjects (>65 years) reported high contact frequency, prolonged exposure duration, and reduced immune competence, leading to a significantly higher infection risk than in other age groups.

S4.6.3. Analysis of the impact of contact settings on contact number and duration

The analysis identified the household as the primary setting influencing reported contact characteristics among our participants. The data in **Figure S27** indicate that reported household contact patterns exhibited a trend opposite to that of age-assortative mixing patterns. For both test-positive and test-negative populations, the reported number and duration of contacts within households did not show the high-intensity diagonal pattern typically seen in age-assortative mixing

as subject age changed. The distribution of the contact number and duration was relatively even across all age groups (**Figures S27D**, and **S27E**).

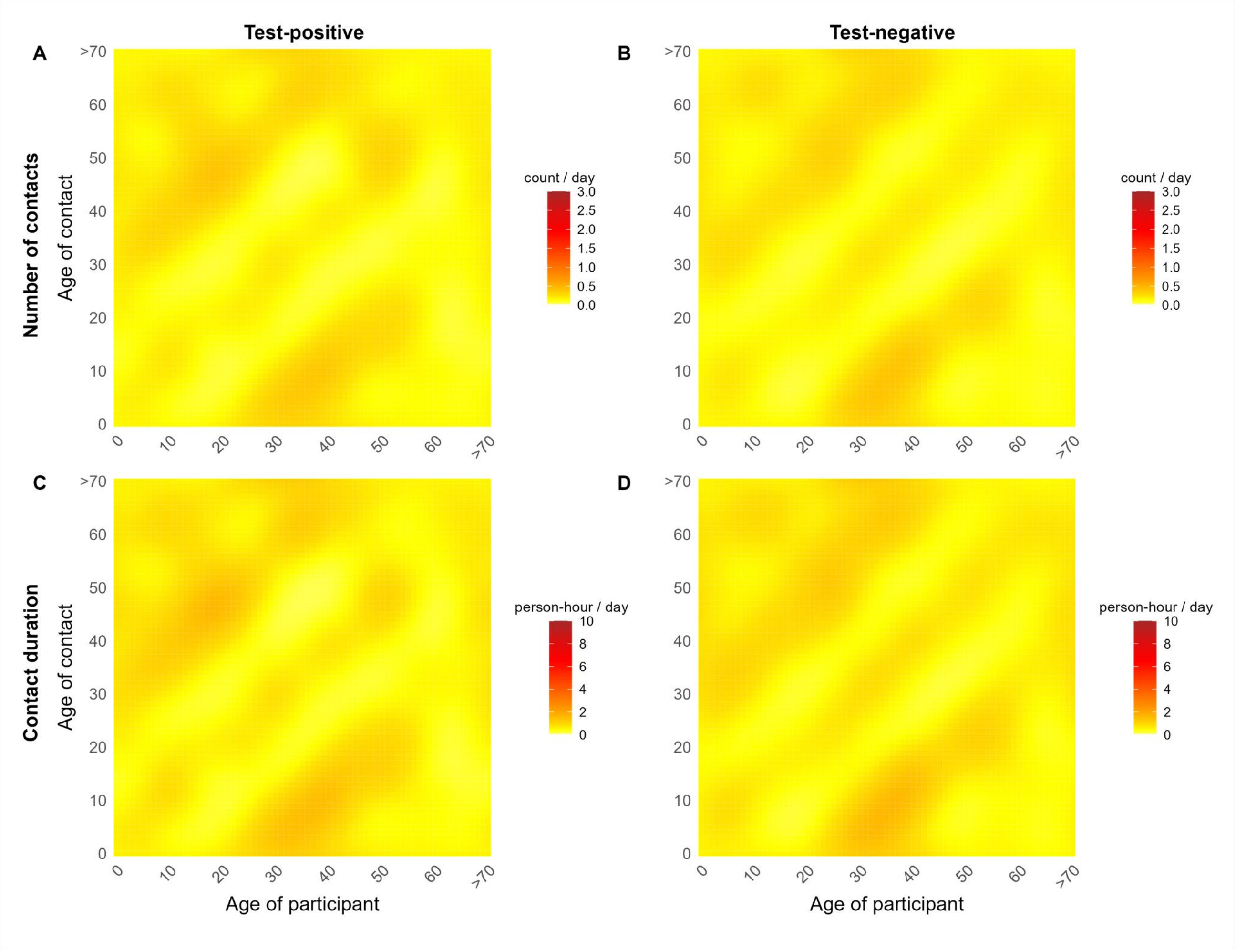

Figure S27. Age-specific contact matrix for **household settings**. All panels were presented and arranged as in Figure S17.

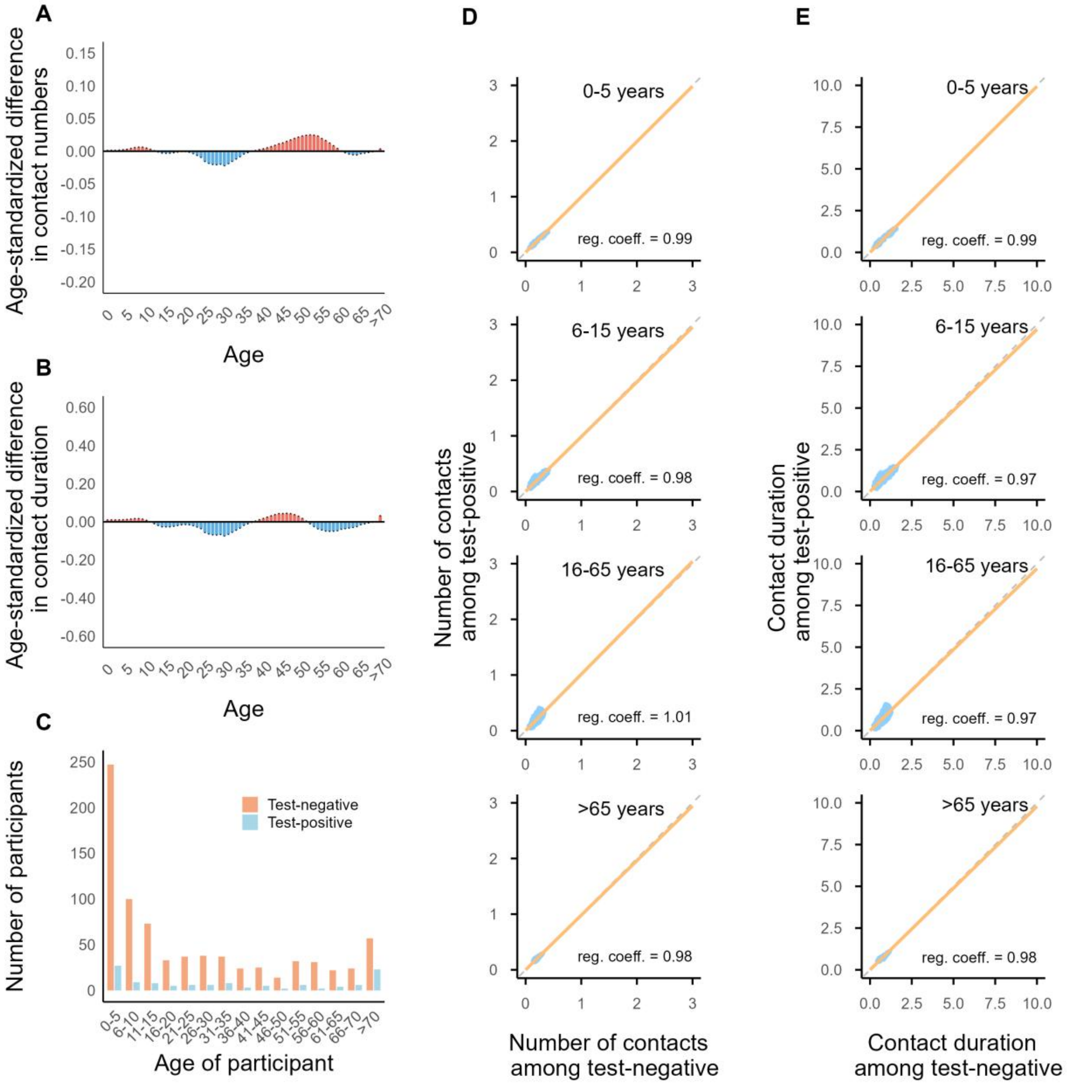

Figure S28. Contact patterns and testing outcomes by age group in **household settings**. All panels were presented and arranged as in Figure S18.

We found that schools and workplaces were settings with a relatively high reported contact density among our participants. As expected, reported contacts in schools predominantly occur among peers. Workplace contacts, while involving a broader age range, showed the highest intensity of peer interactions in the reported data. A distinct age-assortative mixing pattern was observed for both test-positive and test-negative subjects (**Figure S29**). However, the overall reported contact frequency and duration in these settings were below average, and the similarity in temporal contact patterns between positive and negative subjects was relatively low (**Figure S30**).

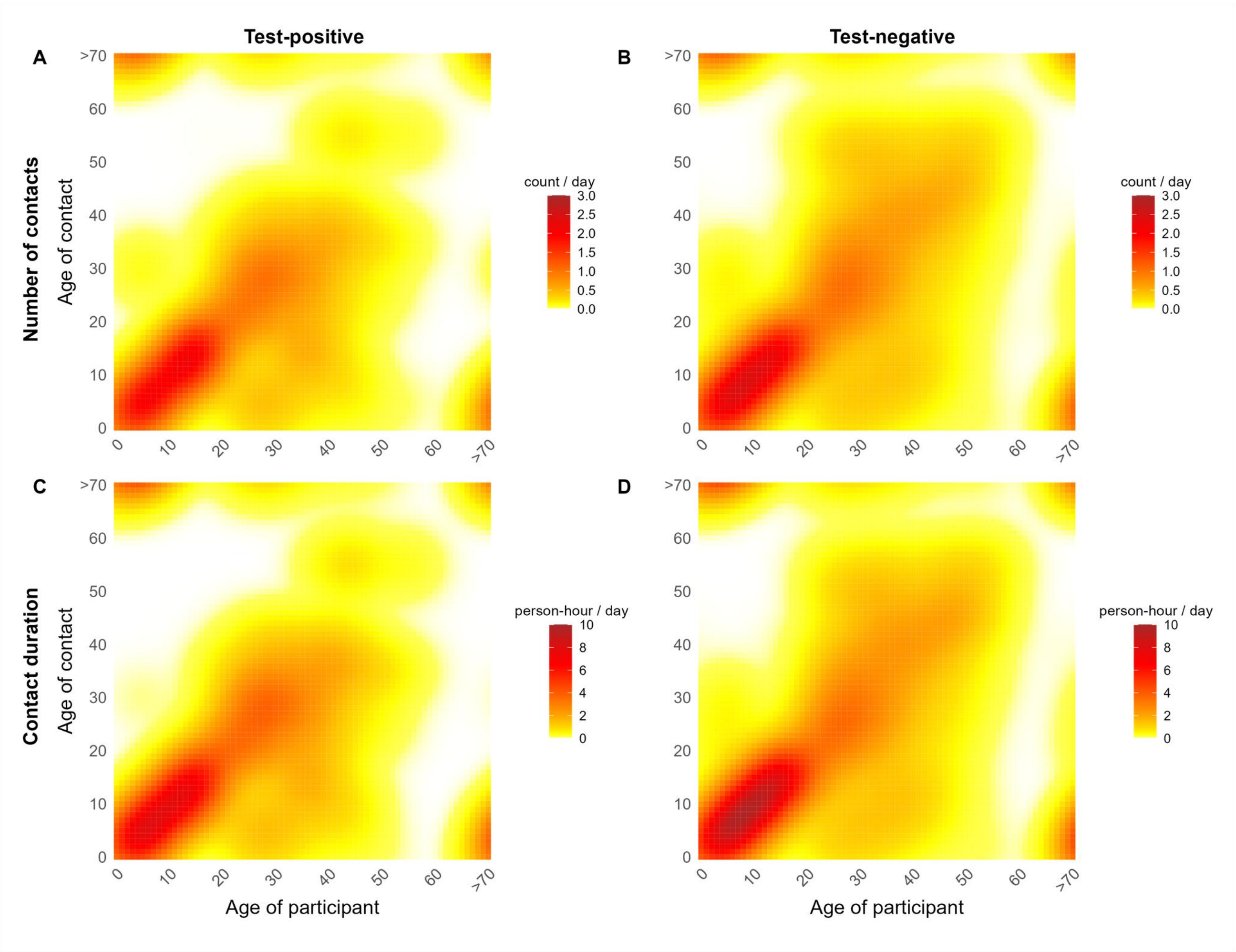

Figure S29. Age-specific contact matrix for **school and workplace settings**. All panels were presented and arranged as in Figure S17.

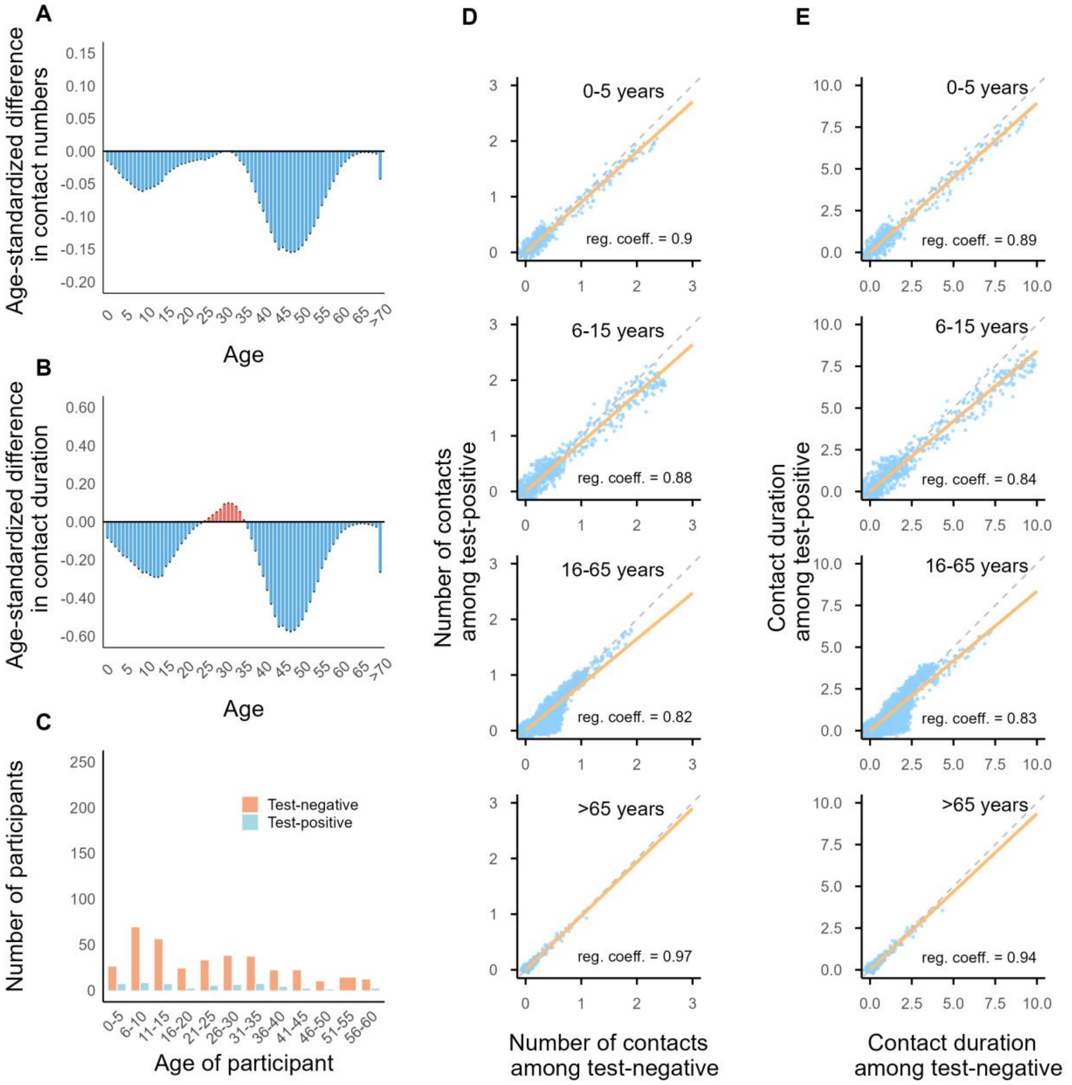

Figure S30. Contact patterns and testing outcomes by age group in **school and workplace settings**. All panels were presented and arranged as in Figure S18.

In leisure and other settings, we observed a highly pronounced age-assortative mixing pattern, as evidenced by a clear diagonal structure in the contact matrix (**Figure S31**). After age standardization, the overall contact frequency and duration reported by test-positive subjects were lower than those reported by test-negative subjects across most age groups, except for slightly higher values in the 0–10-year-old group. Regression analysis indicated that the association between age and contact measures differed in strength between test-positive and test-negative subjects across age groups. The strongest positive association for test-positive subjects was observed in the age group of 0–5 years, with a decreasing trend as age increased (**Figure S32**).

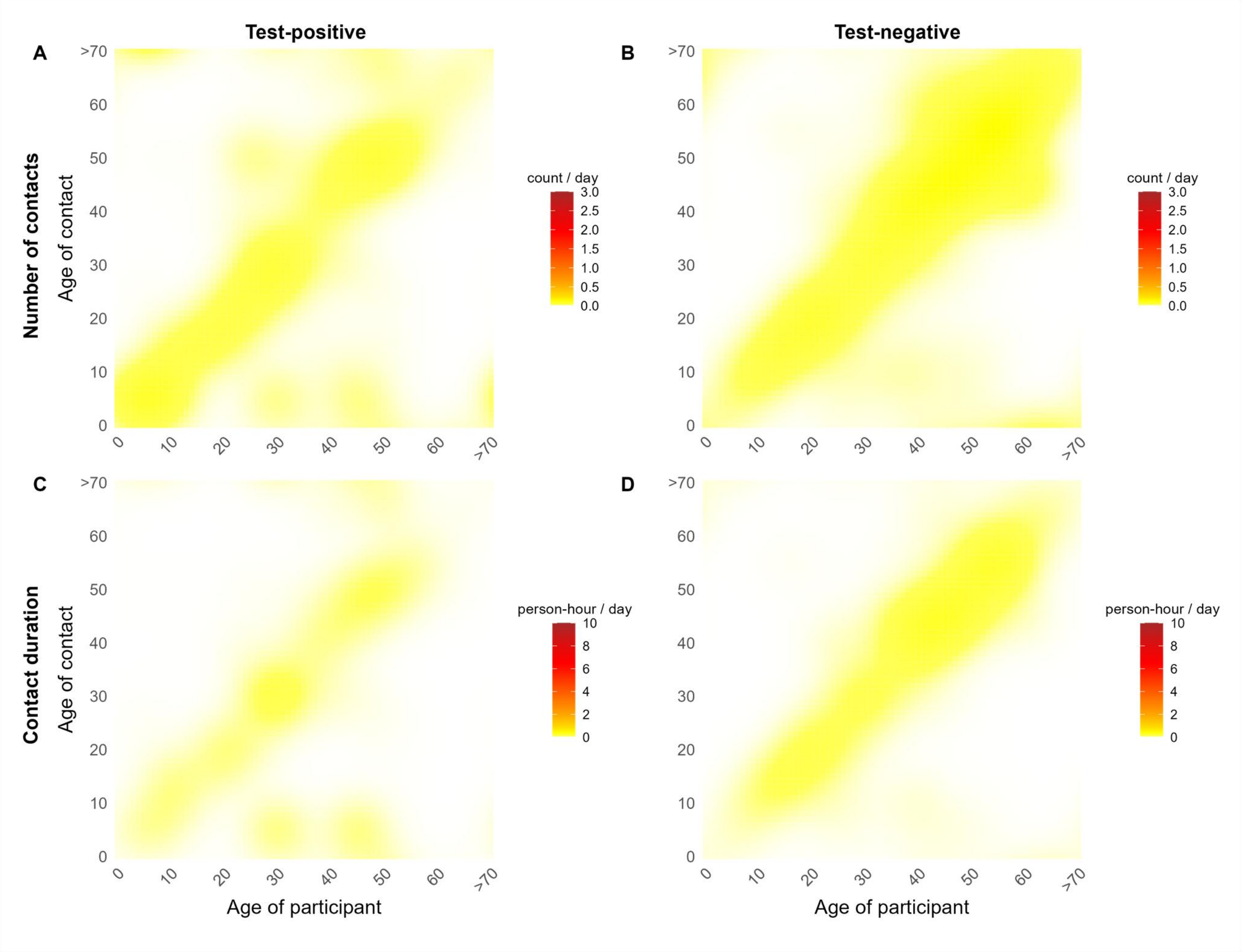

Figure S31. Age-specific contact matrix for **leisure and other settings**. All panels were presented and arranged as in Figure S17.

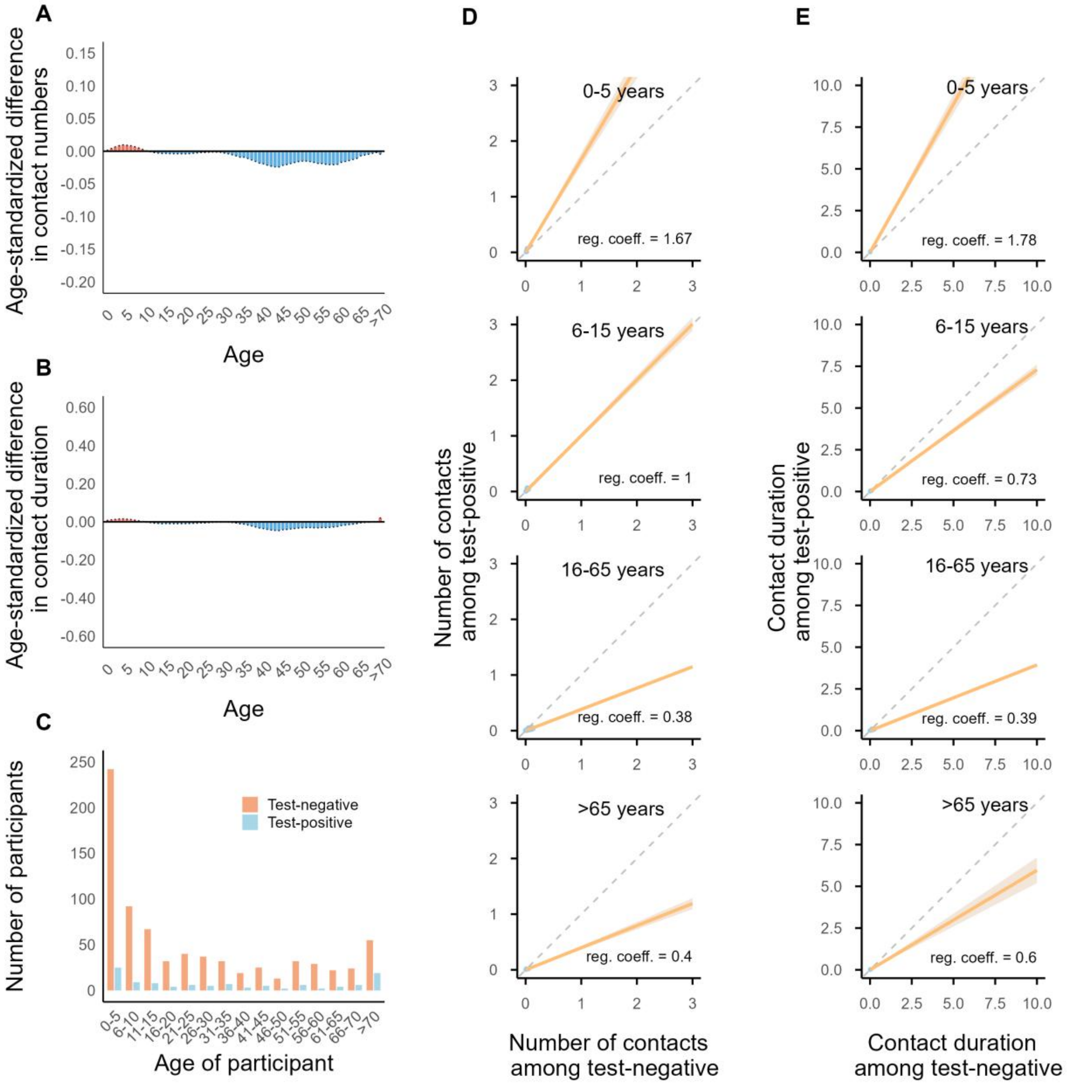

Figure S32. Contact patterns and testing outcomes by age group in **leisure and other settings**. All panels were presented and arranged as in Figure S18.

The three core settings exhibited distinct age-specific contact patterns among all healthcare-seeking ILI subjects. As the primary setting for reported contacts, households in our study did not exhibit the typical age-assortative mixing pattern, instead showing a relatively even distribution of contact frequency and duration across ages.In contrast, schools and workplaces exhibited clear age-assortative mixing patterns with peer-group clustering. However, the reported frequency and duration of contact did not exceed the study population's average, which is likely limited by structured routines (e.g., classroom schedules and the division of labour) that reduce opportunities for spontaneous contact in these populations.Leisure and other settings exhibited a strong age-assortative mixing pattern along the "age diagonal." Among subjects who tested positive, those in most age groups reported contact frequencies and durations below the overall subject average, except for children aged 0 to 10 years, whose reported levels were slightly elevated. The degree of concordance between positive and negative subject contacts varied substantially by age, being strongest in the 0–5-year-old age group and declining sharply with age.

S5. Sensitivity analysis

S5.1 Vaccine effectiveness

S5.1.1 Sensitivity analysis expanded to include children aged under 6 months

To assess robustness, we conducted several sensitivity analyses. The first assessed the impact of including infants under 6 months of age on the VE estimates. The primary analysis included 907 subjects (excluding children under 6 months), whereas this sensitivity analysis reincorporated these infants, increasing the total sample size to 928 subjects.

The analysis revealed that the estimated VE remained largely unchanged after the inclusion of infants under 6 months of age. The VE point estimates and 95% credible intervals for any influenza, influenza A, influenza B, and all subtypes (A/H1, A/H3, B/Victoria) were consistent with those of the primary analysis, indicating that the original model was insensitive to selection bias from the exclusion of this age group and demonstrating the robustness of the findings.

Specifically, the overall VE for any influenza was 42.2% (95% CrI: 11.0, 62.5), which is nearly identical to the estimate of 42.3% from the primary analysis. The VE point estimates and credible intervals across age strata and subtypes showed no material differences, further supporting the stability of the model estimates. Notably, the VE for children under 6 months could not be reliably estimated because of the very small number of vaccinations and subjects, but this had no significant effect on the overall estimates.

In summary, this sensitivity analysis indicates that excluding infants under 6 months of age did not introduce significant bias into the VE estimates, supporting the reliability of the primary analysis conclusions (see **Table S7**).

Table S7. Estimates of the effectiveness of influenza vaccines against influenza A and B (including infants younger than 6 months) stratified by age and influenza subtype.

	**Test-positive subjects**		**Test-negative subjects**		**VE** (95% CrI)
	Subtotal	Vaccinated (%)	Subtotal	Vaccinated (%)	
**Any influenza**					
Overall	122	5 (4.1%)	806	51 (6.3%)	42.2% (11.0, 62.5)
Stratified by ***age group***					
0-0.5 yr	1	0 (0.0%)	20	0 (0.0%)	100% (not estimated)[&]
0.5-6 yr	30	2 (6.7%)	239	15 (6.3%)	37.5% (-0.5, 61.1)
7-17 yr	15	0 (0.0%)	175	13 (7.4%)	100% (not estimated)[&]
18-59 yr	42	1 (2.4%)	262	12 (4.6%)	41.1% (5.4, 63.3)
≥60 yr	34	2 (5.9%)	110	11 (10.0%)	42.1% (7.8, 63.7)
**Influenza A**					
Overall	102	4 (3.9%)	806	51 (6.3%)	43.0% (11.6, 63.2)
Stratified by ***age group***					
0-0.5 yr	1	0 (0.0%)	20	0 (0.0%)	100% (not estimated)[&]
0.5-6 yr	24	2 (8.3%)	239	15 (6.3%)	36.5% (-2.3, 60.6)

7-17 yr	12	0 (0.0%)	175	13 (7.4%)	100% (not estimated)[&]
18-59 yr	33	1 (3.0%)	262	12 (4.6%)	40.4% (4.1, 63.0)
≥60 yr	32	1 (3.1%)	110	11 (10.0%)	44.5% (11.3, 65.2)
**Influenza B**					
Overall	20	1 (5.0%)	806	51 (6.3%)	39.0% (1.4, 62.2)
Stratified by ***age group***					
0-0.5 yr	0	0 (0.0%)	20	0 (0.0%)	100% (not estimated)[&]
0.5-6 yr	6	0 (0.0%)	239	15 (6.3%)	100% (not estimated)[&]
7-17 yr	3	0 (0.0%)	175	13 (7.4%)	100% (not estimated)[&]
18-59 yr	9	0 (0.0%)	262	12 (4.6%)	100% (not estimated)[&]
≥60 yr	2	1 (50.0%)	110	11 (10.0%)	36.9%(-2.7, 61.3)

& Adjusted VE is not shown for subgroups where the model could not be fitted due to the absence of vaccinated subjects in the test-positive group.

S5.1.2 Sensitivity analysis using age and BMI as continuous variables

The primary outcome model was adjusted for age and BMI as categorical variables to control for potential confounding effects. In the sensitivity analysis, we used the continuous form of age and BMI, fitting them via natural spline (ns) functions with 3 degrees of freedom (df).

The results of the analysis indicated that compared with the original model, converting age and BMI from categorical variables into continuous variables with spline terms did not significantly alter the overall VE estimate, and the credible intervals widely overlapped. The VE estimates and 95% CrIs for any influenza, influenza A, influenza B, and all subtypes (H1N1, H3, and Victoria) were highly consistent with those from the primary analysis. These findings demonstrate the robustness of the model to different mathematical handling of age and BMI and produce stable estimates.

In summary, this sensitivity analysis revealed that modelling age and BMI as either categorical variables or as continuous variables with splines did not substantially alter the core VE estimates, further supporting the robustness of the conclusions of the primary analysis (see **Table S8**).

Table S8. Estimates of influenza VE against influenza A and B (age and BMI as continuous variables with natural splines).

	**Test-positive subjects**		**Test-negative subjects**		**VE** (95% CrI)
	Subtotal	Vaccinated (%)	Subtotal	Vaccinated (%)	
**Any influenza**					
Overall	121	5 (4.1%)	786	51 (6.5%)	41.5% (9.9, 62.1)
Stratified by ***age group***					
0.5-6 yr	30	2 (6.7%)	239	15 (6.3%)	38.9% (2.1, 61.9)
7-17 yr	15	0 (0.0%)	175	13 (7.4%)	100% (not estimated)[&]

18-59 yr	42	1 (2.4%)	262	12 (4.6%)	41.0% (5.2, 63.3)
≥60 yr	34	2 (5.9%)	110	11 (10.0%)	41.7% (7.1, 63.4)
**Influenza A**					
Overall	101	4 (5.4%)	786	51 (6.5%)	42.0% (10.0, 62.6)
Stratified by ***age group***					
0.5-6 yr	24	2 (8.3%)	239	15 (6.3%)	37.6% (-0.2, 61.2)
7-17 yr	12	0 (0.0%)	175	13 (7.4%)	100% (not estimated)[&]
18-59 yr	33	1 (3.0%)	262	12 (4.6%)	40.2% (3.7, 62.9)
≥60 yr	32	1 (3.1%)	110	11 (10.0%)	44.3% (11.0, 65.1)
**Influenza B**					
Overall	20	1 (5.0%)	786	51 (6.5%)	39.3% (2.1, 62.4)
Stratified by ***age group***					
0.5-6 yr	6	0 (0.0%)	239	15 (6.3%)	100% (not estimated)[&]
7-17 yr	3	0 (0.0%)	175	13 (7.4%)	100% (not estimated)[&]
18-59 yr	9	0 (0.0%)	262	12 (4.6%)	100% (not estimated)[&]
≥60 yr	2	1 (50.0%)	110	11 (10.0%)	36.9%(-2.9, 61.2)

& Adjusted VE is not shown for subgroups where the model could not be fitted due to the absence of vaccinated subjects in the test-positive group.

S5.1.3 Sensitivity analysis incorporating the calendar date

In addition to the variables adjusted for in the primary model (sex, time of presentation, age group, and BMI group), we further included the calendar date of symptom onset to evaluate potential confounding by temporal trends. This variable was modelled as a continuous variable via a natural spline (ns, df=3).

After adjusting for calendar time, the point estimate of VE was 44.2% (95% CrI: 14.0, 63.7). This value is slightly higher than the 42.3% (95% CrI: 11.1, 62.5) observed in the primary analysis, and the lower limit of the confidence interval was also higher. Consistent modest increases were observed in the VE estimates against any influenza, influenza A, influenza B, and each subtype. These findings suggest that unadjusted calendar time may have introduced a slight negative confounding effect and that controlling for it yielded more precise and robust estimates. The results from both the primary and sensitivity analyses mutually corroborate the core conclusion that the vaccine provides protective efficacy (see **Table S9**).

Table S9. Estimates of influenza VE against influenza A and B (additionally adjusted for the calendar time of symptom onset via natural splines).

	**Test-positive subjects**		**Test-negative subjects**		**VE** (95% CrI)
	Subtotal	Vaccinated (%)	Subtotal	Vaccinated (%)	
**Any influenza**					

Overall	121	5 (4.1%)	786	51 (6.5%)	44.2% (14.0, 63.7)
Stratified by ***age group***					
0.5-6 yr	30	2 (6.7%)	239	15 (6.3%)	40.0% (0.3, 61.4)
7-17 yr	15	0 (0.0%)	175	13 (7.4%)	100% (not estimated)
18-59 yr	42	1 (2.4%)	262	12 (4.6%)	41.1% (5.4, 63.4)
≥60 yr	34	2 (5.9%)	110	11 (10.0%)	42.0% (7.5, 63.7)
**Influenza A**					
Overall	101	4 (5.4%)	786	51 (6.5%)	44.6% (14.1, 64.2)
Stratified by ***age group***					
0.5-6 yr	24	2 (8.3%)	239	15 (6.3%)	37.1% (-1.3, 61.0)
7-17 yr	12	0 (0.0%)	175	13 (7.4%)	100% (not estimated)[&]
18-59 yr	33	1 (3.0%)	262	12 (4.6%)	40.5% (4.3, 63.1)
≥60 yr	32	1 (3.1%)	110	11 (10.0%)	44.1% (10.5, 65.1)
**Influenza B**					
Overall	20	1 (5.0%)	786	51 (6.5%)	39.2% (1.8, 62.3)
Stratified by ***age group***					
0.5-6 yr	6	0 (0.0%)	239	15 (6.3%)	100% (not estimated)[&]
7-17 yr	3	0 (0.0%)	175	13 (7.4%)	100% (not estimated)[&]
18-59 yr	9	0 (0.0%)	262	12 (4.6%)	100% (not estimated)[&]
≥60 yr	2	1 (50.0%)	110	11 (10.0%)	37.2%(-2.3, 61.4)

& Adjusted VE is not shown for subgroups where the model could not be fitted due to the absence of vaccinated subjects in the test-positive group.

S5.2 Contact patterns

To evaluate whether vaccination status significantly modified the association between contact and influenza test results and to test the robustness of our main findings, we performed a sensitivity analysis limited to unvaccinated subjects (n=872) and excluded those who were vaccinated (n=56).

As shown in **Figure S33** and **Figure S34**, the contact patterns remained consistent with the main findings after the exclusion of vaccinated subjects. Distinct diagonal and subdiagonal patterns are still evident in **Figure S33**. The regression coefficients in **Figure S34D** and **S34E** differed by <0.05 from their counterparts in the primary analysis. These results confirm that the association between contact and influenza test outcomes is highly consistent with that observed in the full cohort. The core relationship remains substantively unchanged, indicating that the original conclusions are robust to and unconfounded by vaccination status. This results confirm that the association persists after accounting for the direct protective effect of vaccination, thereby underscoring its high reliability.

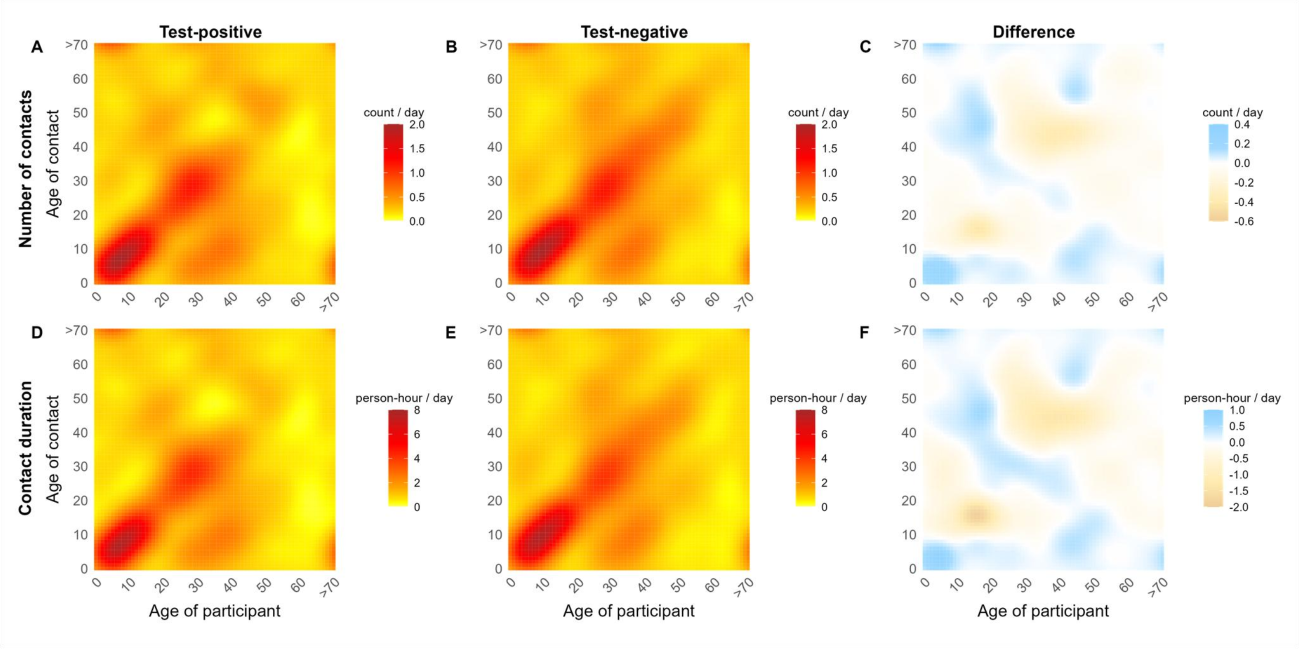

Figure S33. Age-specific contact matrix, **excluding the vaccinated group**. All panels were presented and arranged as in Figure S17.

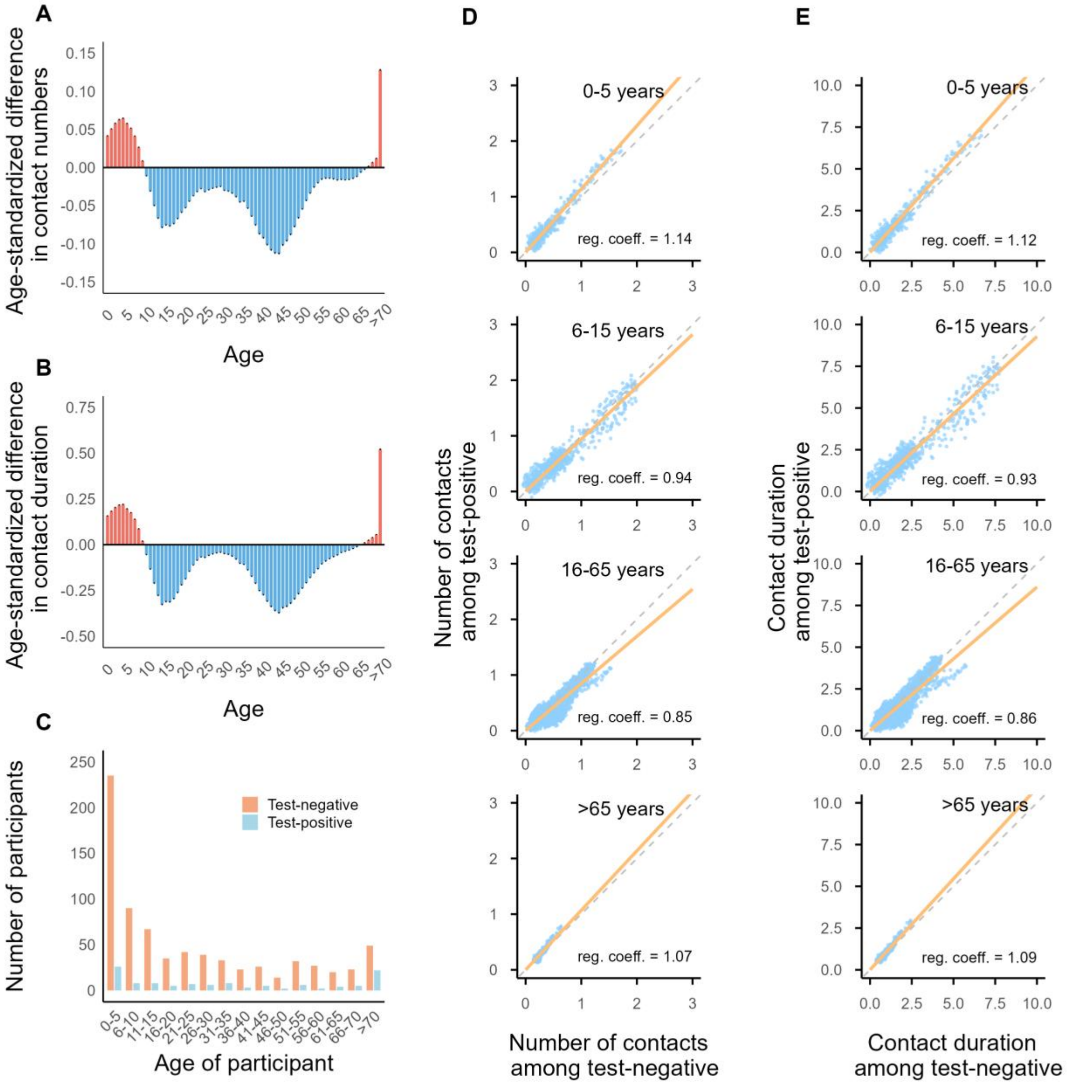

Figure S34. Contact patterns and testing outcomes by age group, **excluding the vaccinated group**. All panels were presented and arranged as in Figure S18.

Supplementary references

[1] World Health Organization. Surveillance and monitoring: case definitions for ILI and SARI. World Health Organization. Accessed October 4, 2025. https://www.who.int/teams/global-influenza-programme/surveillance-and-monitoring/case-definitions-for-ili-and-sari.

[2] (in Simplified Chinese) Chinese Center for Disease Control and Prevention. National Influenza Surveillance Program (2017 version). Published 2017. Accessed October 4, 2025. https://ivdc.chinacdc.cn/cnic/zyzx/jcfa/201709/t20170927_153830.htm.

[3] Wang Z, Yang P, Wang R, et al. Estimating the contribution of setting-specific contacts to SARS-CoV-2 transmission using digital contact tracing data. Nat Commun. 2024;15(1):6103. Published 2024 Jul 19.

[4] Taherdoost, H. (2022). Designing a Questionnaire for a Research Paper: A Comprehensive Guide to Design and Develop an Effective Questionnaire. Asian Journal of Managerial Science, 11(1), 8–16.

[5] Danecek P, Bonfield JK, Liddle J, et al. Twelve years of SAMtools and BCFtools. Gigascience. 2021;10(2):giab008.

[6] Van der Auwera GA & O'Connor BD. (2020). Genomics in the Cloud: Using Docker, GATK, and WDL in Terra (1st Edition). O'Reilly Media.

[7] Kumar S, Stecher G, Suleski M, Sanderford M, Sharma S, Tamura K. MEGA12: Molecular Evolutionary Genetic Analysis Version 12 for Adaptive and Green Computing. Mol Biol Evol. 2024;41(12):msae263.

[8] McLean HQ, Belongia EA. Influenza Vaccine Effectiveness: New Insights and Challenges. Cold Spring Harb Perspect Med. 2021;11(6):a038315. Published 2021 Jun 1.

[9] Gelman, A., & Su, Y. (2024). arm: Data Analysis Using Regression and Multilevel/Hierarchical Models (Version 1.14-4) [R package]. https://CRAN.R-project.org/package=arm

[10] Khare, S., et al (2021) GISAID's Role in Pandemic Response. China CDC Weekly, 3(49): 1049-1051.

[11] Thomas K.F. Wong, Nhan Ly-Trong, Huaiyan Ren, Hector Banos, Andrew J. Roger, Edward Susko, Chris Bielow, Nicola De Maio, Nick Goldman, Matthew W. Hahn, Gavin Huttley, Robert Lanfear, Bui Quang Minh (2025) IQ-TREE 3: Phylogenomic Inference Software using Complex Evolutionary Models. Submitted. https://ecoevorxiv.org/repository/view/8916/

[12] Yu G. (2020). Using ggtree to Visualize Data on Tree-Like Structures. Current protocols in bioinformatics, 69(1), e96. https://doi.org/10.1002/cpbi.96.

[13] The PyMOL Molecular Graphics System, Version 3.0 Schrödinger, LLC.

[14] Xu R, Ekiert DC, Krause JC, Hai R, Crowe JE Jr, Wilson IA. Structural basis of preexisting immunity to the 2009 H1N1 pandemic influenza virus. Science. 2010;328(5976):357-360.

[15] Stadlbauer D, Zhu X, McMahon M, et al. Broadly protective human antibodies that target the active site of influenza virus neuraminidase. Science. 2019;366(6464):499-504.

[16] Berman H, Henrick K, Nakamura H. Announcing the worldwide Protein Data Bank. Nat Struct Biol. 2003;10(12):980.

[17] Cao L, Lou J, Zhao S, et al. In silico prediction of influenza vaccine effectiveness by sequence analysis. Vaccine. 2021;39(7):1030-1034.

[18] World Health Organization. Recommended composition of influenza virus vaccines for use in the 2023-2024 northern hemisphere influenza season. World Health Organization. Accessed 24 February 2023.
https://www.who.int/publications/m/item/recommended-composition-of-influenza-virus-vaccines-for-use-in-the-2023-2024-northern-hemisphere-influenza-season.

[19] Zhang J, Klepac P, Read JM, et al. Patterns of human social contact and contact with animals in Shanghai, China. Sci Rep. 2019;9(1):15141.

[20] Mossong J, Hens N, Jit M, et al. Social contacts and mixing patterns relevant to the spread of infectious diseases. PLoS Med. 2008;5(3):e74.

[21] Leung K, Jit M, Lau EHY, Wu JT. Social contact patterns relevant to the spread of respiratory infectious diseases in Hong Kong. Sci Rep. 2017;7(1):7974.

STROBE Statement—checklist of items that should be included in reports of observational studies

	Item No.	Recommendation	Page No.
**Title and abstract**	1	(*a*) Indicate the study's design with a commonly used term in the title or the abstract	1
		(*b*) Provide in the abstract an informative and balanced summary of what was done and what was found	3
**Introduction**			
Background/rationale	2	Explain the scientific background and rationale for the investigation being reported	6
Objectives	3	State specific objectives, including any prespecified hypotheses	6, 7
**Methods**			
Study design	4	Present key elements of study design early in the paper	7
Setting	5	Describe the setting, locations, and relevant dates, including periods of recruitment, exposure, follow-up, and data collection	7
Participants	6	(*a*) *Cohort study*—Give the eligibility criteria, and the sources and methods of selection of participants. Describe methods of follow-up<br>*Case-control study*—Give the eligibility criteria, and the sources and methods of case ascertainment and control selection. Give the rationale for the choice of cases and controls<br>*Cross-sectional study*—Give the eligibility criteria, and the sources and methods of selection of participants	7
		(*b*) *Cohort study*—For matched studies, give matching criteria and number of exposed and unexposed<br>*Case-control study*—For matched studies, give matching criteria and the number of controls per case	NA
Variables	7	Clearly define all outcomes, exposures, predictors, potential confounders, and effect modifiers. Give diagnostic criteria, if applicable	7, 8
Data sources/ measurement	8*	For each variable of interest, give sources of data and details of methods of assessment (measurement). Describe comparability of assessment methods if there is more than one group	*7,8*
Bias	9	Describe any efforts to address potential sources of bias	8
Study size	10	Explain how the study size was arrived at	7

Continued on next page

Quantitative variables	11	Explain how quantitative variables were handled in the analyses. If applicable, describe which groupings were chosen and why	NA
Statistical methods	12	(*a*) Describe all statistical methods, including those used to control for confounding	8
		(*b*) Describe any methods used to examine subgroups and interactions	8, 9
		(*c*) Explain how missing data were addressed	NA
		(*d*) *Cohort study*—If applicable, explain how loss to follow-up was addressed<br>*Case-control study*—If applicable, explain how matching of cases and controls was addressed<br>*Cross-sectional study*—If applicable, describe analytical methods taking account of sampling strategy	NA
		(*e*) Describe any sensitivity analyses	9
Participants	13*	(a) Report numbers of individuals at each stage of study—eg numbers potentially eligible, examined for eligibility, confirmed eligible, included in the study, completing follow-up, and analysed	9
		(b) Give reasons for non-participation at each stage	9
		(c) Consider use of a flow diagram	NA
Descriptive data	14*	(a) Give characteristics of study participants (eg demographic, clinical, social) and information on exposures and potential confounders	9
		(b) Indicate number of participants with missing data for each variable of interest	NA
		(c) *Cohort study*—Summarise follow-up time (eg, average and total amount)	NA
Outcome data	15*	*Cohort study*—Report numbers of outcome events or summary measures over time	NA
		Case-control study—Report numbers in each exposure category, or summary measures of exposure	9
		Cross-sectional study—Report numbers of outcome events or summary measures	NA
Main results	16	(*a*) Give unadjusted estimates and, if applicable, confounder-adjusted estimates and their precision (eg, 95% confidence interval). Make clear which confounders were adjusted for and why they were included	9, 10
		(*b*) Report category boundaries when continuous variables were categorized	9
		(*c*) If relevant, consider translating estimates of relative risk into absolute risk for a meaningful time period	NA

Continued on next page

Other analyses	17	Report other analyses done—eg analyses of subgroups and interactions, and sensitivity analyses	10, 11, 12
Key results	18	Summarise key results with reference to study objectives	10, 11
Limitations	19	Discuss limitations of the study, taking into account sources of potential bias or imprecision. Discuss both direction and magnitude of any potential bias	12, 13
Interpretation	20	Give a cautious overall interpretation of results considering objectives, limitations, multiplicity of analyses, results from similar studies, and other relevant evidence	12, 13
Generalisability	21	Discuss the generalisability (external validity) of the study results	13
**Other information**			
Funding	22	Give the source of funding and the role of the funders for the present study and, if applicable, for the original study on which the present article is based	14

*Give information separately for cases and controls in case-control studies and, if applicable, for exposed and unexposed groups in cohort and cross-sectional studies.

Note: An Explanation and Elaboration article discusses each checklist item and gives methodological background and published examples of transparent reporting. The STROBE checklist is best used in conjunction with this article (freely available on the Web sites of PLoS Medicine at http://www.plosmedicine.org/, Annals of Internal Medicine at http://www.annals.org/, and Epidemiology at http://www.epidem.com/). Information on the STROBE Initiative is available at www.strobe-statement.org.
